# High Coverage Sub‐Nano Iridium Cluster on Core–Shell Cobalt‐Cerium Bimetallic Oxide for Highly Efficient Full‐pH Water Splitting

**DOI:** 10.1002/advs.202407475

**Published:** 2024-10-14

**Authors:** Lili Zhang, Yuanting Lei, Yinze Yang, Dan Wang, Yafei Zhao, Xu Xiang, Huishan Shang, Bing Zhang

**Affiliations:** ^1^ School of Chemical Engineering Zhengzhou Key Laboratory of Advanced Separation Technology Zhengzhou University Zhengzhou 450001 P. R. China; ^2^ State Key Laboratory of Chemical Resource Engineering Beijing University of Chemical Technology Beijing 100029 P. R. China

**Keywords:** cobalt‐cerium bimetallic oxide, core–shell, full‐pH water splitting, high coverage, sub‐nano iridium cluster

## Abstract

The construction of sub‐nanometer cluster catalysts (<1 nm) with almost complete exposure of active atoms serves as a promising avenue for the simultaneous enhancement of atom utilization efficiency and specific activity. Herein, a core–shell cobalt‐cerium bimetallic oxide protected by high coverage sub‐nanometer Ir clusters (denoted as Ir cluster@CoO/CeO_2_) is constructed by a confined in situ exsolution strategy. The distinctive core–shell structure endows Ir cluster@CoO/CeO_2_ with enhanced intrinsic activity and high conductivity, facilitating efficient charge transfer and full‐pH water splitting. The Ir cluster@CoO/CeO_2_ achieves low overpotentials of 49/215, 52/390, and 54/243 mV at 10 mA cm^−2^ for hydrogen evolution reaction/oxygen evolution reaction (HER/OER) in 0.5 m H_2_SO_4_, 1.0 m PBS, and 1.0 m KOH, respectively. The small decline in performance after 300 h of operation renders it one of the most effective catalysts for full‐pH water splitting. DFT calculations indicate that oriented electron transfer (along the path from Ce to Co and then to Ir) creates an electron‐rich environment for surface Ir clusters. The reconstructed interface electronic environment provides optimized intermediates adsorption/desorption energy at the Ir site (for HER) and at the Ir‐Co site (for OER), thus simultaneously speeding up the HER/OER kinetics.

## Graphical abstract

Designed a uniform high coverage sub nano iridium cluster catalyst supported on core–shell bimetallic oxides (Ir cluster@CoO/CeO_2_). Due to interface electron rearrangement and stable heterostructure, the catalyst exhibits excellent electrochemical water‐splitting performance across the entire pH range.

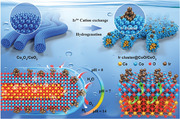

## 1 Introduction

To obtain a large and sustainable supply of green hydrogen energy, an in‐depth exploration of highly efficient catalysts in electrocatalytic water splitting has always been the research focus.^[^ ^1^, ^2^ ^]^ Pt, Ru/Ir‐based catalysts as the benchmark hydrogen/oxygen evolution reaction (HER/OER) catalysts for electrocatalytic water splitting have been extensively studied due to their high intrinsic activity.^[^ ^3^ ^]^ Pt‐based catalysts are widely developed as cathode materials, unfortunately, it is difficult to achieve complete electrocatalytic water splitting because they are easily oxidized to PtO_x_ and deactivated in OER.^[^ ^4^ ^]^ Ru‐based catalysts have outstanding performance for OER, but the soluble high oxidation state Ru (such as RuO_4_) formed during the OER process can suffer from rapid degradation under acidic and oxidation conditions, resulting in its poor stability.^[^ ^5^ ^]^ These limitations greatly increase the difficulty of further developing Pt‐based and Ru‐based bifunctional electrocatalysts under full‐pH conditions.^[^ ^6^ ^]^ In contrast, Ir‐based catalysts are expected to become the most versatile catalysts for water splitting, but the downside is that iridium is extremely scarce with a tenfold smaller abundance than platinum.^[^ ^7^ ^]^ Considering the complex microenvironment of water splitting over a wide pH range (inevitable changes of proton concentration),^[^ ^8^ ^]^ manufacturing Ir‐based bifunctional electrocatalysts with sufficient activity, a high utilization rate, and a clear mechanism remains a long‐term goal.

Downsizing the metal species to precise atom levels is a valid strategy to elevate metal utilization and control costs.^[^ ^9^ ^]^ Sub‐nano cluster catalysts are typically composed of atomically precise and isolated metal clusters, whose diameter is no more than 1 nanometer and contain 2–20 precise separation atoms.^[^ ^10^ ^]^ The sub‐nano cluster catalysts are located in the middle position between emerging single‐atom catalysts and traditional metal nanoparticle catalysts.^[^ ^11^ ^]^ Single‐atom catalysts have high activity/selectivity, but their ability is limited by the geometric constraints of isolated metal centers, which means they cannot simultaneously accelerate reactions involving multiple reactant molecules/intermediates and multi‐step redox processes.^[^ ^12^ ^]^ Traditional metal nanoparticle catalysts have unsatisfactory durability due to their easy solubility during the reaction process.^[^ ^13^ ^]^ Comparatively, sub‐nano cluster catalysts have more advantages in catalysis due to their surface geometric properties, quantum size effects, and unique electronic structure.^[^ ^14^, ^15^ ^]^ Considering the significant impact of surface effects on nano‐catalysts, precise control of the size, arrangement, and combination of metal clusters directly determines their activity, selectivity, and stability in electrocatalytic applications.^[^ ^16^ ^]^ Additionally, industrial applications necessitate a substantial density of metal atoms on durable supports. When each metal atom exhibits high catalytic activity, the yield of the desired product per unit volume or mass of catalyst is optimized. Consequently, the preparation of sub‐nano cluster catalysts with high coverage and uniform distribution has the potential to motivate excellent performance in electrocatalytic water splitting but is challenging.

Although bountiful research has been made to synthesize sub‐nano clusters, difficulties remain in controlling the distribution of metal clusters on designated support materials, particularly with high metal loading. Searching for a practicable strategy to prevent metal clusters from aggregating into nanoparticles during synthesis and reaction processes remains the research focus. Since the support surface properties play an integral role in the adsorption/dispersion of metal precursor molecules and the stability of generated clusters.^[^ ^17^ ^]^ Therefore, designing appropriate support materials that enable highly stabilized and interact with metal clusters to prevent metal aggregation is one of the breakthroughs.^[^ ^18^ ^]^ Graphite nitride carbon with plentiful nitrogen atom‐modified coordination sites has been commonly utilized as a support material for sub‐nano metal clusters. Whereas, the high mobility of metal atoms at high temperatures can easily lead to metal aggregation, resulting in loss of positional uniformity.^[^ ^19^ ^]^ As support for sub‐nano metal clusters, metal oxides can provide good structural stability under alkaline conditions, but their durability in catalyzing water splitting under acidic and neutral conditions is insufficient.^[^ ^20^ ^]^ In addition, the implementation of a core–shell structure has the potential to optimize the transport‐reaction trade‐offs and effectively utilize catalytically active material, thereby improving the catalytic performance of the catalyst. Considering the influence of microenvironmental factors during the synthesis process, reasonably customizing catalysts with optimal composition and configuration is the infallible direction for achieving high‐coverage metal cluster catalysts.

Herein, we elaborately designed high coverage sub‐nano Ir clusters on core–shell CoO/CeO_2_ nanowire (denoted as Ir cluster@CoO/CeO_2_) for efficient and stable full‐pH water splitting. The uniformly covered Ir clusters on the outermost layer effectively enhance the corrosion resistance of the catalyst in various microenvironments, inheriting the high activity of Ir‐based catalyst while simultaneously greatly improving stability. The CeO_2_ core functions as an electron donor to stabilize the electronic environment at the catalyst interface, while the CoO shell effectively anchors sub‐nano Ir clusters through strong electronic interactions and synergistically catalyzes the reaction via Ir─Co bonds. The optimization in composition and structure endow Ir cluster@CoO/CeO_2_ catalyst with excellent water splitting activity at the full‐pH range. In addition, benefiting from the maximum atomic utilization of sub‐nano Ir clusters (≈0.7 nm), the catalyst has a high turnover value and mass activity. Theoretical calculations reveal that the Ir site has the optimal free energy of H adsorption, achieving an accelerated hydrogen evolution rate. For OER, the Ir‐Co diatomic sites synergistically regulate the electronic structure and optimize the adsorption/desorption of oxygen‐containing intermediates, thereby enhancing OER.

## 2 Results and Discussion

**Figure 1:**
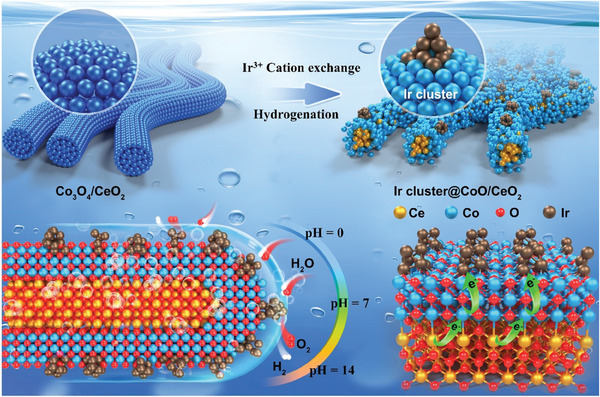
Schematic diagram of the synthesis of the core–shell Ir cluster@CoO/CeO_2_.

As illustrated in **Figures** **1** and S1 (Supporting Information), the Ir cluster@CoO/CeO_2_ was fabricated via an in situ exsolution strategy. First, the bimetallic oxide Co_3_O_4_/CeO_2_ heterostructure was prepared via solvothermal and calcination processes. Then, Co_3_O_4_/CeO_2_ was soaked in a low concentration of Ir^3+^ solution to undergo a cation exchange reaction to form Ir^3+^@Co_3_O_4_/CeO_2_ heterostructure as the host oxide. According to Harrison's classification, metal ions undergo simultaneous diffusion into both the bulk lattice and the grain boundaries at elevated temperatures. However, when the homologous temperature decreases to approximately one‐third of the melting point, diffusion is restricted solely to the grain boundaries.^[^ ^21^ ^]^ Considering that the melting point of CeO_2_ is 2475 °C, it remains stable at lower reduction temperatures.^[^ ^22^ ^]^ Co serves as the exsolving cation due to its elevated cosegregation energy associated with the exsolution process. Consequently, in a reducing environment, the Co ion is liberated from the hetero‐interface and migrates to the surface of the oxide. The Ir^3+^@Co_3_O_4_/CeO_2_ experienced exsolution and phase transition, which was in situ reconstructed into the Ir cluster@CoO/CeO_2_. In detail, the in situ exsolution of Co ions impelled the formation of a core–shell structure, which still maintained the heterogeneous interfaces by the atomic connectivity of the Co─O─Ce network for efficient electron conduction.^[^ ^23^ ^]^ It is noteworthy that the catalyst underwent a remarkable phase transition, accompanied by a change in morphology and roughness. This may result in the exposure of more active sites during the reaction process. By adjusting the concentration of Ir ions, high‐coverage sub‐nano Ir clusters were obtained, which were uniformly embedded on the surface of bimetallic oxides. The high‐coverage sub‐nanometer iridium clusters on the surface create a protective barrier that inhibits the further reduction of CoO to lower valence Co species. Additionally, these clusters safeguard the cobalt sites by forming Ir‐Co bonds, thereby preventing their dissolution during the reaction, particularly in acidic environments. The core–shell structure not only possessed excellent activity but also provided more guarantee for the stability of the catalyst.

**Figure 2:**
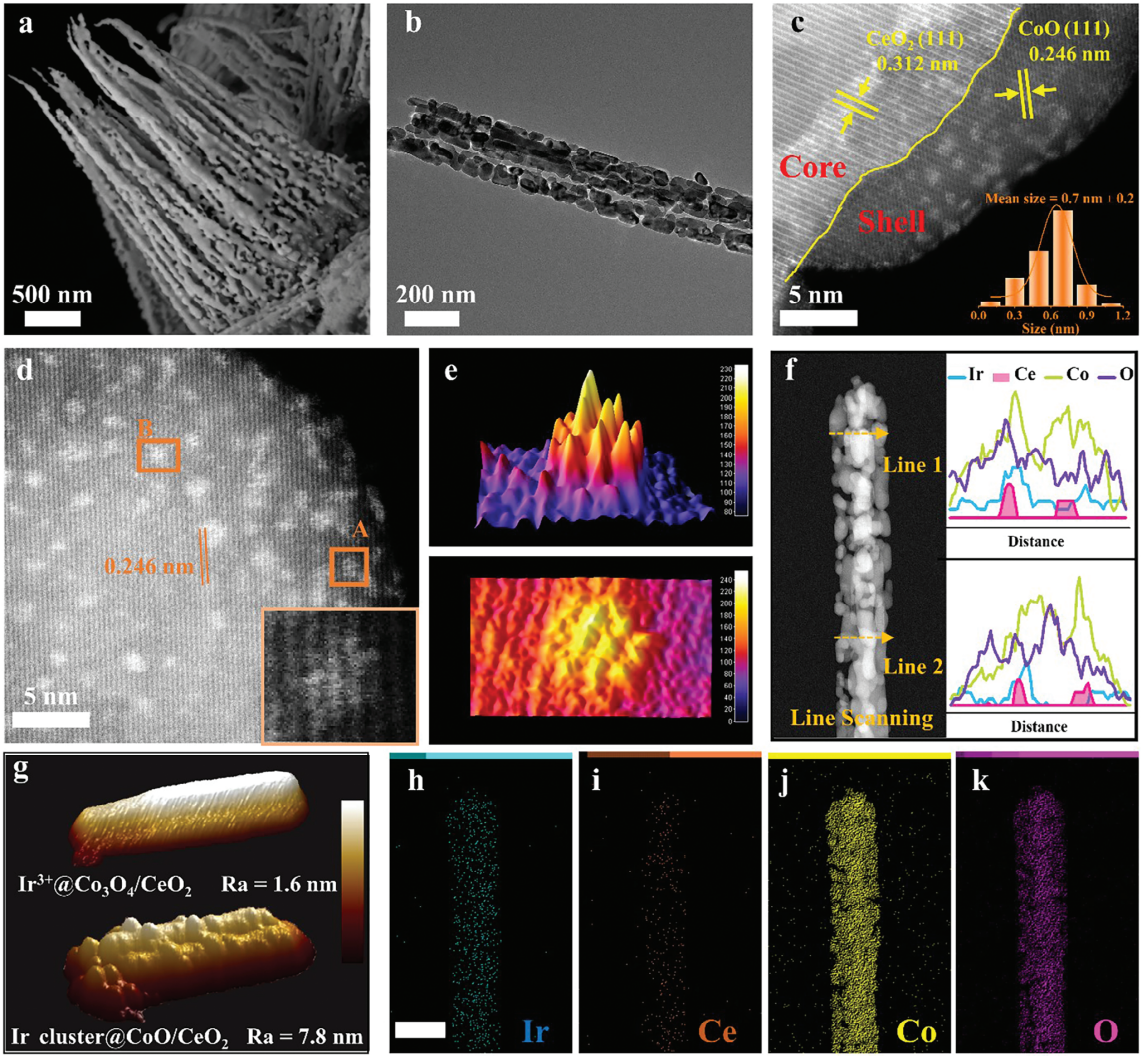
Morphology characterization of Ir cluster@CoO/CeO_2_. a) SEM, b) TEM, and c) Aberration‐corrected HAADF‐STEM images. The inset of (c): histogram of the size distribution of Ir nanoclusters. d) AC HAADF‐STEM image of Ir cluster@CoO/CeO_2_. The inset of (d): Magnified AC HAADF‐STEM image of region A. e) 3D intensity surface plot and intensity range displayed for the regions B in (d). f) HAADF‐STEM image (left) and the EDS line scanning (right) along Line 1 and Line 2 of Ir cluster@CoO/CeO_2_ nanowire. g) 3D modes of AFM images for Ir^3+^@Co_3_O_4_/CeO_2_ and Ir cluster@CoO/CeO_2_. h–k) Corresponding element mapping of Ir cluster@CoO/CeO_2_. The scale bar is 200 nm.

To investigate the nanostructure of Ir cluster@CoO/CeO_2_, scanning and transmission electron microscopies (SEM and TEM) were performed. As displayed in **Figures** **2**a and S2 (Supporting Information), Ir cluster@CoO/CeO_2_ exhibited a dense array of nanowires with orderly arrangement and rough surface. The nanowires with an approximate length of 6 µm were vertically grown on carbon cloth forming a 3D configuration. This 3D topological structure was in favor of maximizing the exposure of active surface, offering a highly accessible mass transport pathway and facilitating the diffusion of reaction intermediates.^[^ ^24^ ^]^ The TEM image revealed that the Ir cluster@CoO/CeO_2_ nanowire had a diameter of ≈100 nm (Figure 2b). The heterogeneous interfaces between CoO and CeO_2_ could be intuitively observed in the HRTEM image in Figure 2c. Interestingly, the lattice of CeO_2_ (with a lattice spacing of 0.312 nm, attributing to the (111) plane) was surrounded by the lattice of CoO (with a lattice spacing of 0.246 nm, corresponding to the (111) plane) inside the nanowire, presenting a core–shell structure. The bright spots correspond to the Ir clusters, which were composed of several adjacent Ir atoms evenly dispersed in the lattice of CoO. The sub‐nano Ir clusters were uniformly covered on the surface of the core–shell structure, with an average diameter of 0.7 ± 0.2 nm.

As shown in Figure 2d, high‐coverage sub‐nano Ir clusters were embedded in the CoO lattice. The anchored Ir clusters could maximize the exposure of the active sites, improve atomic utilization efficiency, and enhance stability by bonding with Co atoms.^[^ ^25^ ^]^ Figure 2e presents the 3D model of the sub‐nano Ir cluster in region B of Figure 2d. Furthermore, the nanostructure of Ir cluster@CoO, Co_3_O_4_/CeO_2_, CoO/CeO_2_, and CeO_2_ were shown in Figures S3–S6 (Supporting Information). The line scanning profiles in Figure 2f revealed a broad distribution of Ir, Co, and O throughout the nanowire. In contrast, the signal of element Ce was observed only inside the nanowire, confirming the generation of a core–shell configuration. As illustrated in the atomic force microscope (AFM) image (Figure 2g; Figure S7, Supporting Information), the Ir cluster@CoO/CeO_2_ (Ra = 7.8 nm) exhibited a higher roughness than Ir^3+^@Co_3_O_4_/CeO_2_ (Ra = 1.6 nm), thereby confirming the morphological change and phase transition caused by in situ exsolution.^[^ ^26^ ^]^ In addition, the EDX elemental mapping images demonstrated that the Ir, Ce, Co, and O elements were uniformly distributed across the entire nanowire (Figure 2h–k). Notably, the actual content of Ir in Ir cluster@CoO/CeO_2_ was measured to be ≈2.23 wt.% by the inductively coupled plasma optical emission spectrometry (ICP‐OES).

**Figure 3:**
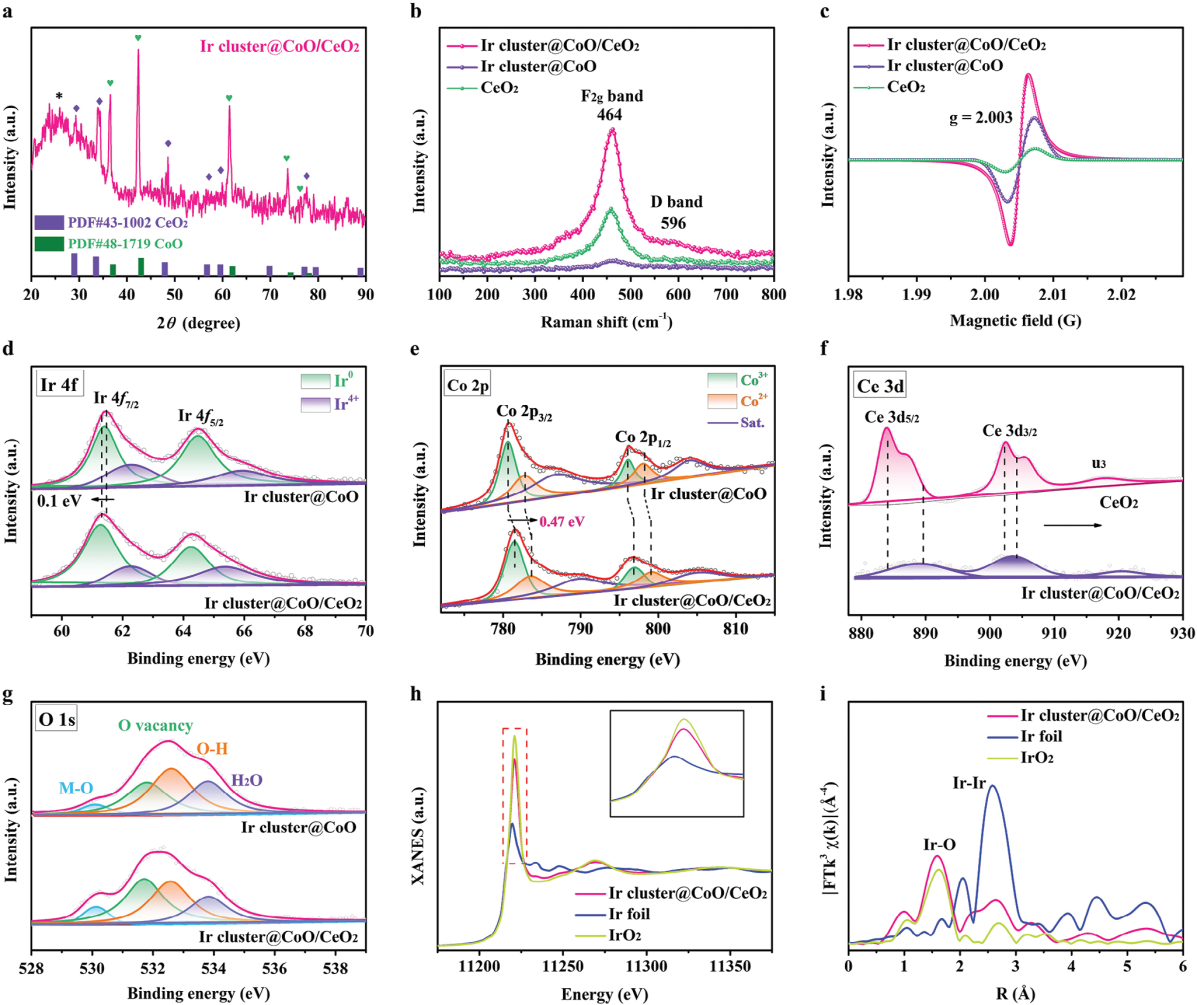
Structural characterization of the Ir cluster@CoO/CeO_2_. a) XRD pattern, b) Raman spectra, and c) EPR spectra of Ir cluster@CoO/CeO_2_, Ir cluster@CoO, and CeO_2_, respectively. XPS spectra of d) Ir 4f, e) Co 2p, f) Ce 3d, and g) O 1s for different catalysts. h) Ir L_3_‐edge XANES spectra and i) FT‐EXAFS curves of the Ir L_3_‐edge for the Ir cluster@CoO/CeO_2_, Ir foil, and IrO_2_.

The composition analysis of Ir cluster@CoO/CeO_2_ was performed using X‐ray diffraction (XRD). As displayed in **Figure** **3**a, the characteristic peaks could be indexed to CeO_2_ (JCPDS 43–1002) and CoO (JCPDS 48–1719). Meanwhile, no metallic Ir phase was detected, indicating the absence of Ir nanoparticles. The XRD patterns of Co_3_O_4_/CeO_2_, Ir^3+^@Co_3_O_4_/CeO_2_, and CeO_2_ were shown in Figure S8 (Supporting Information). From the XRD results, a phase transition occurred for the Co species after the hydrogenation process, resulting in the reduction of Ir^3+^@Co_3_O_4_/CeO_2_ to Ir cluster@CoO/CeO_2_.^[^ ^27^ ^]^ The Ir clusters embedded on the surface prevent further reduction of cobalt oxide to the lower valence Co species in the reducing atmosphere, thereby enhancing the insoluble of the catalyst across various pH environments, particularly in acidic conditions.^[^ ^28^ ^]^ Conversely, at elevated reduction temperatures, CoO undergoes further reduction to Co^0^ in the absence of Ir, which leads to a reduction in the stability of the catalyst across different pH conditions (Figure S9, Supporting Information). The broad peak represented by an asterisk at ≈26° was the typical peak of C (002).^[^ ^29^ ^]^ Further, the two peaks lying ≈646 and 596 cm^−1^ in the Raman spectra (Figure 3b) could be assigned to fluorite F_2g_ symmetry mode (F_2g_ band) and defect‐induced mode (D band) of CeO_2_, respectively.^[^ ^30^ ^]^ From the electron paramagnetic resonance (EPR) spectra in Figure 3c, the signal at g = 2.003 represented oxygen vacancies. The peak intensity of the Ir cluster@CoO/CeO_2_ sample was significantly higher than that of the Ir cluster@CoO and CeO_2_, indicating a greater number of oxygen vacancies.^[^ ^31^ ^]^ The increase in oxygen vacancy was caused by the spontaneous segregation (in situ exsolution) in bimetallic oxides.

Furthermore, the XPS was used to analyze the surface composition and elemental valence states of the catalyst. The XPS survey spectrum in Figure S10 (Supporting Information) revealed the presence of Ir, Ce, Co, and O elements in the different catalysts. As expected, for Ir cluster@CoO/CeO_2_, the signal of the Ce element became relatively poor due to the limited depth of the XPS probe detection signal, further confirming the formation of the core–shell structure. For Ir 4f spectra in Figure 3d, Ir cluster@CoO/CeO_2_ had slightly lower binding energies (≈0.1 eV) compared to Ir cluster@CoO, suggesting that Ir obtained part electrons.^[^ ^32^ ^]^ The surface electron‐enriched Ir endowed the catalyst's beneficial electronic microenvironment to enhance catalytic activity and stability. Figure 3e and Figure S11 (Supporting Information) illustrated the Co 2p XPS spectra of the Ir cluster@CoO/CeO_2_, Ir cluster@CoO and CoO/CeO_2_, where the spectra made up of four main peaks centered at 780.9, 784.2, 796.1, and 797.2 eV, corresponding to Co^3+^ (2p_3/2_), Co^2+^ (2p_3/2_), Co^3+^ (2p_1/2_), and Co^2+^ (2p_1/2_), respectively. The two peaks located at 787.1 and 803.3 eV were satellite peaks.^[^ ^33^ ^]^ In addition, the binding energy of Co 2p in the Ir cluster@CoO/CeO_2_ was positively shifted ≈0.47 eV compared with Ir cluster@CoO, implying that the Co lost electrons and the introduction of CeO_2_ could promote electronic redistribution.^[^ ^34^ ^]^ In addition, the Co^2+^ over Co^3+^ ratio is decreased after coupling with CeO_2_. The ratios of Co^2+^: Co^3+^ in Ir cluster@CoO and Ir cluster@CoO/CeO_2_ are 0.92: 1 and 0.86: 1, respectively, suggesting an increased valence state of Co on the surface of Ir cluster@CoO/CeO_2_ (Table S1, Supporting Information). Therefore, the shift in peak position and the changing trend of the Co^2+^: Co^3+^ ratio jointly confirm that Co species lose electrons and their valence increases for the Ir cluster@CoO/CeO_2_ catalyst. In comparison to CeO_2_, the obvious positive shift of Ce 3d paeks in Ir cluster@CoO/CeO_2_ unveiled the strong electronic interactions among Ce and Co atoms (Figure 3f).^[^ ^35^ ^]^ In Figure 3g, the binding energies of O 1s could be deconvoluted into four peaks corresponding to metal‐oxygen bonds (M─O at 530.1 eV), oxygen vacancy (O_2_^2−^/O^−^ at 531.7 eV), the oxygen of surface hydroxyl groups (O─H at 532.5 eV), and water molecules (H_2_O at 533.8 eV).^[^ ^36^ ^]^ The nitrogen adsorption‐desorption technique examined the surface area and pore structure of the catalysts (Figure S12, Supporting Information). The analysis results demonstrated that the Ir cluster@CoO/CeO_2_ possessed a specific surface area of 27.2 m^2^ g^−1^, higher than that of Ir cluster@CoO (10.7 m^2^ g^−1^) and CeO_2_ (9.1 m^2^ g^−1^). The pore size distribution curves (inset Figure S12, Supporting Information) manifested that the Ir cluster@CoO/CeO_2_ had a hierarchically porous structure, which could contribute to the gas diffusion and mass/charge transfer during the reaction. The results of Raman, EPR, and XPS were consistent with that of XRD, confirming the occurrence of phase transition under the reducing atmosphere. After reduction, Co ions were released from the bimetallic oxide, creating cation defects and oxygen vacancies. Simultaneously, Ir cations gained electrons and were reduced to Ir nanoclusters on the surface, accompanied by a structural transformation to a core–shell configuration. Additionally, the unobstructed electronic pathway avoided the aggregation of Ir species during the reaction process and mitigated the sensitivity of the catalyst to the environment.

The X‐ray absorption spectroscopy (XAS) was then conducted to investigate the local electronic structure of the Ir species. Except for the peak positions and relative intensities, the X‐ray absorption near‐edge structure (XANES) spectra for the Ir L_3_‐edge in Figure 3h display analogous structural features. The prominent peak of the Ir L_3_‐edge (historically called the white line) corresponds to the electron transition from the occupied 2p_3/2_ orbital to the partially occupied Ir 5d orbitals. Furthermore, the magnitude of its intensity is directly proportional to the density of unoccupied 5d orbitals.^[^ ^37^, ^38^ ^]^ The white line intensity of the Ir L_3_‐edge for Ir cluster@CoO/CeO_2_ is situated between metallic Ir and IrO_2_, disclosing that the number of unoccupied states in the 5d band for Ir cluster@CoO/CeO_2_ is between them, which has been utilized to correlate the catalytic performance of noble metal‐based electrocatalysts to changes in their local electronic states.^[^ ^39^, ^40^ ^]^ Figure 3i presents the Fourier transforms of the extended X‐ray absorption fine structure (EXAFS) spectra, which are utilized to investigate the local environment surrounding Ir. Notably, peaks observed at 2.48 Å, are indicative of the Ir‐Ir interaction, which is evident in both the Ir cluster@CoO/CeO_2_ and metallic Ir. In Ir cluster@CoO/CeO_2_, the prominent peak observed at 1.75 Å indicates the scattering interaction associated with the Ir─O bond.^[^ ^41^, ^42^ ^]^ Furthermore, the presence of the Ir─Ir bond substantiates the assertion that the Ir species within the Ir cluster@CoO/CeO_2_ catalyst are present in the form of Ir clusters.

**Figure 4:**
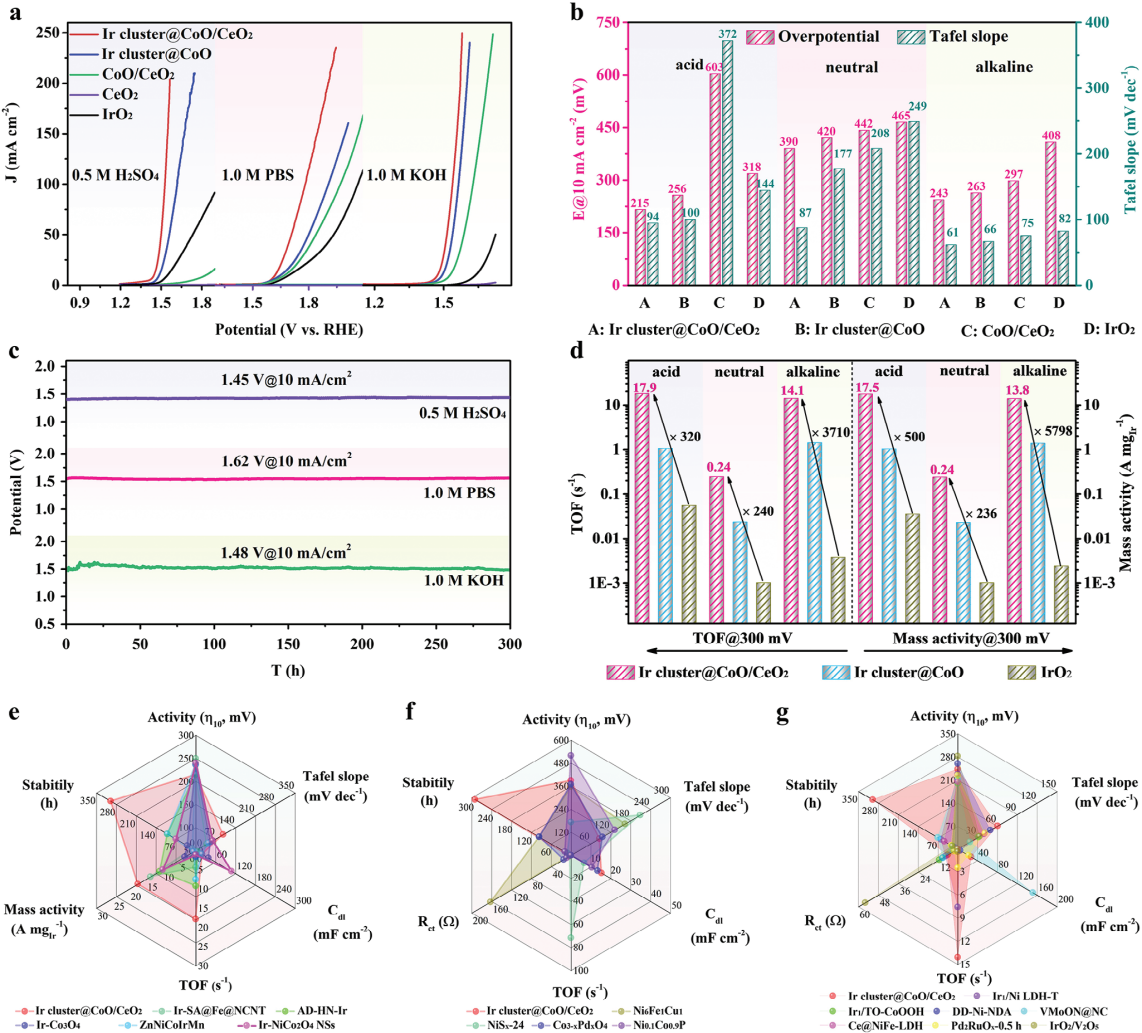
OER performance in different electrolytes. a) LSV curves. b) Comparison of overpotential and Tafel slope values. c) Chronopotentiometric measurement of Ir cluster@CoO/CeO_2_ for 300 h at 10 mA cm^−2^. d) Comparison of turnover frequency (TOF) and mass activity (MA) at an overpotential of 300 mV. e) Comparison of performance‐determining factors between Ir cluster@CoO/CeO_2_ and other reported catalysts in acidic medium. The variable values shown were η_10_, Tafel slope, C_dl_, TOF, MA, and stability, the detailed information is given in Table S2 (Supporting Information). f,g) Comparison of performance‐determining factors between Ir cluster@CoO/CeO_2_ and other reported catalysts in neutral and alkaline medium, respectively. The variable values shown were η_10_, Tafel slope, C_dl_, TOF, R_ct_, and stability, the detailed information was given in Tables S3 and S4 (Supporting Information).

**Figure 5:**
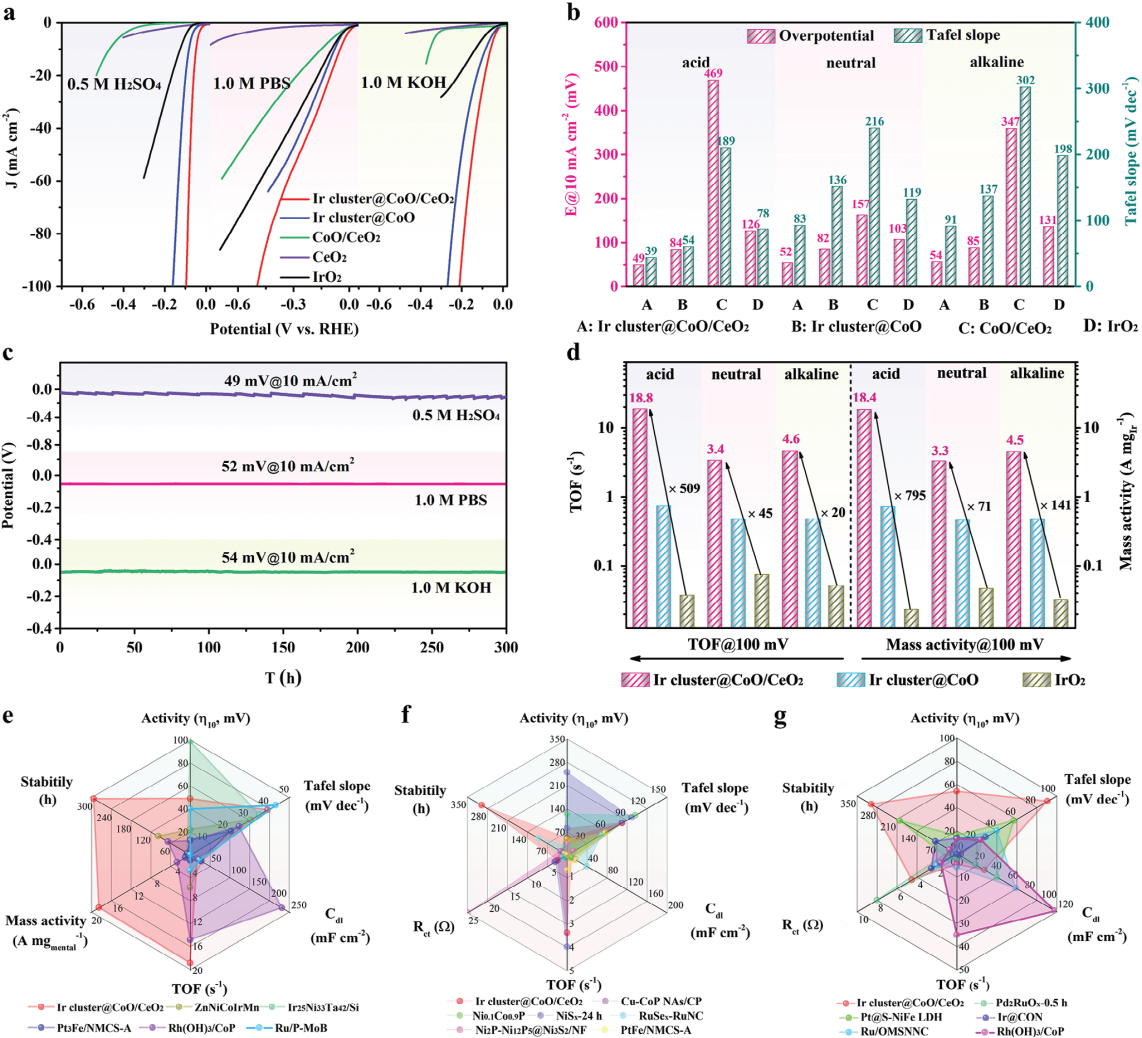
HER performance in different electrolytes. a) LSV curves. b) Comparison of overpotential and Tafel slope values. c) Chronopotentiometric measurement of Ir cluster@CoO/CeO_2_ for 300 h at 10 mA cm^−2^. d) Comparison of TOF values and MA at an overpotential of 100 mV. e) Comparison of performance‐determining factors between Ir cluster@CoO/CeO_2_ and other reported catalysts in acidic medium. The variable values shown were η_10_, Tafel slope, C_dl_, TOF, MA, and stability, the detailed information was given in Table S5 (Supporting Information). f,g) Comparison of performance‐determining factors between Ir cluster@CoO/CeO_2_ and other reported catalysts in neutral and alkaline medium, respectively. The variable values shown were η_10_, Tafel slope, C_dl_, TOF, R_ct_, and stability, the detailed information was given in Tables S6 and S7 (Supporting Information).

The electrocatalytic OER performances were measured in a typical three‐electrode system under different pH conditions. The linear scanning voltammetry (LSV) curves in **Figure** **4**a suggested that the Ir cluster@CoO/CeO_2_ possessed excellent OER activities at wide pH ranges due to the high activity of Ir clusters and the abundant interface sites. Notably, the Ir cluster@CoO/CeO_2_ responded well to high currents in different pH environments (Figure S13, Supporting Information), indicating its enormous potential in industrial applications. The corresponding Tafel slope of these samples was calculated from the LSV curves and displayed in Figure S14 (Supporting Information). The overpotentials and Tafel slope values of the catalysts at 10 mA cm^−2^ were summarized in Figure 4b. Specifically, the Ir cluster@CoO/CeO_2_ could drive a current density of 10 mA cm^−2^ at ultralow overpotentials of 215, 390, 243 mV for OER and 94, 87, 61 mV dec^−1^ for Tafel slope in acid, neutral, and alkaline electrolyte, respectively. Compared with Ir cluster@CoO, CoO/CeO_2_, CeO_2_, and IrO_2_, Ir cluster@CoO/CeO_2_ had the lowest overpotential and Tafel slope value at different pH conditions, illustrating the superior reaction rates of Ir cluster@CoO/CeO_2_ toward OER.^[^ ^43^ ^]^ The effect of metal precursor (iridium chloride hydrate) content on OER activity has also been studied. We depicted the relation of metal precursor content as the function of the overpotentials for the electrode. The OER activities of the catalyst were the best when the amount of metal precursor was 6 mg, as revealed by the OER LSV curves (Figure S15, Supporting Information). Further, to explore the high intrinsic activity of Ir cluster@CoO/CeO_2_ for OER, the double‐layer capacitance (C_dl_) was tested by CV measurements to calculate the electrochemical surface area (ECSA) (Figures S16–S18, Supporting Information).^[^ ^44^ ^]^ The Ir cluster@CoO/CeO_2_ illustrated the highest C_dl_ of 19.6, 16.1, and 28.1 mF cm^−2^ in 0.5 m H_2_SO_4_, 1.0 m PBS, and 1.0 m KOH respectively, which were larger than the experimental values of other comparison samples. The electrochemical impedance spectroscopy (EIS) Nyquist curves in Figure S19 (Supporting Information) indicated that the Ir cluster@CoO/CeO_2_ had the fastest charge transfer in pH universal environments.^[^ ^45^ ^]^ To further determine whether the enhanced OER activity is solely contingent on the increased active sites, the ECSA‐normalized OER polarization curves are calculated. From the ECSA‐normalized LSV curves (Figure S20, Supporting Information), the Ir cluster@CoO/CeO_2_ catalyst still exhibits the highest activity, suggesting intrinsically improved OER activity on the Ir cluster@CoO/CeO_2_. As shown in Figure 4c, the Ir cluster@CoO/CeO_2_ catalyst could operate stably in acidic, neutral, and alkaline electrolytes for at least 300 h, respectively. Small overpotential attenuation could be observed on the electrode, proving that the Ir cluster@CoO/CeO_2_ could achieve steady OER operation. In addition, the catalyst also exhibits good durability for OER under high current conditions of 100 and 400 mA cm^−2^ in different pH electrolytes (Figure S21, Supporting Information). Based on the above experimental results, Ir cluster@CoO/CeO_2_ was proven to be a highly active and robust OER electrocatalyst under full‐pH conditions. As an important parameter for evaluating the instantaneous efficiency of catalysts, the TOF value represented the number of molecules reacting at each active site per unit time, reflecting the intrinsic activity of the catalyst. As displayed in Figure 4d, Ir cluster@CoO/CeO_2_ reached the TOF values of 17.90, 0.24, and 14.10 s^−1^ at overpotential of 300 mV in 0.5 m H_2_SO_4_, 1.0 m PBS, and 1.0 m KOH, respectively, which were quite larger than those of Ir cluster@CoO (1.04, 0.02, and 1.40 s^−1^) and IrO_2_ (0.056, 0.001, and 0.0038 s^−1^), elucidating that Ir cluster@CoO/CeO_2_ delivered a high instantaneous efficiency for OER catalysis.^[^ ^46^ ^]^ In particular, the TOF values of Ir cluster@CoO/CeO_2_ were 2–3 orders of magnitude higher than commercial IrO_2_. Moreover, at an overpotential of 300 mV, the Ir cluster@CoO/CeO_2_ also had mass activities (MA) of 17.50, 0.24, and 13.80 A mg_Ir_^−1^ at acid, neutral, and alkaline solution, which were orders of magnitude higher than Ir cluster@CoO and IrO_2_. The TOF‐normalized and mass‐normalized LSV curves for OER in different media were displayed in Figures S22 and S23 (Supporting Information). Furthermore, the stability and durability of core–shell Ir cluster@CoO/CeO_2_ electrodes were tested by CV cycling and chronoamperometry. LSV curves of Ir cluster@CoO/CeO_2_ for OER declined negligibly after 5000 CV cycles in different pH solutions (Figure S24, Supporting Information). Specifically, for acidic OER, in the absence of Ir clusters anchored on the catalyst surface, the activity of CoO/CeO_2_ decayed rapidly, indicating that the surface Ir cluster layer made a significant contribution to the stability of the catalyst (Figure S25, Supporting Information).^[^ ^47^ ^]^ Charge compensation from valence‐variable Ce atoms to Co and Ir atoms can extremely avoid excessive loss of lattice oxygen and Ir leaching, greatly enhancing the stability of the catalyst.^[^ ^48^ ^]^ The radar plots showed that the comprehensive electrocatalytic OER performance of Ir cluster@CoO/CeO_2_ was significantly better than other advanced catalysts within the full‐pH range (Figure 4e–g). Subsequently, the pH‐universal HER performance of Ir cluster@CoO/CeO_2_ was evaluated. As shown in **Figure** **5**a, the overpotentials of Ir cluster@CoO/CeO_2_ were 49, 52, and 54 mV when the current density reached 10 mA cm^−2^ in acidic, neutral, and alkaline electrolytes. These values were much lower than those of Ir cluster@CoO (84, 82, and 85 mV), CoO/CeO_2_ (469, 157, and 347 mV), and IrO_2_ (126, 103, and 131 mV), unveiling the enhanced HER activities. Notably, the Ir cluster@CoO/CeO_2_ still possessed small overpotentials at high current densities within a broad pH range (Figure S26, Supporting Information), indicating its great potential for application in clean hydrogen energy. As expected, a volcano‐type relation was observed between iridium metal precursor content and the overpotentials for the pH universal HER. At a dosage of 6 mg for the Ir precursor, the catalyst exhibits the highest HER catalytic activities (Figure S27, Supporting Information). The Tafel slopes of Ir cluster@CoO/CeO_2_ were determined to be 39, 83, and 91 mV dec^−1^, significantly smaller than these of Ir cluster@CoO (54, 136, and 137 mV dec^−1^), CoO/CeO_2_ (189, 216, and 302 mV dec^−1^), and IrO_2_ (78, 119, and 198 mV dec^−1^) (Figure 5b; Figure S28, Supporting Information), suggesting the fast HER kinetics.^[^ ^49^ ^]^ Meanwhile, Ir cluster@CoO/CeO_2_ also had a higher ECSA as compared with other catalysts evidenced by a larger C_dl_ as summarized in Figures S29–S31 (Supporting Information). The ECSA‐normalized LSV curves for HER in different electrolyte solutions show that Ir cluster@CoO/CeO_2_ catalyst has the highest intrinsic activity (Figure S32, Supporting Information). Additionally, as illustrated in Figure S33 (Supporting Information), the noticeably smaller R_ct_ values suggested that the catalyst had better conductivity, which was conducive to improving the HER activity on Ir cluster@CoO/CeO_2_. Then, poor stability for HER was also found in 0.5 m H_2_SO_4_ for the absence of Ir clusters (Figure S34, Supporting Information). The results revealed that the protective and synergistic effects of high coverage sub‐nano Ir clusters cannot be ignored during the HER process. The LSV curves after 5000 CV in different electrolytes had small attenuation, revealing the HER activities remained good after the accelerated stability test (Figure S35, Supporting Information). Additionally, to investigate the long‐term electrochemical stability of HER in different electrolytes, the chronopotentiometry test was performed. The actual time‐potential response signal remained nearly unchanged after a continuous running of 300 h, even exceeding 50 h at high current densities (at 100 and 400 mA cm^−2^), which showed the high durability of Ir cluster@CoO/CeO_2_ for HER in a wide pH range (Figure 5c; Figure S36, Supporting Information). From the TOF‐/MA‐normalized polarization curves of HER in Figures S37 and S38 (Supporting Information), the as‐prepared catalyst had significantly improved TOF and MA values in a wide pH range. Concretely, at the overpotential of 100 mV in different pH conditions, the TOF values (18.8, 3.4, and 4.6 H_2_ s^−1^) of Ir cluster@CoO/CeO_2_ were much greater than those of Ir cluster@CoO and IrO_2_, respectively, attesting its enhanced intrinsic electrocatalytic efficiency (Figure 5d). As expected, Ir cluster@CoO/CeO_2_ also generated the highest MA of 18.4, 3.3, and 4.5 A mg_Ir_^−1^ at η_100_, which were significantly larger than those of Ir cluster@CoO and IrO_2_. Moreover, the systematic comparison of η_10_, Tafel slope, C_dl_, TOF, MA, and stability at 10 mA cm^−2^ was displayed in Figure 5e. The comprehensive electrocatalytic HER performance of Ir cluster@CoO/CeO_2_ prominently surpassed that of other state‐of‐the‐art catalysts. In addition, the comparison of various performance indicators of the catalyst showed that compared to other previously reported electrocatalysts, Ir cluster@CoO/CeO_2_ also had significant advantages in neutral and alkaline electrolytes (Figure 5f,g).

**Figure 6:**
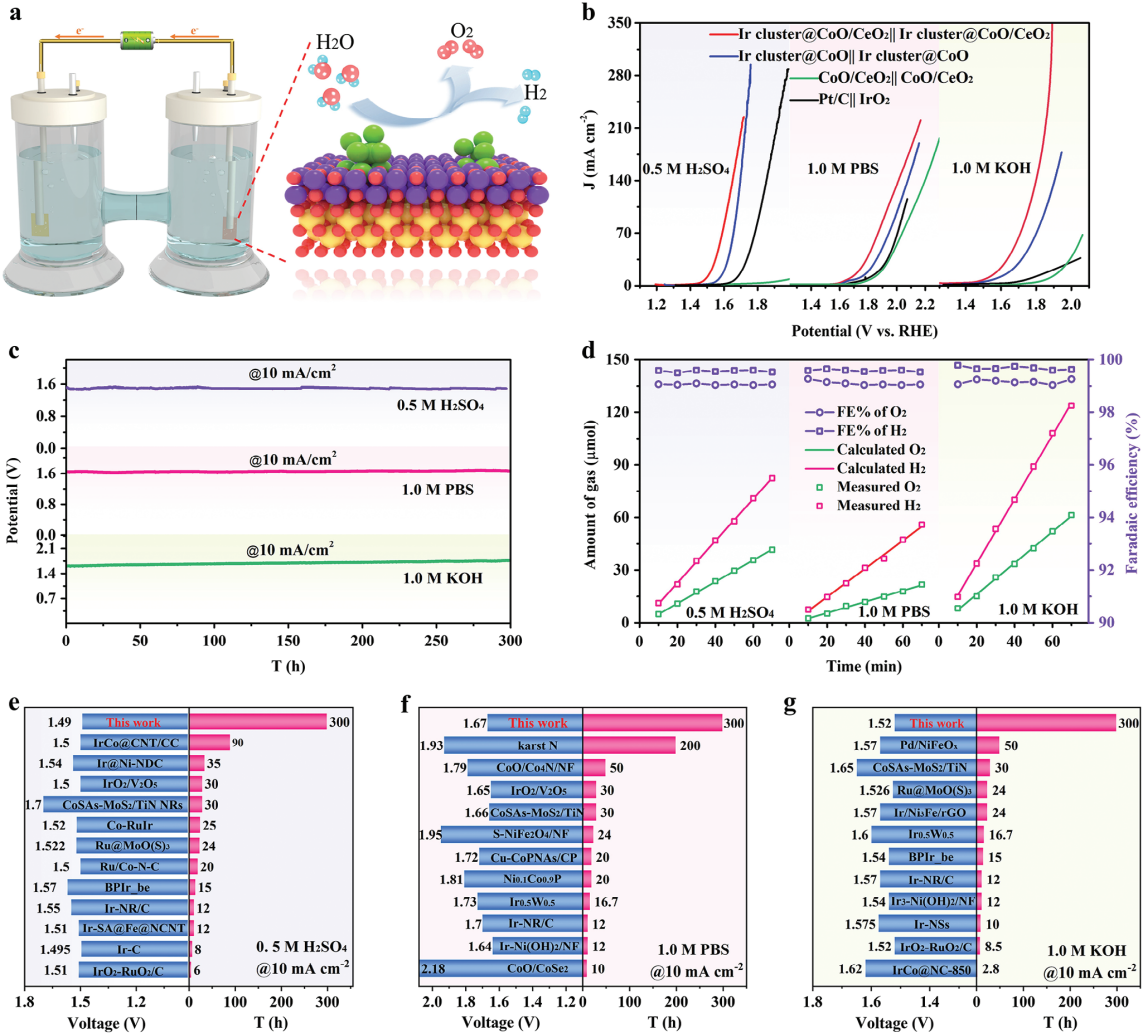
Electrocatalytic overall water splitting performance in different electrolytes. a) Schematic diagram of a two‐electrode water electrolysis device. b) Polarization curves of the Ir cluster@CoO/CeO_2_||Ir cluster@CoO/CeO_2_ and other control samples electrolyzers. c) Chronopotentiometry response for 300 h of Ir cluster@CoO/CeO_2_||Ir cluster@CoO/CeO_2_ under constant current conditions of 10 mA cm^−2^. d) Generated H_2_ and O_2_ amounts and the calculated corresponding Faraday efficiency for the assembled electrolyzer at 10 mA cm^−2^. e–g) Comparison of activity and stability between Ir cluster@CoO/CeO_2_ and reported electrocatalysts in 0.5 m H_2_SO_4_, 1.0 m PBS, and 1.0 m KOH, respectively. The detailed information is given in Tables S8–S11 (Supporting Information).

The capability of Ir cluster@CoO/CeO_2_ serving as both cathode and anode simultaneously in different electrolytes was subsequently investigated. The overall water‐splitting reaction was carried out in an H‐type two‐electrode electrolytic cell, as exhibited in **Figure** **6**a. The Ir cluster@CoO/CeO_2_||Ir cluster@CoO/CeO_2_ electrolyzer could drive water electrolysis with a cell voltage of 1.49, 1.67, and 1.52 V at 10 mA cm^−2^ in the acidic, neutral, and alkaline environment, respectively (Figure 6b), which were superior to other electrolyzers and even commercial Pt/C||IrO_2_. Moreover, it also showed prominent durability in the chronopotentiometry test, which could operate at least 300 h at 10 mA cm^−2^ (Figure 6c). In addition, the Faradic efficiency for generated H_2_ and O_2_ of Ir cluster@CoO/CeO_2_ were evaluated using a lab‐scale drainage and gas collection apparatus setup. From Figure 6d, the volume ratio of H_2_ (generated by the cathode) to O_2_ (generated by the anode) in three different electrolytes conformed to the theoretical value (2:1), proving the Faradic efficiency of Ir cluster@CoO/CeO_2_ in water electrolysis was close to 100%. When compared to the activity and stability of other iridium‐ or cobalt‐based catalysts, Ir cluster@CoO/CeO_2_ had a significant advantage at the entire pH range for the overall water splitting (Figure 6e–g; Tables S8–S11, Supporting Information). The SEM, TEM, XPS, and AC‐STEM characterization of the Ir cluster@CoO/CeO_2_ after water splitting were shown in Figures S39–S41 (Supporting Information), attesting that the catalyst had excellent structural stability. In addition, we investigated the prospective utilization of the Ir cluster@CoO/CeO_2_ catalyst under industrial conditions (80 °C, 30% KOH). As illustrated in Figure S42 (Supporting Information), the Ir cluster@CoO/CeO_2_ catalyst shines with impressive OER, HER, and overall water‐splitting performance even in industrial settings.

**Figure 7:**
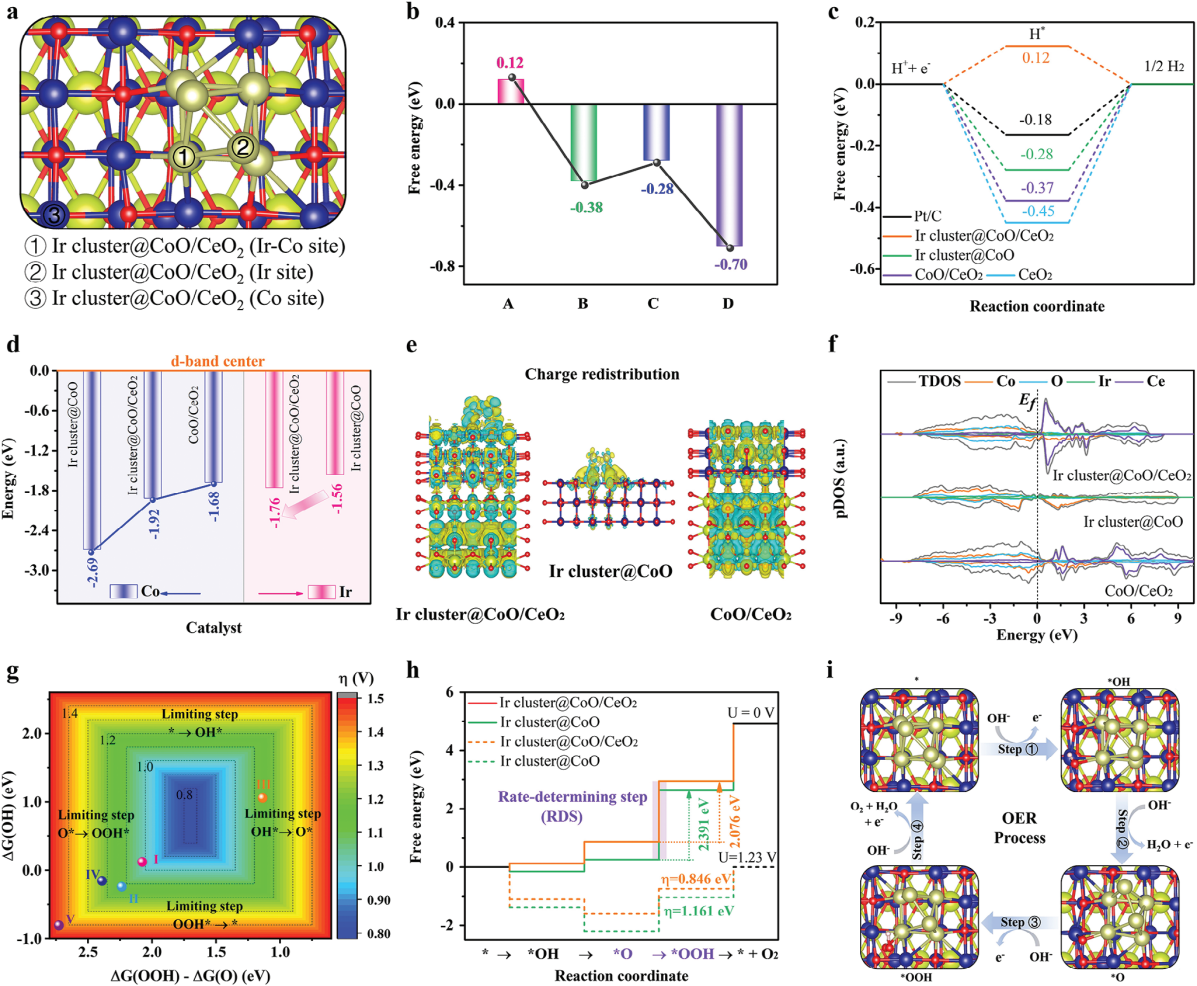
DFT mechanism exploration. a) Models of different adsorption sites on Ir cluster@CoO/CeO_2_. The blue, yellow, golden, and red ball marks Co, Ce, Ir, and O atoms, respectively. b) The corresponding H* free‐energy diagrams on A, B, C, and D. A: Ir cluster@CoO/CeO_2_ (Ir site), B: Ir cluster@CoO/CeO_2_ (Co site), C: Ir cluster@CoO (Ir site), and D: Ir cluster@CoO (Co site). c) The predicted free‐energy diagrams for HER at U = 0 V. d) Comparison of the Ir and Co d‐band center values of Ir cluster@CoO/CeO_2_, Ir cluster@CoO, and CoO/CeO. e) Charge density difference. The yellow (blue) shadows represent the electron accumulation (depletion). f) PDOS of different catalysts. g) Contour plot of theoretical overpotential as a function of ΔG_O*_–ΔG_OH*_ and ΔG_OOH*_. The corresponding overpotentials (mV) are shown in the right bar. Constructed by assuming a scaling relation ΔG_OOH*_ = ΔG_OH*_ + 3.2 eV. h) Standard free energy diagrams of the OER pathway on Ir cluster@CoO/CeO_2_ and Ir cluster@CoO at U = 0 and 1.23 V. i) The four electrons mechanism for Ir cluster@CoO/CeO_2_ of OER.

Density functional theory calculation (DFT) was further studied to explore the electronic structures and reaction mechanisms of the catalysts. The simulation models of Ir cluster@CoO/CeO_2_, Ir cluster@CoO, CoO/CeO_2_, and CeO_2_ constructed based on the experiment results are shown in Figure S43 (Supporting Information). For Ir cluster@CoO/CeO_2_, all possible active sites are shown in **Figure** **7**a. Through the calculation of Gibbs free energy of H* adsorption, it was determined that the HER reaction site was on the Ir site rather than on the Co site of the Ir─Co bond (Figure 7b; Figure S44, Supporting Information). The results confirmed the ΔG_H*_ value of Ir cluster@CoO/CeO_2_ was closer to 0 eV (the optimal criteria for HER) than the other considered catalysts.^[^ ^50^ ^]^ As shown in Figure 7c, compared with Ir cluster@CoO (ΔG_H*_ = −0.28 eV), CoO/CeO_2_ (ΔG_H*_ = −0.37 eV), and CeO_2_ (ΔG_H*_ = −0.45 eV), the Ir cluster@CoO/CeO_2_ possessed the optimum Gibbs free energy of H* adsorption (ΔG_H*_ = 0.12 eV), even better than commercial Pt/C (ΔG_H*_ = −0.18 eV). The differential charge density further demonstrated the significant influence of molecular structure on electronic configuration. From the side and top views of the models, Ir cluster@CoO/CeO_2_ experienced noticeable electron transfer and redistribution compared to Ir cluster@CoO and CoO/CeO_2_ (Figure 7e; Figure S45, Supporting Information). More charge accumulation (yellow shadows) was found around the Ir‐Co bond accompanied by charge depletion (blue shadows) around the Co and Ce atoms. The Bader charge analysis in Table S12 (Supporting Information) revealed that the Ir atoms in the Ir cluster@CoO/CeO_2_ exhibited a lower charge depletion (−0.10 e) than that of the Ir cluster@CoO (−0.07 e). In contrast, the Co charge at the interface was higher than that of Ir cluster@CoO, indicating the electrons transferred from Co to Ir. The Ce charge at the Ir cluster@CoO/CeO_2_ interface was higher than that of CoO/CeO_2_, which means the Ce loses electrons. The result suggested the electrons were transferred from Ce to Ir, consistent with the XPS results.^[^ ^51^ ^]^ The surface Ir clusters optimize the electronic structure of the catalyst by disrupting the original charge distribution, reshaping a new charge balance, and generating local charge aggregation, thereby improving its electrocatalytic activity.^[^ ^24^ ^]^ The analysis of the d‐band center (ɛ_d_) of Co and Ir atoms at the surface was displayed in Figure 7d and Figure S46 (Supporting Information). Compared with Ir cluster@CoO, the ɛ_d_ of the Ir atom in Ir cluster@CoO/CeO_2_ underwent a negative shift (−1.56 → −1.76 eV) far away from the Fermi energy level. Meanwhile, the ɛ_d_ of the Co atom in Ir cluster@CoO/CeO_2_ possessed a moderate bandgap of −1.92 eV compared with Ir cluster@CoO (−2.69 eV) and CoO/CeO_2_ (−1.68 eV). Following the classical d‐band theory, different sites in catalysts had different interactions with oxygen‐containing intermediates. The results indicated that moderate Co atom adsorption and weakened Ir atom adsorption collaborative optimized the adsorption path in Ir cluster@CoO/CeO_2_, proving the positive effect of the Ir‐Co site on reducing the energy barrier during the OER.^[^ ^52^ ^]^ Furthermore, the projected density of states (pDOS) curves of various catalysts are depicted in Figure 7f. The total DOSs for the Ir cluster@CoO/CeO_2_ near the Fermi level are more closely aligned and continuous, suggesting that the sub‐nano Ir cluster assembled with the core–shell CoO/CeO_2_ maintains superior conductivity and unobstructed electron transfer.^[^ ^53^ ^]^ Theoretical overpotential volcano plots for all possible active sites were shown in the volcano plot in Figure 7g, in which a two‐dimension energy contour map of OER theoretical overpotentials regarding different sites in catalysts (I: Ir‐Co site, II: Ir site, and III: Co site at Ir cluster@CoO/CeO_2_; IV: Ir‐Co site at Ir cluster@CoO; V: CeO_2_). The Ir cluster@CoO/CeO_2_ was predicted to have lower theoretical overpotentials compared to Ir cluster@CoO and CoO/CeO_2_, which was consistent well with experimental trends.^[^ ^54^ ^]^ Figure S47a (Supporting Information) illustrated the Gibbs free energy profiles (ΔG) for the intermediates and products on Ir─Co, Ir, and Co sites of Ir cluster@CoO/CeO_2_ during the alkaline OER processes at U = 0 V. For Ir─Co and Ir sites, the rate‐determining step (RDS) is *O→*OOH. Whereas, the RDS is *OH→*O for the Co sites. The Ir─Co site owned the lowest overpotential under the same OER path, compared to the Ir site, and also excelled over the Co site. Figure S47b (Supporting Information) displays the Gibbs adsorption‐free energy of different oxygen‐containing intermediates (*OH, *O, and *OOH) at different active sites. The Gibbs free energy calculation results show that in the Ir cluster@CoO/CeO_2_ catalyst, the most favorable active site is the Ir─Co dual site, which has the optimal reaction intermediate free energy. Thus, the adsorption/desorption of oxygen‐containing intermediates was more easily carried out on the electroactive Ir─Co sites. The corresponding adsorption models during OER of Ir cluster@CoO/CeO_2_ and Ir cluster@CoO were displayed in Figures S48–S51 (Supporting Information). Figure 7h illustrates the ΔG for the intermediates and products on Ir cluster@CoO/CeO_2_ and Ir cluster@CoO during the alkaline OER processes at U = 0 and U = 1.23 V versus RHE. Apparently, the RDS was *O → *OOH according to their distinct adsorption capability of O species. The ΔG_RDS_ was found to be the lowest when mediated by Ir cluster@CoO/CeO_2_. The results indicated that the Ir cluster@CoO/CeO_2_ interface (Ir─Co site) is the electroactive site that substantially boosts OER kinetics.^[^ ^55^ ^]^ Additionally, the detailed four‐electron mechanism for the Ir cluster@CoO/CeO_2_ catalyst in the OER process is illustrated in Figure 7i.

## 3 Conclusion

In summary, we have constructed high coverage Ir clusters on core–shell CoO/CeO_2_ catalyst as an efficient full‐pH bifunctional electrocatalyst. Impressively, the as‐prepared Ir cluster@CoO/CeO_2_ possessed a superior full‐pH overall water‐splitting performance and fast kinetics. The sub‐nano Ir clusters (≈0.7 nm) confined and embedded on the lattice of the CoO shell endow a distinctive interface configuration and appropriate electronic structure. The core–shell CoO/CeO_2_ heterogeneous has opened up an unobstructed and sturdy transmission channel for electronic and mass transmission. These structural and electronic advantages give this catalyst better activity and stability than commercial precious metals noble catalysts in full‐pH electrolytes. DFT reveals that the Ir cluster@CoO/CeO_2_ interface with electronic reconstruction affords near‐optimal HER/OER adsorption energy and augmented resistance to dissolution. This work provides a new idea for constructing sub‐nano cluster/heterostructure interface electrocatalysts not only for HER but also for OER and overall water splitting.

## Conflict of Interest

The authors declare no conflict of interest.

## Supporting information



Supporting Information

## Data Availability

The data that support the findings of this study are available from the corresponding author upon reasonable request.
