# Supplementary material for: High Coverage Sub‐Nano Iridium Cluster on Core–Shell Cobalt‐Cerium Bimetallic Oxide for Highly Efficient Full‐pH Water Splitting
Source: Adv Sci (Weinh). 2024 Oct 14;11(45):2407475. doi: 10.1002/advs.202407475 (PMC11615758; doi:10.1002/advs.202407475)
Supplement: Supplementary file 1 — Supporting Information [file ADVS-11-2407475-s001.docx]

**Supporting Information**

High Coverage Sub-Nano Iridium Clusters Anchored on CoO/CeO_2_ Core-Shell Heterostructure for Efficient Overall Water Splitting Under pH-Universal Conditions

*Lili Zhang, Yuanting Lei, Yinze Yang, Dan Wang, Yafei Zhao, Xu Xiang, Huishan Shang*, Bing Zhang*

L. L. Zhang, Y. T. Lei, Dr. Y. Z. Yang, Dr. D. Wang, Dr. Y. F. Zhao, Dr. H. S. Shang, Dr. B. Zhang

School of Chemical Engineering, Zhengzhou University

Zhengzhou, 450001, China

E-mail: shanghs@zzu.edu.cn

Dr. X. Xu

State Key Laboratory of Chemical Resource Engineering, Beijing University of Chemical Technology

Beijing, 100029, PR China

**Section1.** **Experimental detail**

**Materials**

Carbon cloth (CC, type: WOS1009, thickness: 0.33 mm) was obtained from Taiwan CeTech Co., Ltd. All other chemical reagents were received from Aladdin Ltd (China) and utilized as received.

**Preparation of Co_3_O_4_/CeO_2_, Co_3_O_4_, and CeO_2_**

The Co(OH)F/CeF_3_ precursor nanowires were obtained by the previous experimental method. Under stirring conditions, Co(NO_3_)_2_·6H_2_O (1.6 mmol), Ce(NO_3_)_3_·6H_2_O (1.0 mmol), NH_4_F (10.0 mmol), and urea (6.0 mmol) were uniformly mixed in 24 mL deionized water. Then, the pink mixture solution into a 50 mL Teflon-lined autoclave. Put a piece of clean CC (2 × 2 cm^2^) into the autoclave and maintain it in an oven for 6 h at 120 °C. Rinsed clean the obtained Co(OH)F/CeF_3_ precursor with distilled water and anhydrous ethanol, drying and reserving. At 500 °C, calcined Co(OH)F/CeF_3_ precursor in a tubular furnace for 2 h under air atmosphere to obtain Co_3_O_4_/CeO_2_. The pure Co_3_O_4_ and CeO_2_ were obtained by the same procedure without adding the Ce(NO_3_)_3_·6H_2_O and Co(NO_3_)_2_·6H_2_O, respectively. The loading mass of the Co_3_O_4_/CeO_2_ on carbon cloth is 0.27 mg/cm^3^.

**Preparation of Ir cluster@CoO@CeO_2_, Ir cluster@CoO, and CoO@CeO_2_**

Dissolved a certain amount of iridium (Ⅲ) chloride hydrate in 10 mL of deionized water. Then, added Co_3_O_4_/CeO_2_ into the above solution, and slowly stirred for 30 minutes for sufficient ion exchange. The Ir^3+^@Co_3_O_4_/CeO_2_ was obtained by vacuum drying. The Ir^3+^@Co_3_O_4_/CeO_2_ was reduced at 300 °C for 3 h under H_2_/Ar (10%) to generate Ir cluster@CoO@CeO_2_. The Ir cluster@CoO was obtained by the same procedure by adding the Co_3_O_4_. The CoO@CeO_2_ was obtained by directly reducing the Co_3_O_4_/CeO_2_. In addition, the heating rate during the reduction process is 2.5 ^o^C/min.

**Catalyst characterization**

X-ray diffraction (XRD, D8 Bruker AXS, scan range of 10-80°) test was performed to identify the crystallinity and phase of samples. Raman (Lab RAM HR Evolution spectrometer, excitation line of 532 nm) was analyzed to record the stretching and bond vibration modes. Electron paramagnetic resonance (EPR, Bruker E-500 spectrometer, 100 kHz) measurement was implemented to display lone pair electron content. Scanning electron microscopy (SEM, Ultra 55Zeiss) information was acquired to observe the morphology. The N_2_ adsorption and desorption isotherm (collected from Quantachrone Autosorb iQ-MP-C) was obtained to derive the surface areas and the pore size distribution of the catalysts. Transmission electron microscopy (TEM) and high-resolution transmission electron microscopy (HRTEM) were performed using a FEI Talos F200S microscope operating at an accelerating voltage of 200 kV. Initially, the fully ground sample was dispersed in an ethanol solution and subjected to ultrasonic homogenization. Subsequently, droplets of the solution were deposited onto a copper mesh coated with a carbon film using a pipette. After drying at room temperature, TEM samples were prepared for analysis. The TEM investigations were carried out at an operating voltage of 200 kV. Additionally, the Transmission Electron Microscope with Probe Corrector (Themis Z, Thermo Fisher Scientific) was performed at an accelerating voltage of 200 kV.

X-ray photoelectron spectroscopy (XPS) measurements were conducted utilizing an Omicron EA 125 Energy Analyzer equipped with a monochromated Al K-alpha source operating at 1486.7 eV. High-resolution core-level component X-ray photoelectron spectroscopy (XPS) scans were conducted utilizing a pass energy of 20 eV in high magnification mode. The entrance and exit slits were set to 6 and 3 mm, respectively, resulting in an overall source and instrumental resolution of 0.6 eV. The methodology for preparing XPS samples involves the following steps: First, a small quantity of the powder sample is positioned on a piece of aluminum foil. Subsequently, a section of double-sided Scotch tape, approximately 5 × 5 mm^2^ in area, is cut and held with tweezers to adhere the sample. The tape along with the sample, is then subjected to gentle pressure using a tablet press until the sample surface is rendered flat and free of contaminants. Finally, the prepared sample is placed on the sample stage for analysis. All spectra were calibrated by using the C 1s (binding energy of 284.8 eV) as a reference point.

2.5. Electrochemical measurements

The electrochemical measurements were performed with a CHI660E electrochemical workstation using a standard three-electrode system at room temperature. Ir cluster@CoO/CeO_2_ and other control samples, the graphite rod, and Hg/HgO electrode were employed as the working, counter, and reference electrodes, respectively. All linear sweep voltammetry (LSV) measurements were performed at a scan rate of 5 mV s^-1^ and were recorded with 85% iR compensation. All the potentials were calculated to the reversible hydrogen electrode (RHE) by the equation E_RHE_ = E_Hg/HgO_ + 0.0591 × pH + 0.098. The Nyquist plots were obtained to investigate the conductivity of catalysts (frequency range: 0.01-10^5^ Hz). The electrochemical surface areas (ECSA) of as-prepared samples were calculated from cyclic voltammetry (CV) curves recorded at a sweep rate from 0.01 to 0.12 V s^-1^ in a non-faradic reaction zone. Use chronopotentiometry to evaluate the stability of electrode materials. For comparison, Pt/C (20 wt%), RuO_2_, and other powder samples were loaded on bare CC in the form of a catalyst ink, which contained a certain content of catalyst, ethanol, and 5 wt% Nafion solution. Faradaic efficiency was calculated by comparing the experimental and theoretical produced amounts of H_2_ and O_2_.

**Turnover Frequency (TOF)**

Consequently, the Turnover Frequency (TOF) of the Ir cluster@CoO/CeO_2_ electrocatalyst was calculated using equation (1).

$TOF=\frac{number of O_{2}\mathrm{turnovers}}{number of metal ions}$(1)
 The number of total O_2_ turnovers was calculated using the current density (j) from the OER-LSV polarization:

$$Number of O_{2} turnovers:=\left( j\frac{\mathrm{mA}}{cm^{2}} \right)\left( \frac{1 \frac{C}{s}}{1000 mA} \right)\left( \frac{1 mol e^{-}}{96485 C} \right)\left( \frac{1 molO_{2}}{4 mole^{-}} \right)\left( \frac{6.02\times{10}^{23}\mathrm{mol}O_{2}}{1 mol O_{2}} \right)=1.56 \times{10}^{15} \frac{\frac{O_{2}}{s}}{cm^{2}}\mathrm{per}\frac{\mathrm{mA}}{cm^{2}}$$

(2)

The upper limit of active sites density for Ir cluster@CoO/CeO_2_:

$$= \frac{0.27 mg cm^{-2}\times10^{-3}\times2.23\%\times6.023\times{10}^{23}}{192.2 g mol^{-1}} per cm^{2}$$

$=0.019\times10^{18} Ir site per cm^{2}$(3)

The TOF of Ir cluster@CoO/CeO_2_ can be obtained via the current density from the OER LSV polarization curves and according to:

$TOF=\frac{1.56 \times{10}^{15}}{0.019\times10^{18}} \left| j \right|$(4)

**Mass activity (MA) calculation:**

Mass activity (A g_metal_^-1^) was derived from the current density (mA cm^-2^) normalized by the mass loading (0.27 mg cm^-2^) and per metal at a certainly applied overpotential. The following equation exhibited the mass activity of Ir cluster@CoO/CeO_2_:

$Mass activity=\frac{\left| j \right|}{0.27\times2.23\%}$ (5)

**DFT calculation**

The Vienna Ab initio Simulation Package (VASP) was carried out to perform the density functional theory (DFT) calculations. The electronic exchange and correlation were described within the generalized gradient approximation (GGA) using the Perdew-Burke-Ernzerhof (PBE) functional. The interactions between ion cores and valence electrons were described by the projector augmented wave (PAW) method. The DFT with the Hubbard-U framework (DFT + U) calculated the energetics of metal and account for strongly localized d-electrons for metal. Through linear response theory to get the Hubbard-U correction terms (at U_eff_ (Co) = 3.32 eV). The cutoff energy was set as 500 eV. The Monkhorst-Pack grid of 3 × 3 × 1 was employed in k-points. The energy convergence tolerance and forces for geometry optimization were 10^-6^ eV per atom and < 0.01 eV Å^-1^, respectively.

**Section 2. Supporting Figures and Tables**


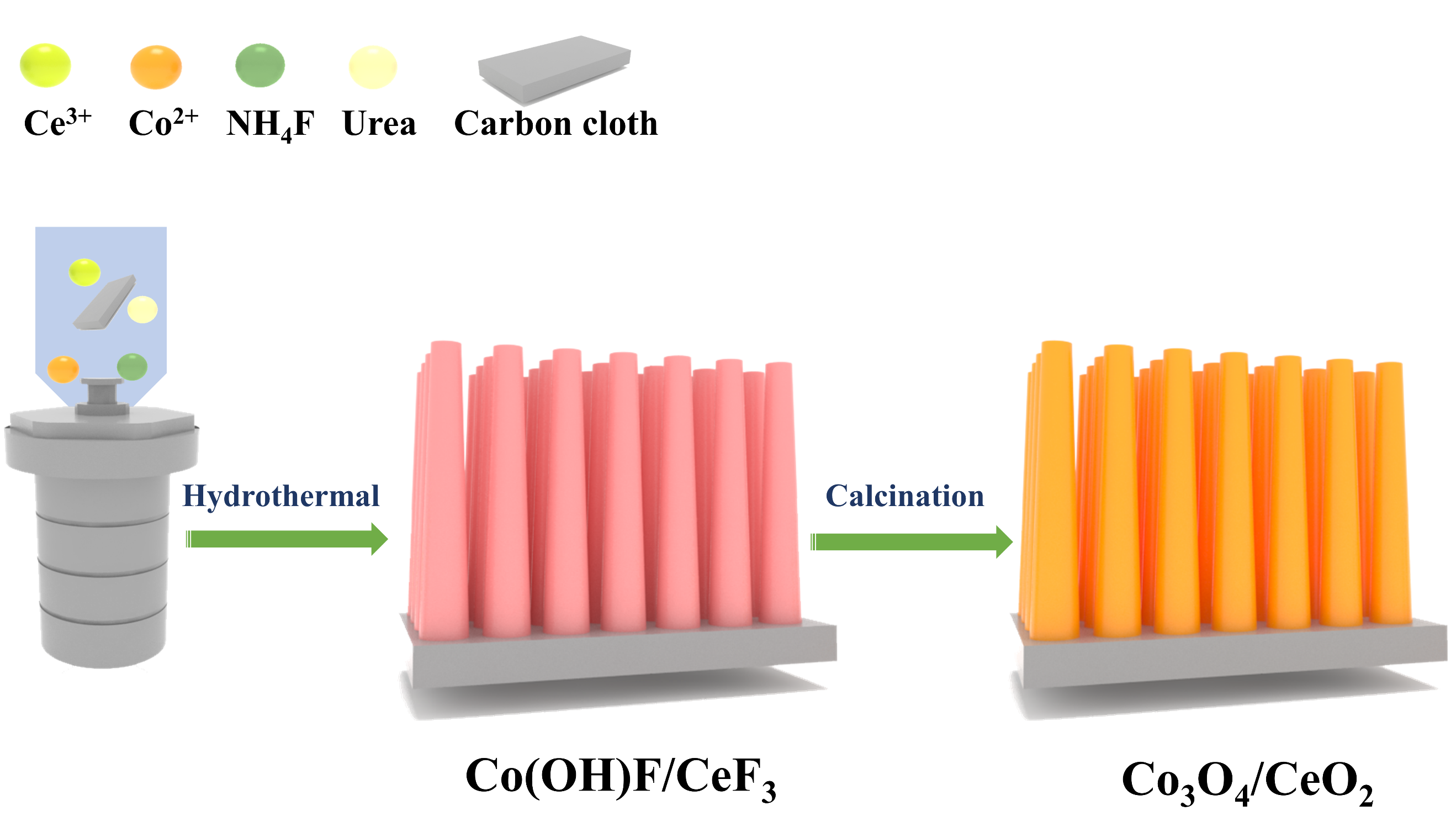


**Figure S1.** Schematic diagram of the synthesis of the Co_3_O_4_/CeO_2_.


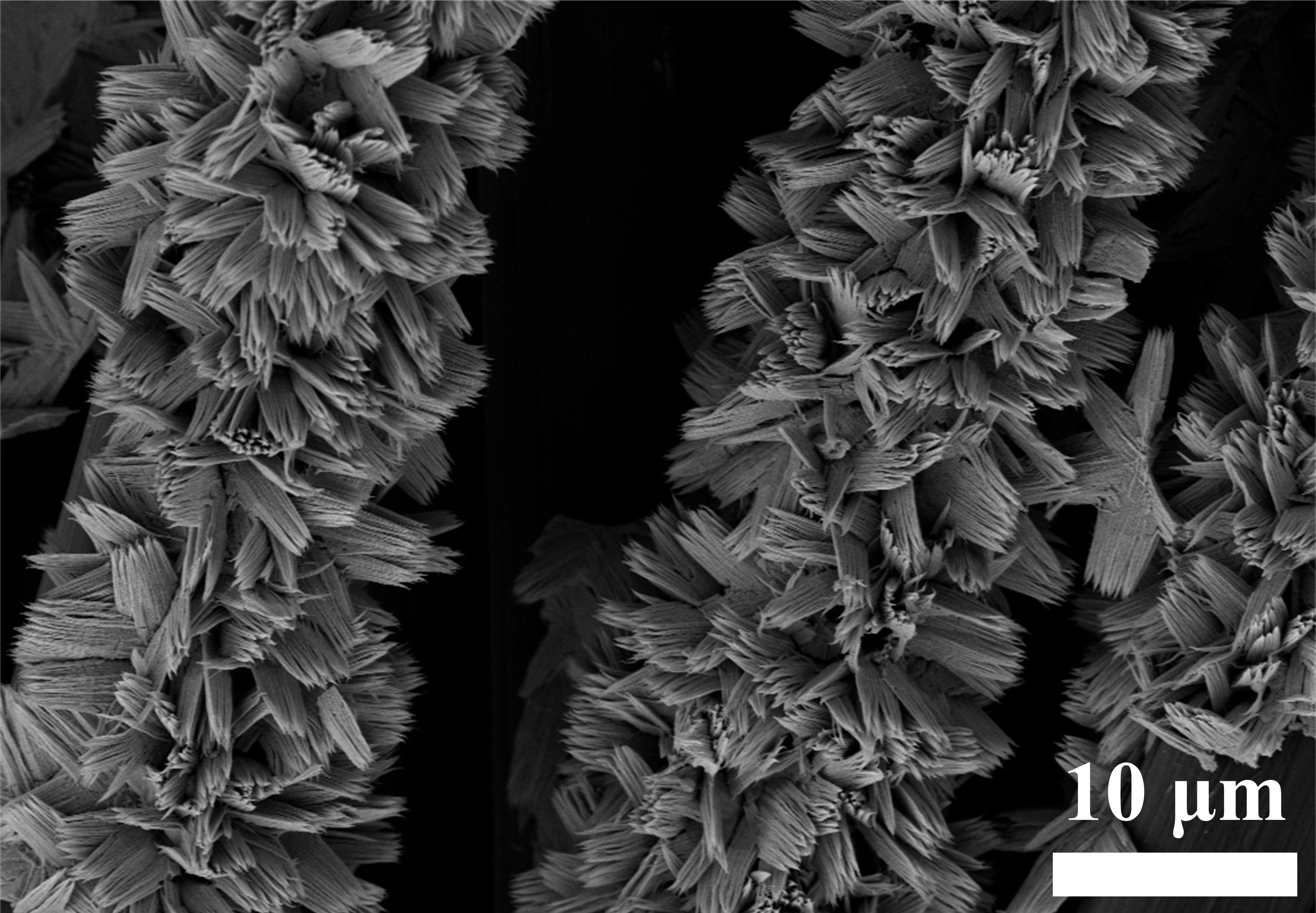


**Figure S2.** SEM images of Ir cluster@CoO/CeO_2_.

**
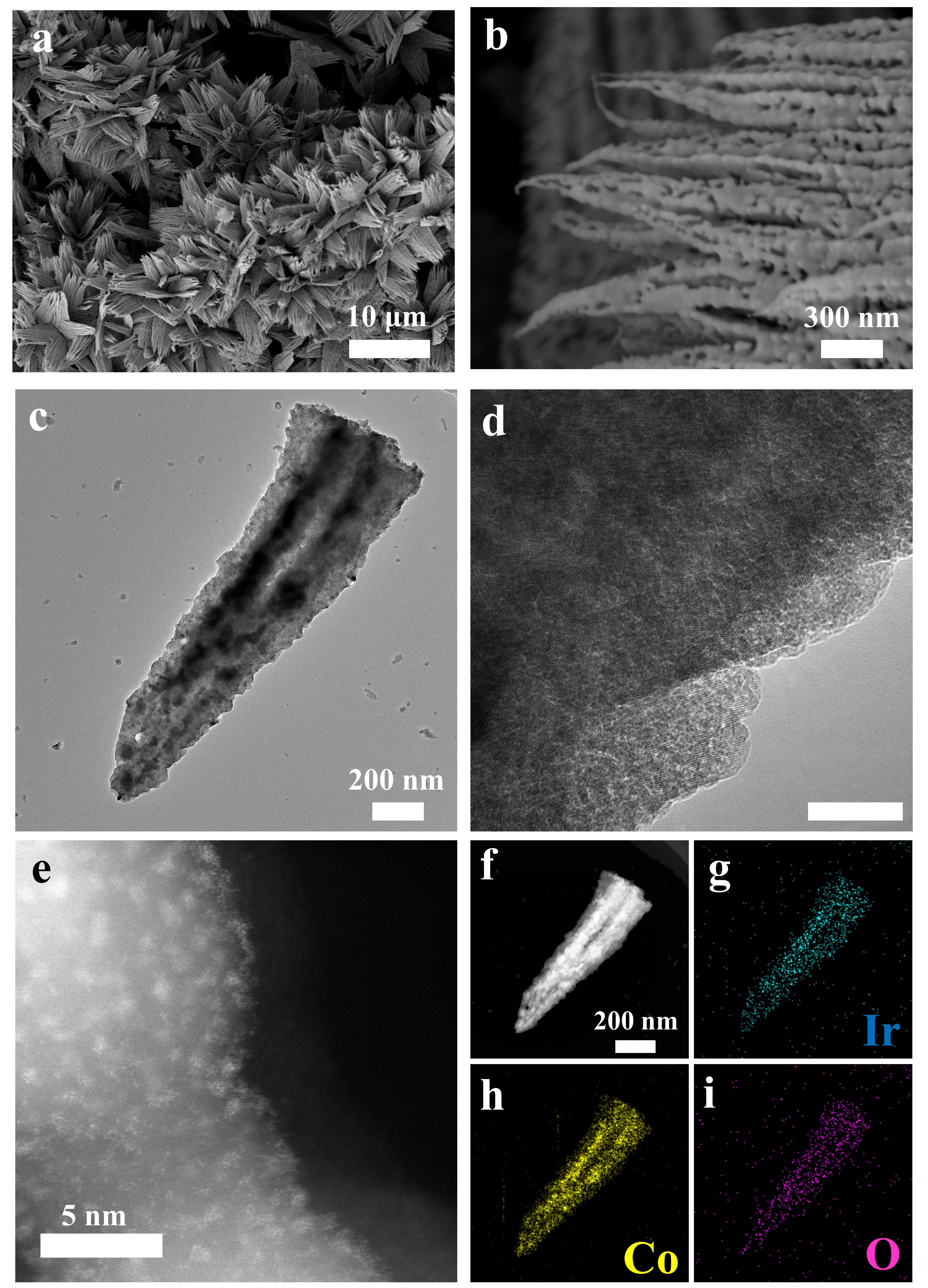
**

**Figure S3.** SEM and TEM images of Ir cluster@CoO.

**
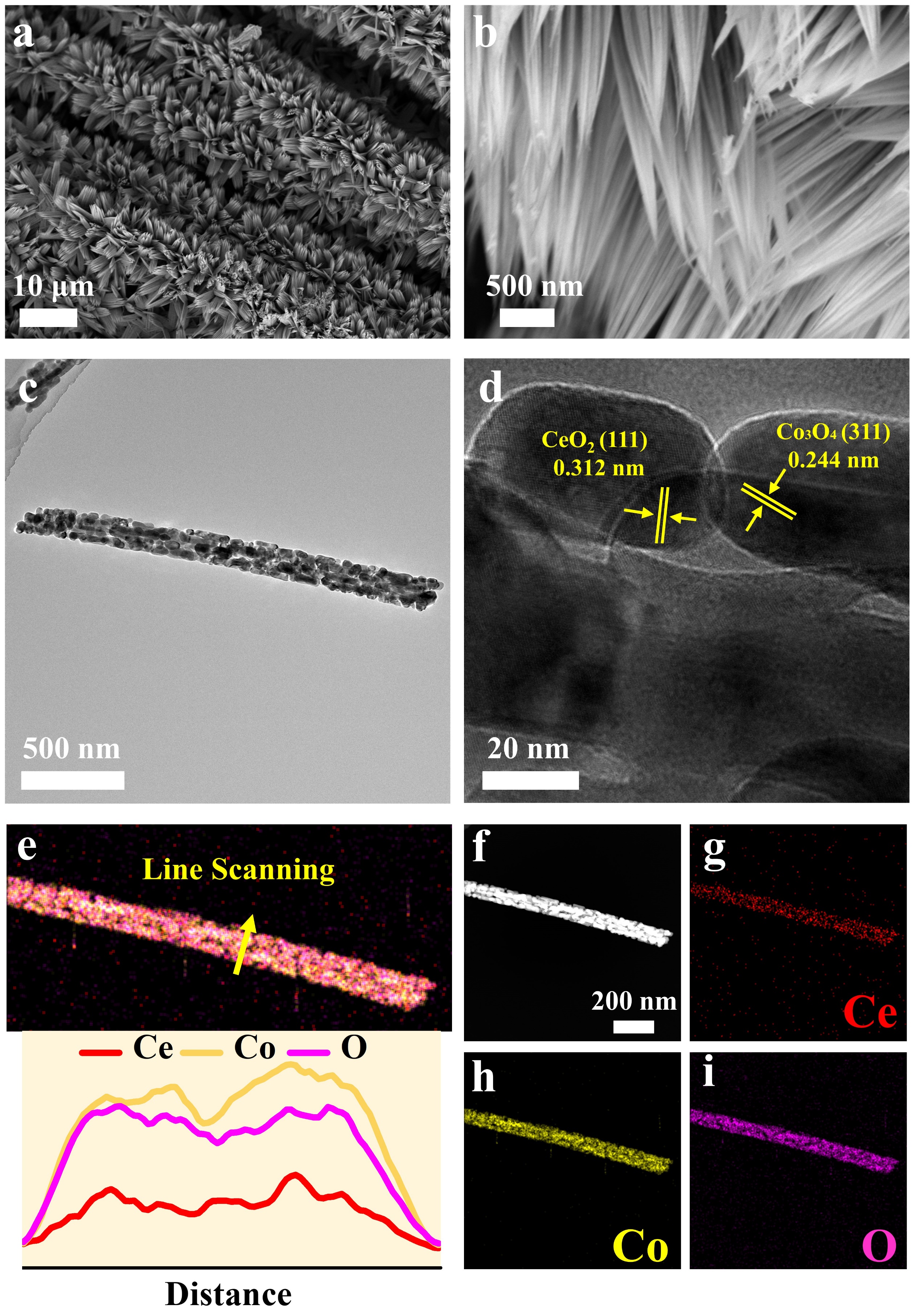
**

**Figure S4.** SEM and TEM images of Co_3_O_4_/CeO_2_.


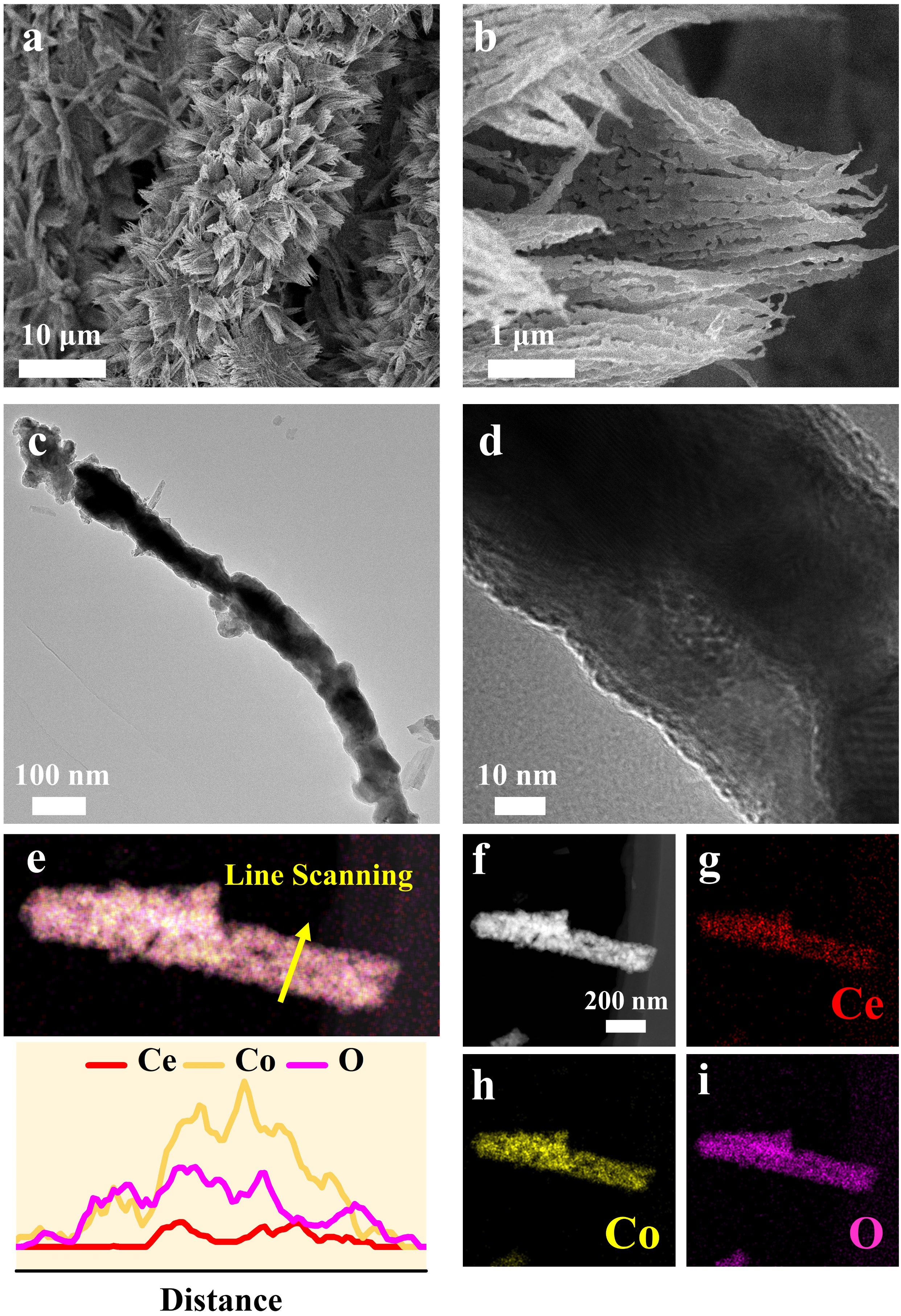


**Figure S5.** SEM and TEM images of CoO/CeO_2_.


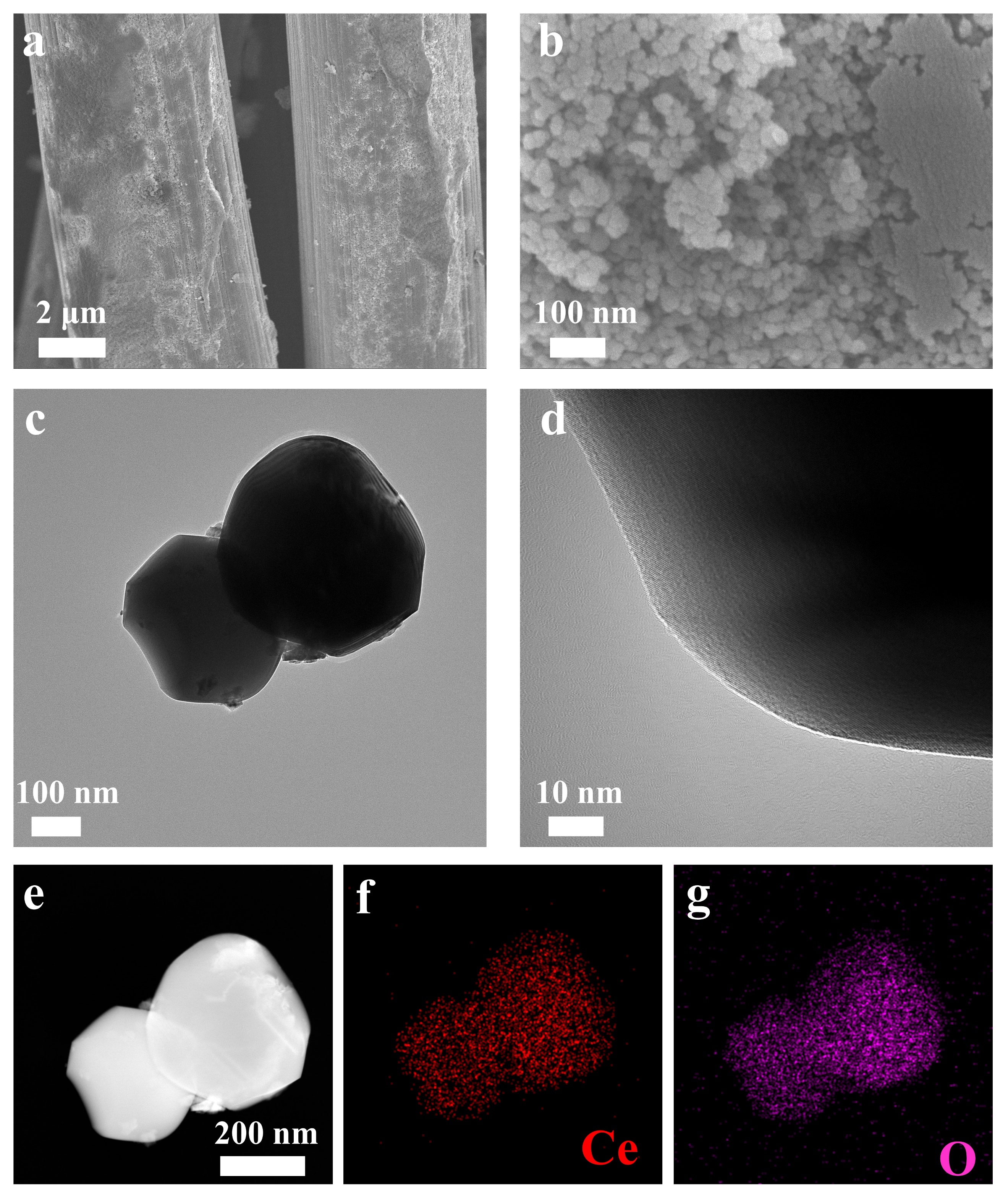


**Figure S6.** SEM and TEM images of CeO_2_.


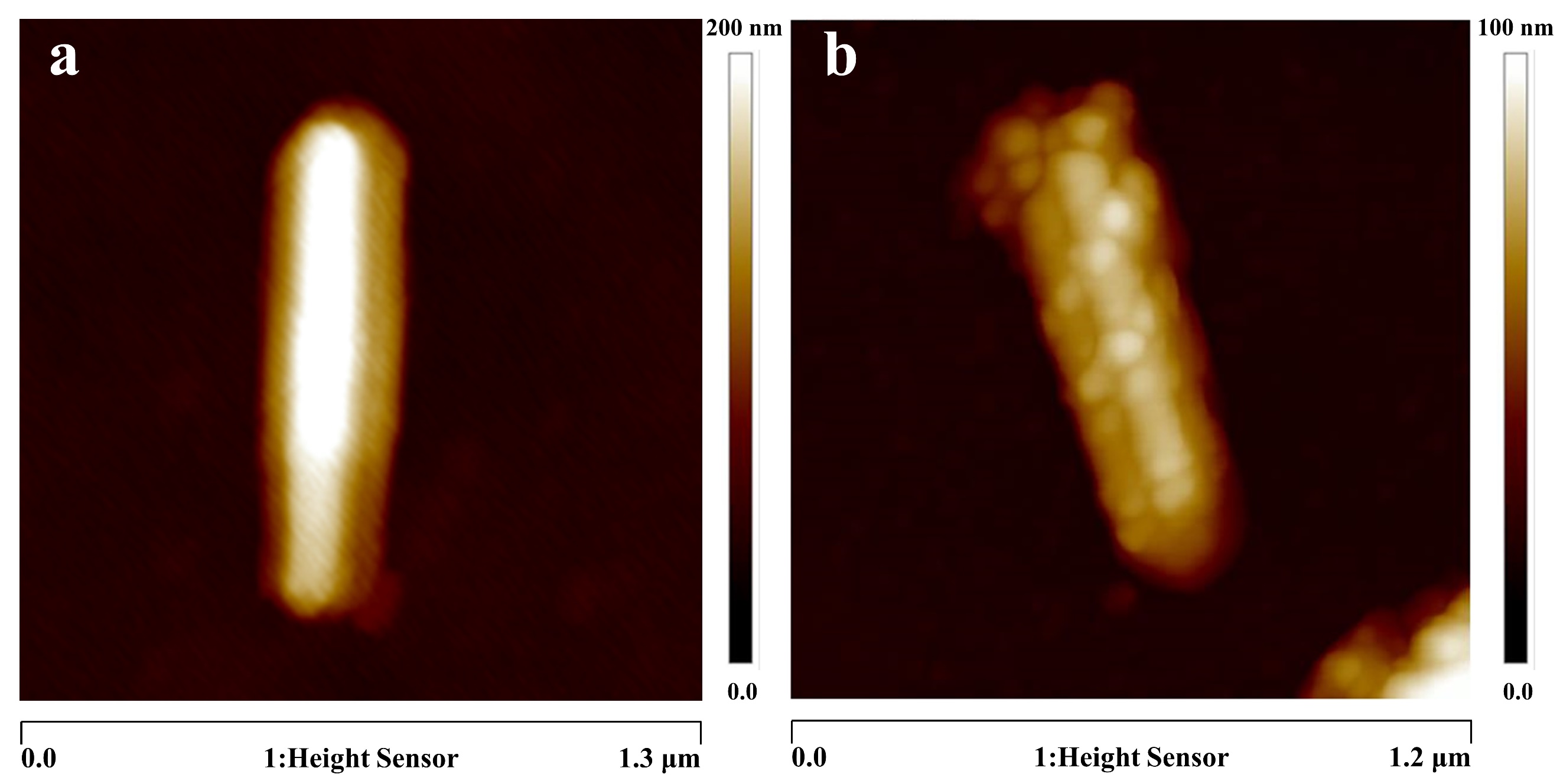


**Figure S7.** AFM images of (a) Ir ^3+^@Co_3_O_4_/CeO_2_, (b) Ir cluster@CoO/CeO_2_.


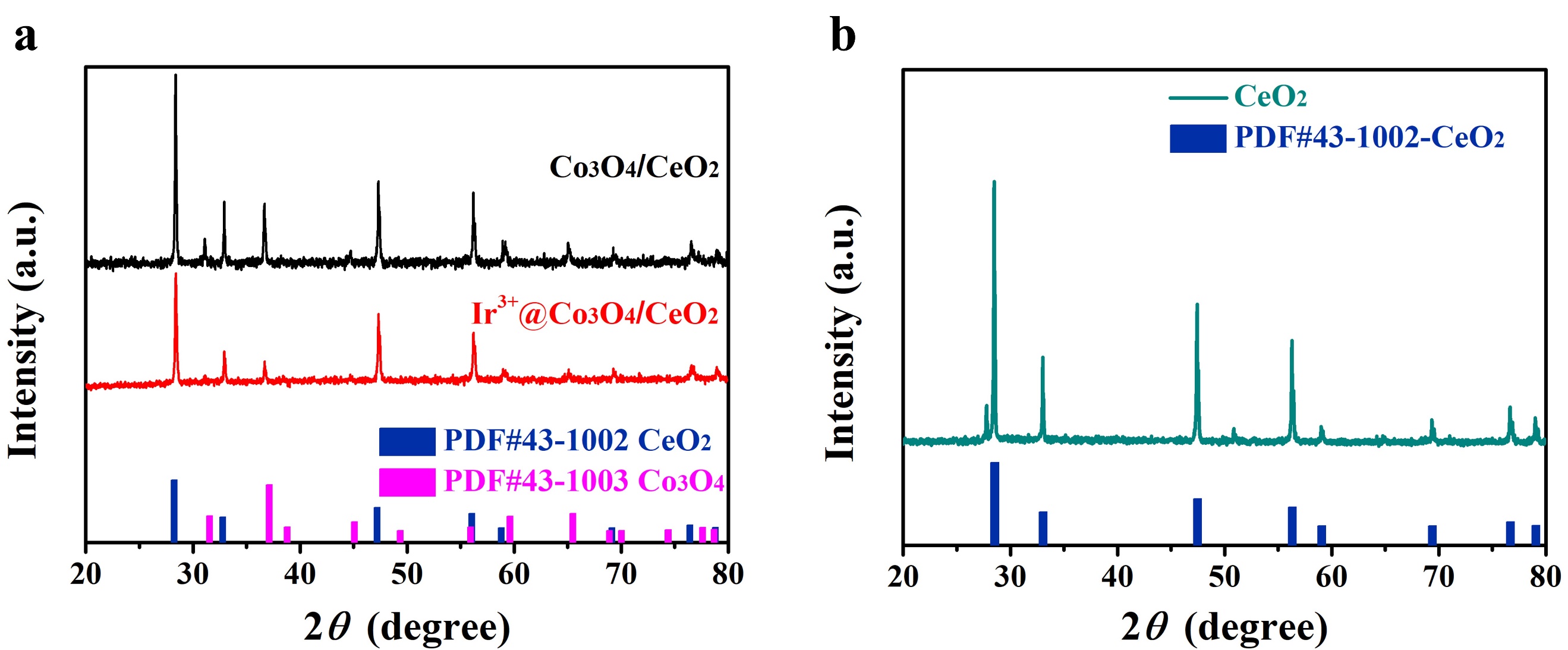


**Figure S8.** XRD pattern of (a) Co_3_O_4_/CeO_2_ before and after Ir cation exchange, (b) CeO_2_.


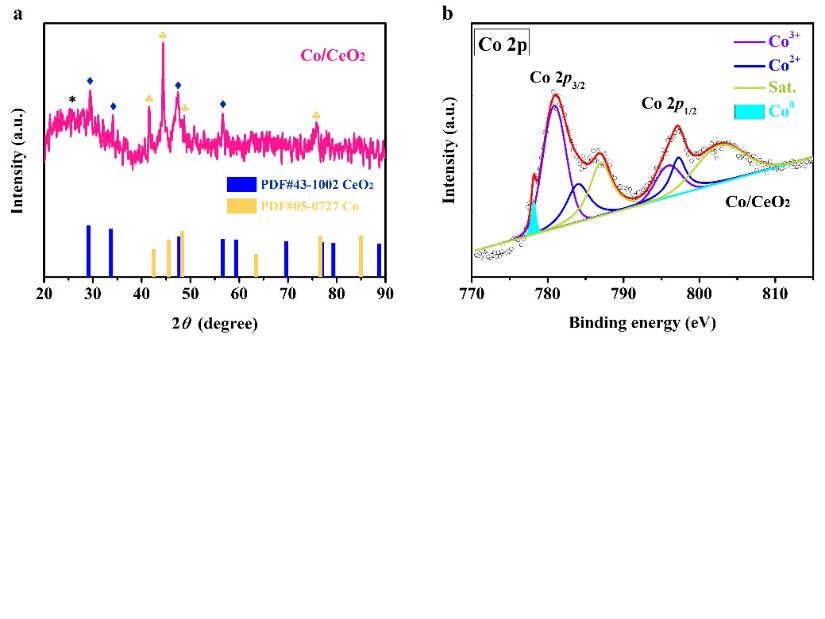


**Figure S9.** XRD pattern of Co/CeO_2_.


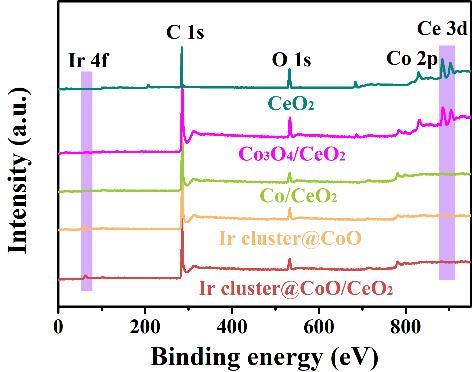


**Figure S10.** XPS survey spectrum of different catalysts.


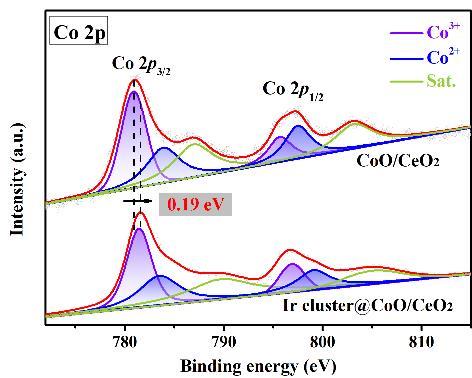


**Figure S11.** XPS spectra of Co 2p in CoO/CeO_2_ and Ir cluster@CoO/CeO_2_.

Compared with CoO/CeO_2_ the binding energy of Co 2p in the Ir cluster@CoO/CeO_2_ was positively shifted about 0.19 eV, implying that the Co lost electrons and the couple with Ir species could promote electronic redistribution.


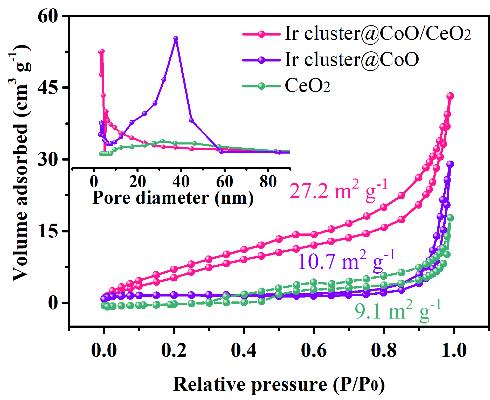


**Figure S12.** N_2_ adsorption/desorption isotherms of the Ir cluster@CoO/CeO_2_ and other catalysts.


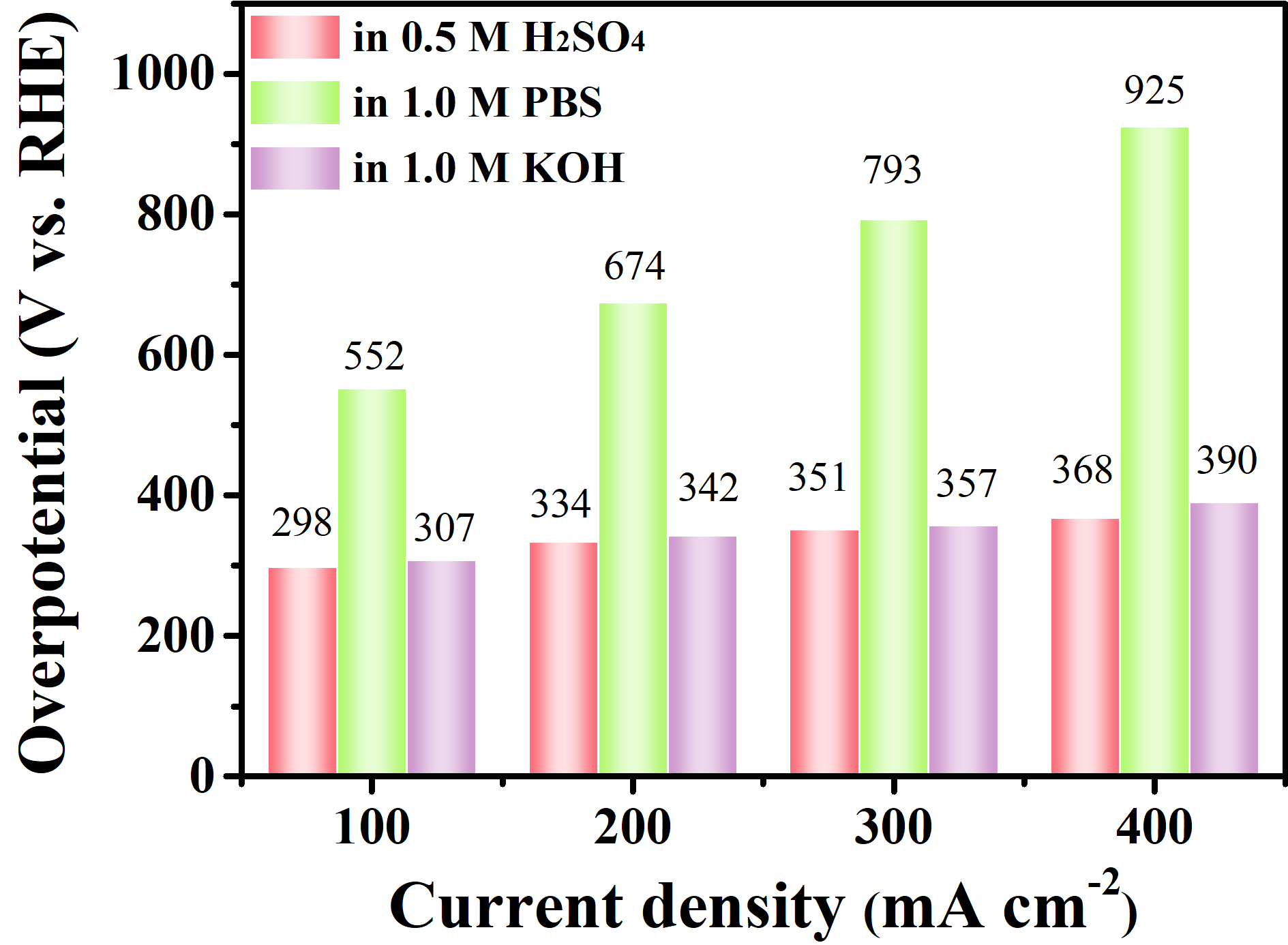


**Figure S13.** Overpotential comparison of Ir cluster@CoO/CeO_2_ at various current densities (at 100, 200, 300, and 400 mA cm^-2^) for OER in 0.5 M H_2_SO_4_, 1.0 M PBS, and 1.0 M KOH.


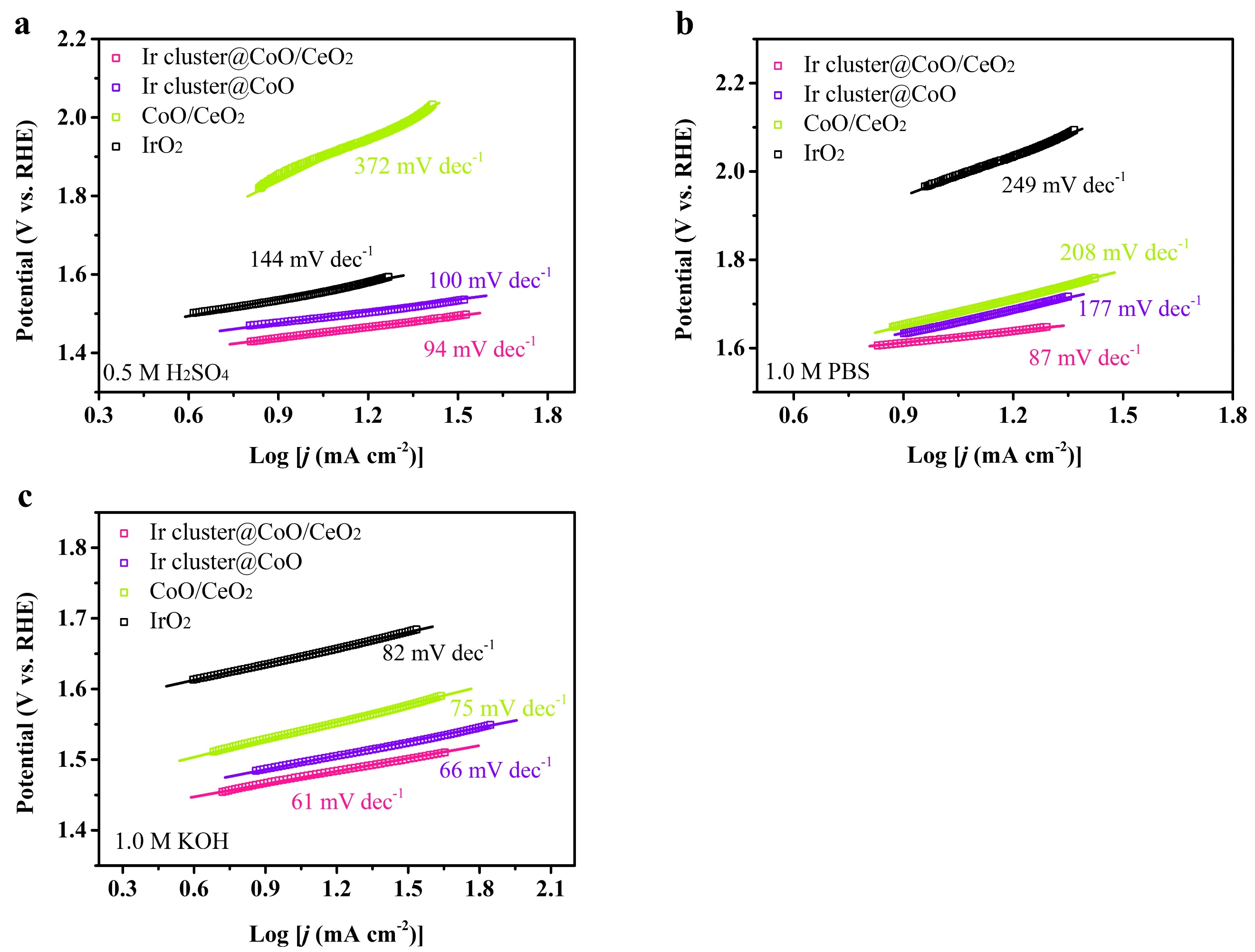


**Figure S14.** Tafel slope values for OER of Ir cluster@CoO/CeO_2_, Ir cluster@CoO, CoO/CeO_2_, and IrO_2_ at 10 mA cm^-2^ under 0.5 M H_2_SO_4_, 1.0 M PBS, and 1.0 M KOH, respectively.


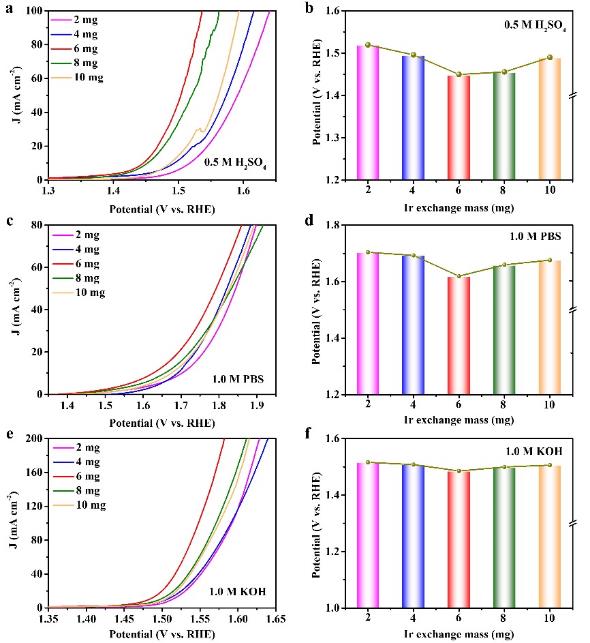


**Figure S15.** Comparison of OER performance of Ir cluster@CoO/CeO_2_ prepared by ion exchange with iridium cations of different concentrations. The OER LSV curves in (a) 0.5 M H_2_SO_4_, (c) 1.0 M PBS, and (e) 1.0 M KOH electrolytes. Comparison of OER overpotential in 10 mA cm^-2^ in (b) 0.5 M H_2_SO_4_, (d) 1.0 M PBS, and (f) 1.0 M KOH electrolytes.


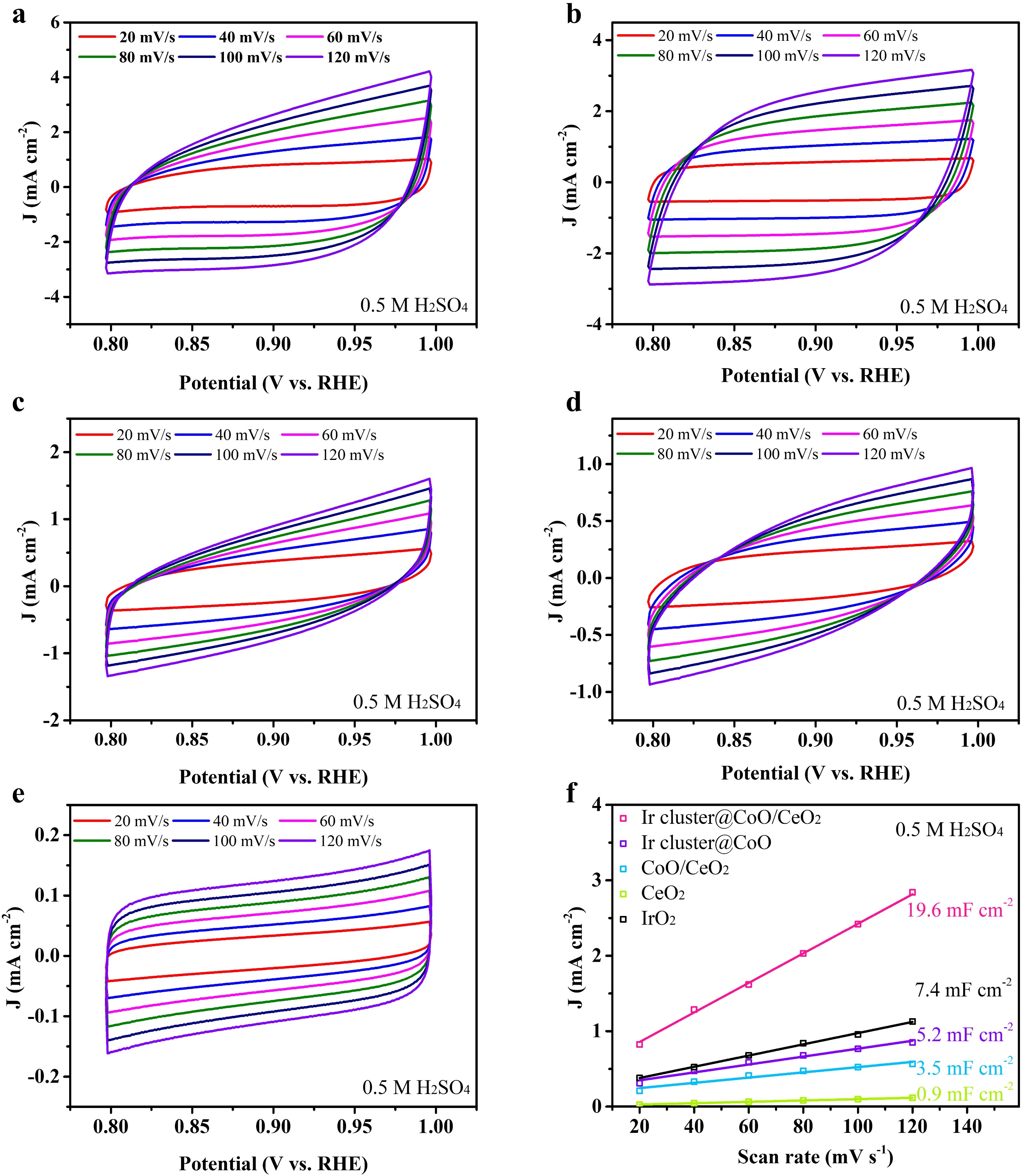


**Figure S16.** CV curves for OER of (a) Ir cluster@CoO/CeO_2_, (b) IrO_2_, (c) Ir cluster@CoO, (d) CoO/CeO_2_, and (e) IrO_2_ at increasing scan rates from 20 to 120 mV s^−1^ in 0.5 M H_2_SO_4_. (f) C_dl_ of the corresponding samples.


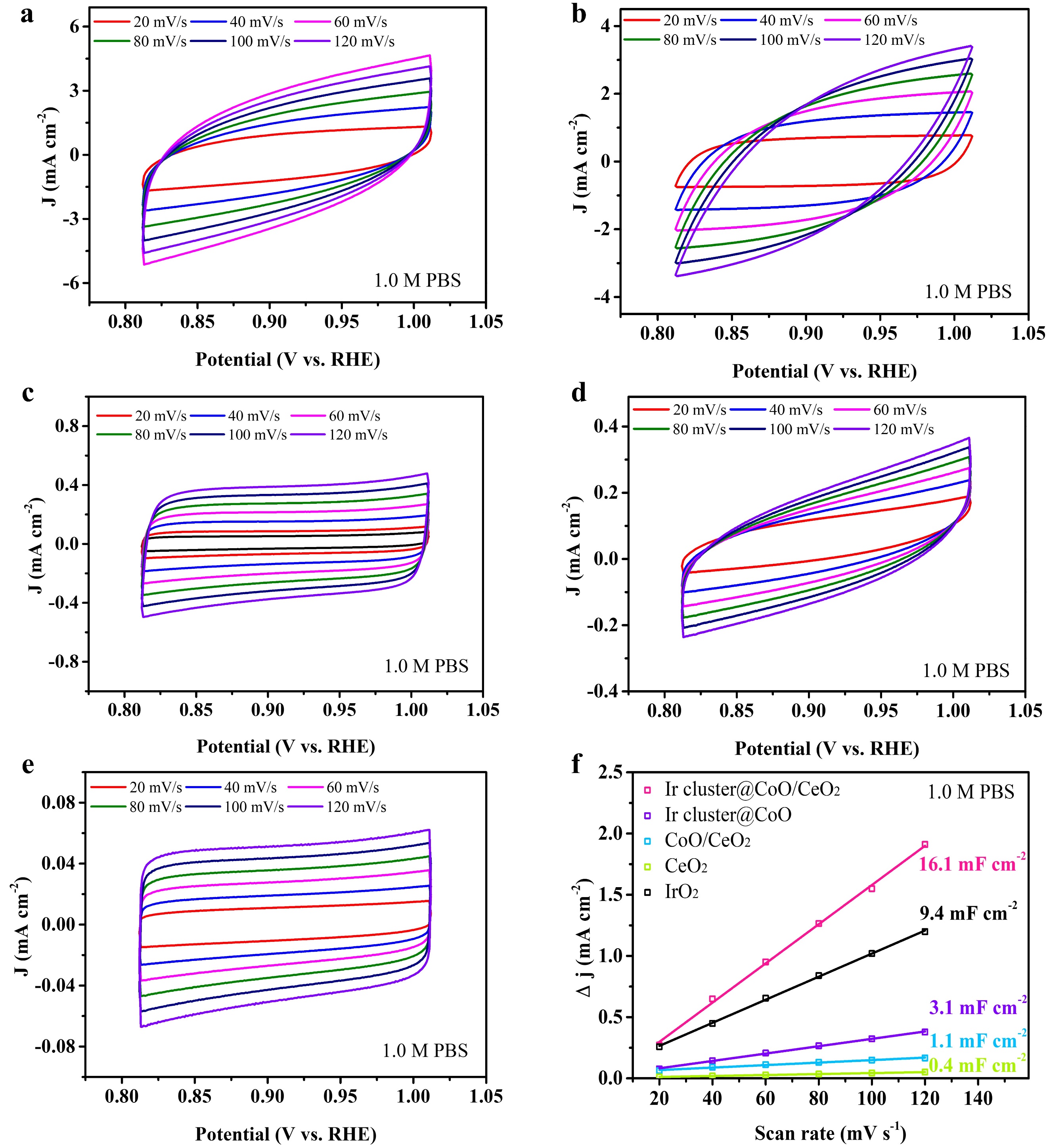


**Figure S17.** CV curves for OER of (a) Ir cluster@CoO/CeO_2_, (b) IrO_2_, (c) Ir cluster@CoO, (d) CoO/CeO_2_, and (e) IrO_2_ at increasing scan rates from 20 to 120 mV s^−1^ in 1.0 M PBS. (f) C_dl_ of the corresponding samples.


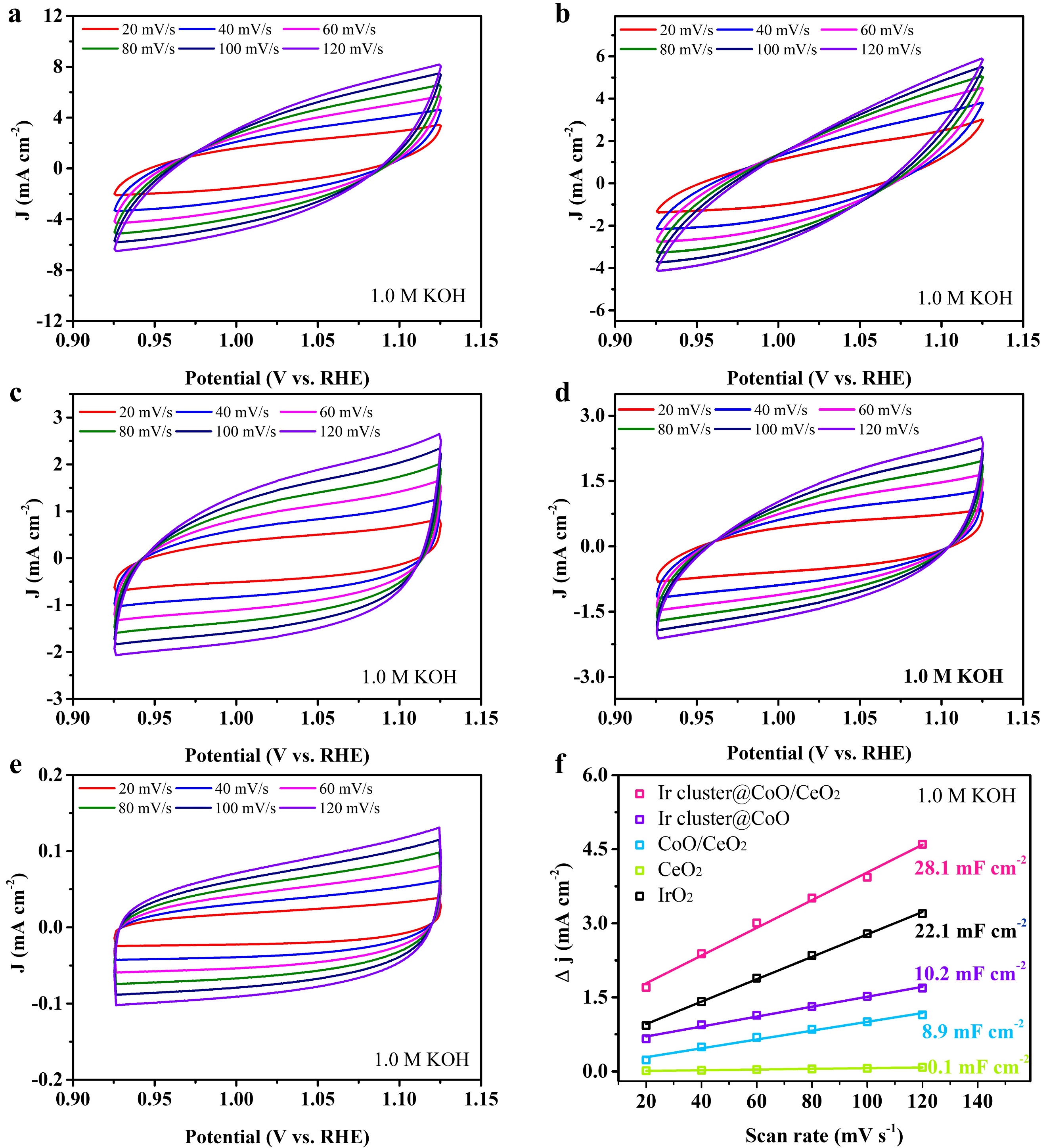


**Figure S18.** CV curves for OER of (a) Ir cluster@CoO/CeO_2_, (b) IrO_2_, (c) Ir cluster@CoO, (d) CoO/CeO_2_, and (e) IrO_2_ at increasing scan rates from 20 to 120 mV s^−1^ in 1.0 M KOH. (f) C_dl_ of the corresponding samples.

**
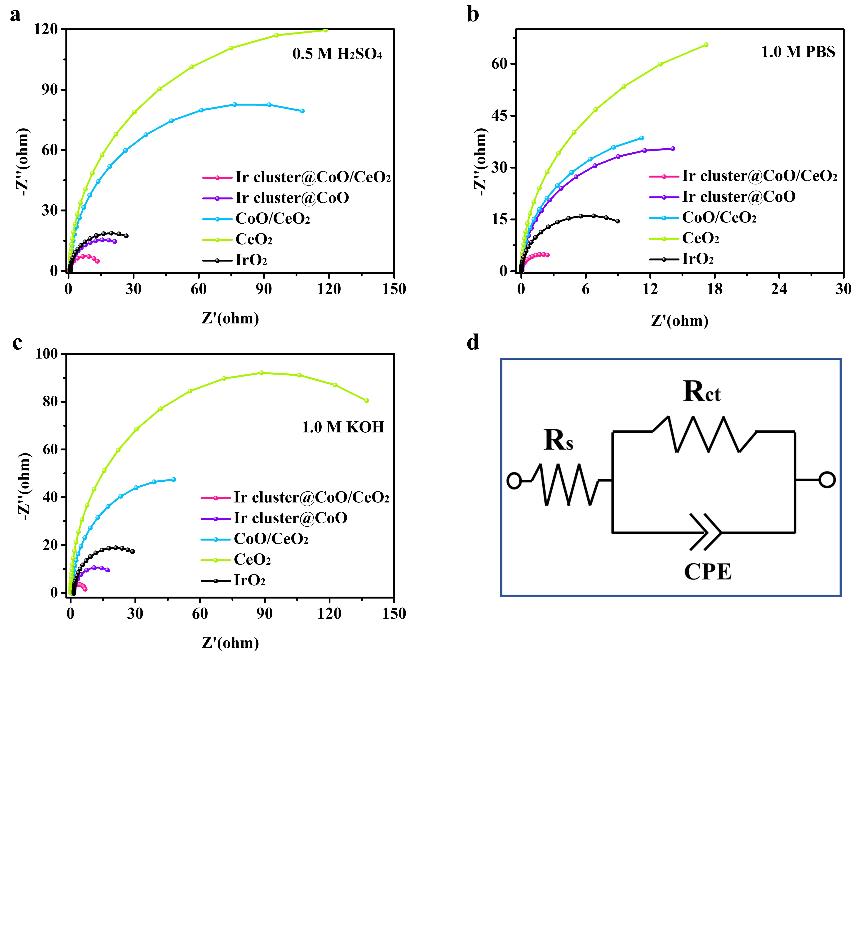
**

**Figure S19.** EIS curves during OER for Ir cluster@CoO/CeO_2_, Ir cluster@CoO, CoO/CeO_2_, and IrO_2_ at 10 mA cm^-2^ under (a) 0.5 M H_2_SO_4_, (b) 1.0 M PBS, and (c) 1.0 M KOH, respectively. (d) The equivalent circuit diagram to fit all the EIS data.


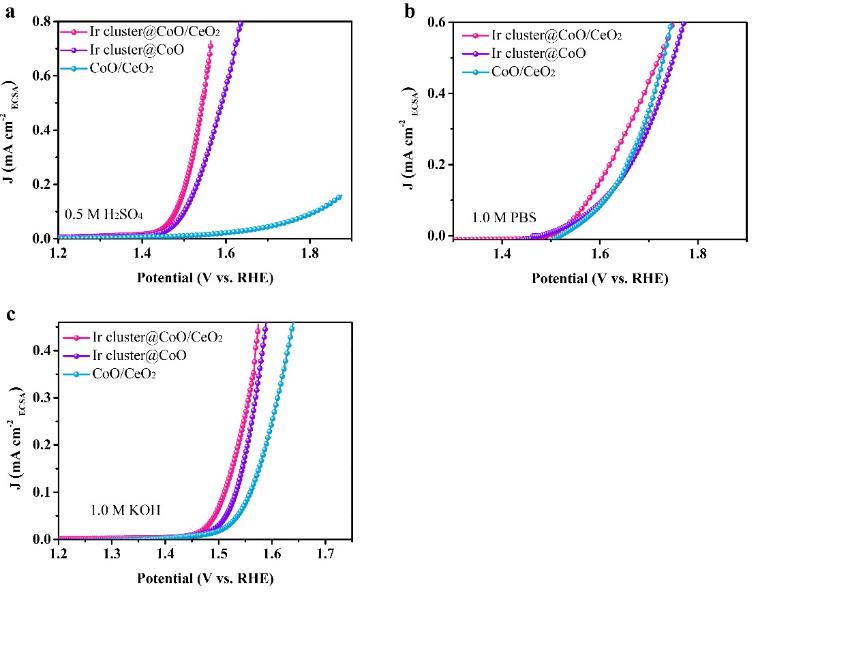


**Figure S20.** ECSA-normalized LSVs of Ir cluster@CoO/CeO_2_, Ir cluster@CoO, and CoO/CeO_2_ for OER in different electrolyte solutions.


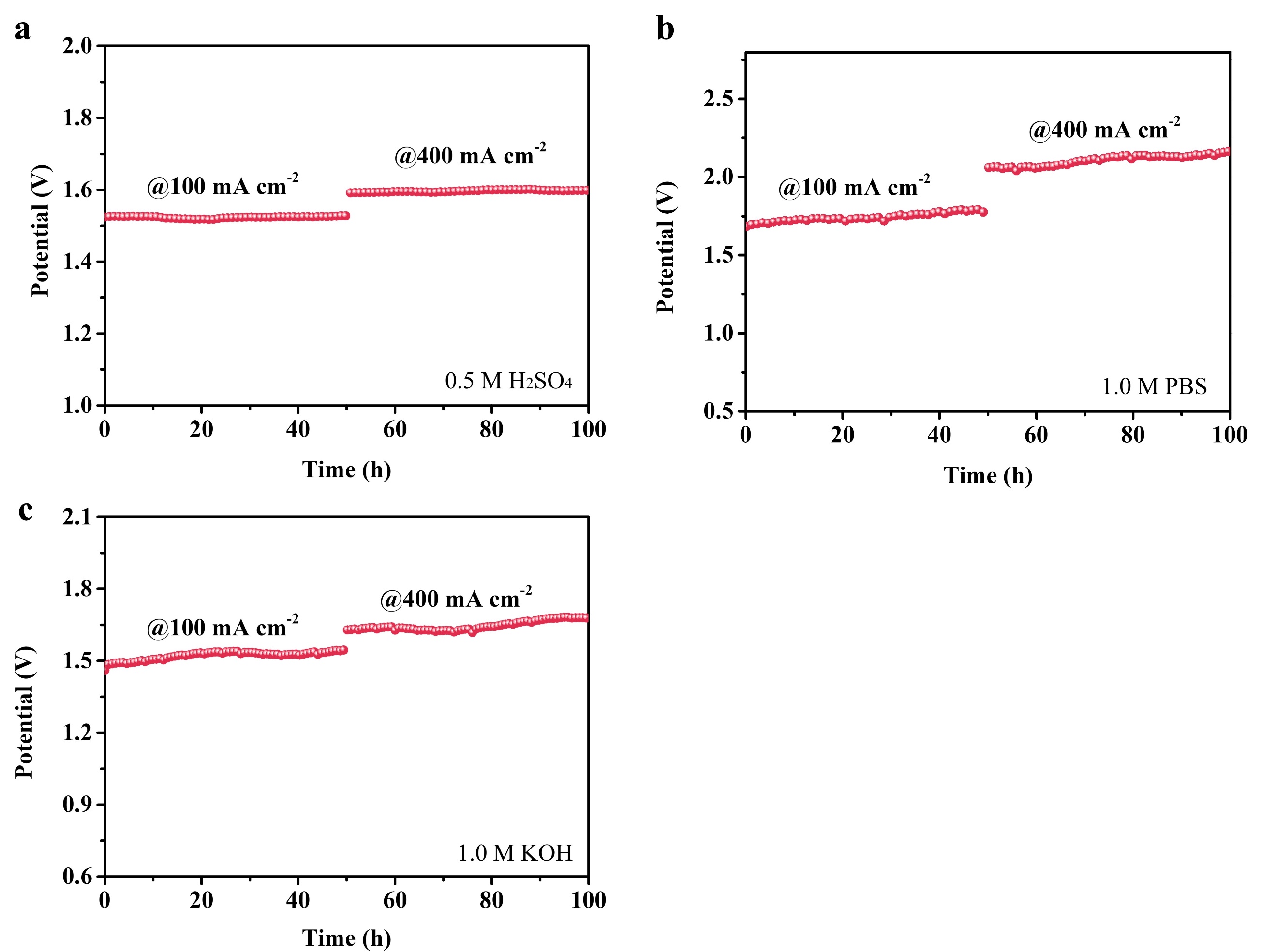


**Figure S21.** (c) Chronopotentiometric analysis of the stability test for OER of Ir cluster@CoO/CeO_2_ at constant high current densities of 100 and 400 mA cm^-2^ in (a) 0.5 M H_2_SO_4_, (b) 1.0 M PBS, and (c) 1.0 M KOH electrolytes, respectively.


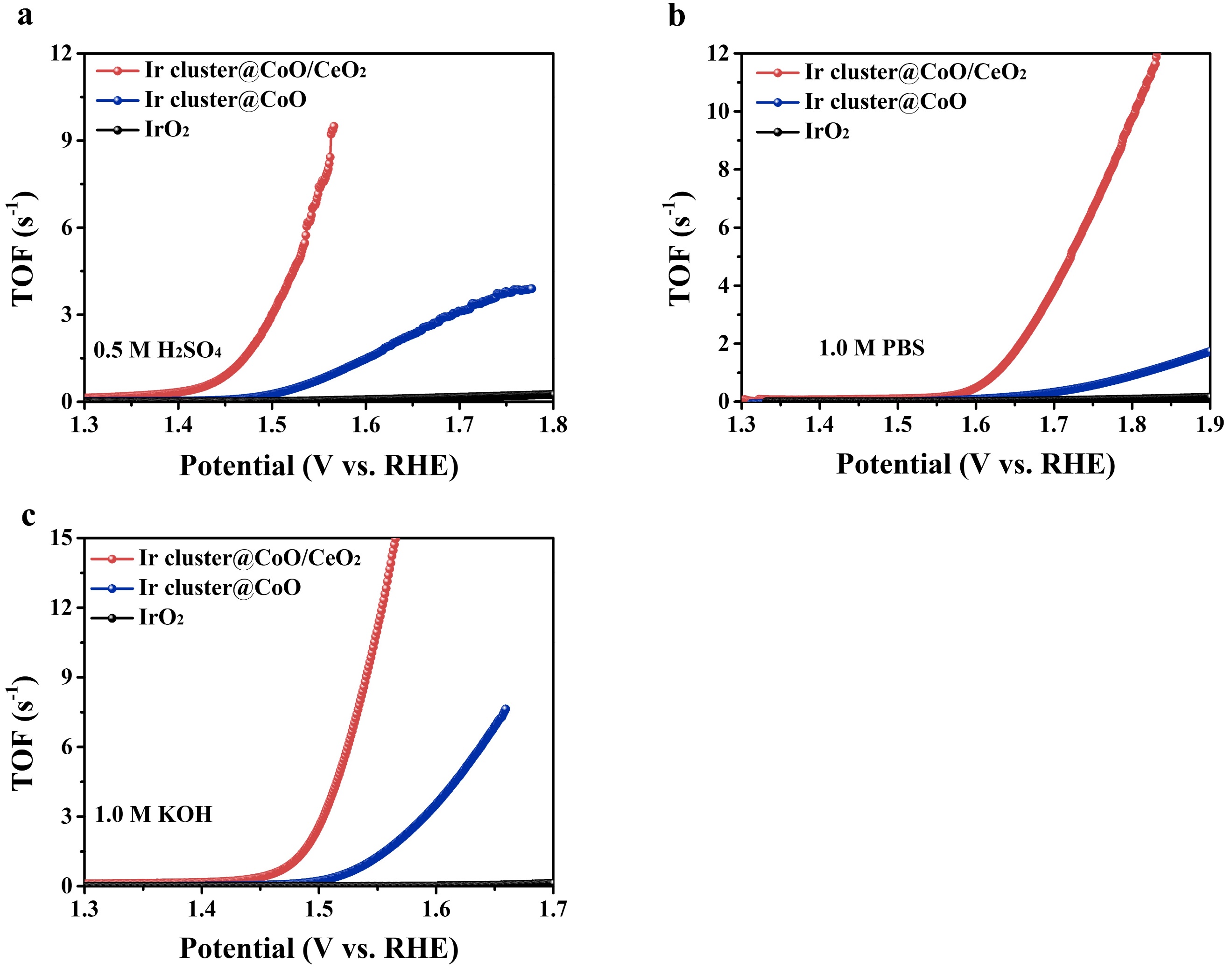


**Figure S22.** The TOF-normalized LSV curves for OER in (a) 0.5 M H_2_SO_4_, (b) 1.0 M PBS, and (c) 1.0 M KOH.


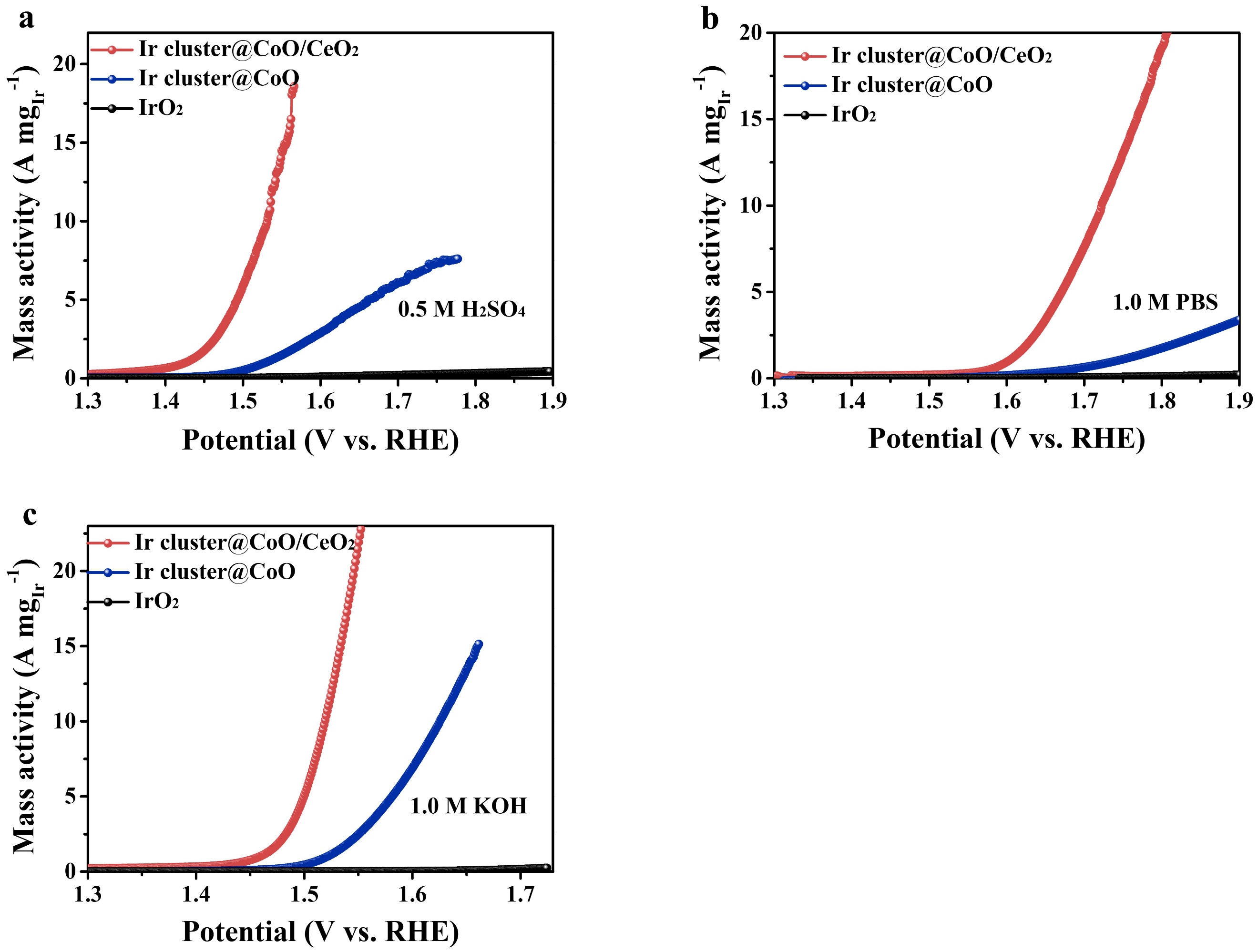


**Figure S23.** The mass-normalized LSV curves for OER in (a) 0.5 M H_2_SO_4_, (b) 1.0 M PBS, and (c) 1.0 M KOH.


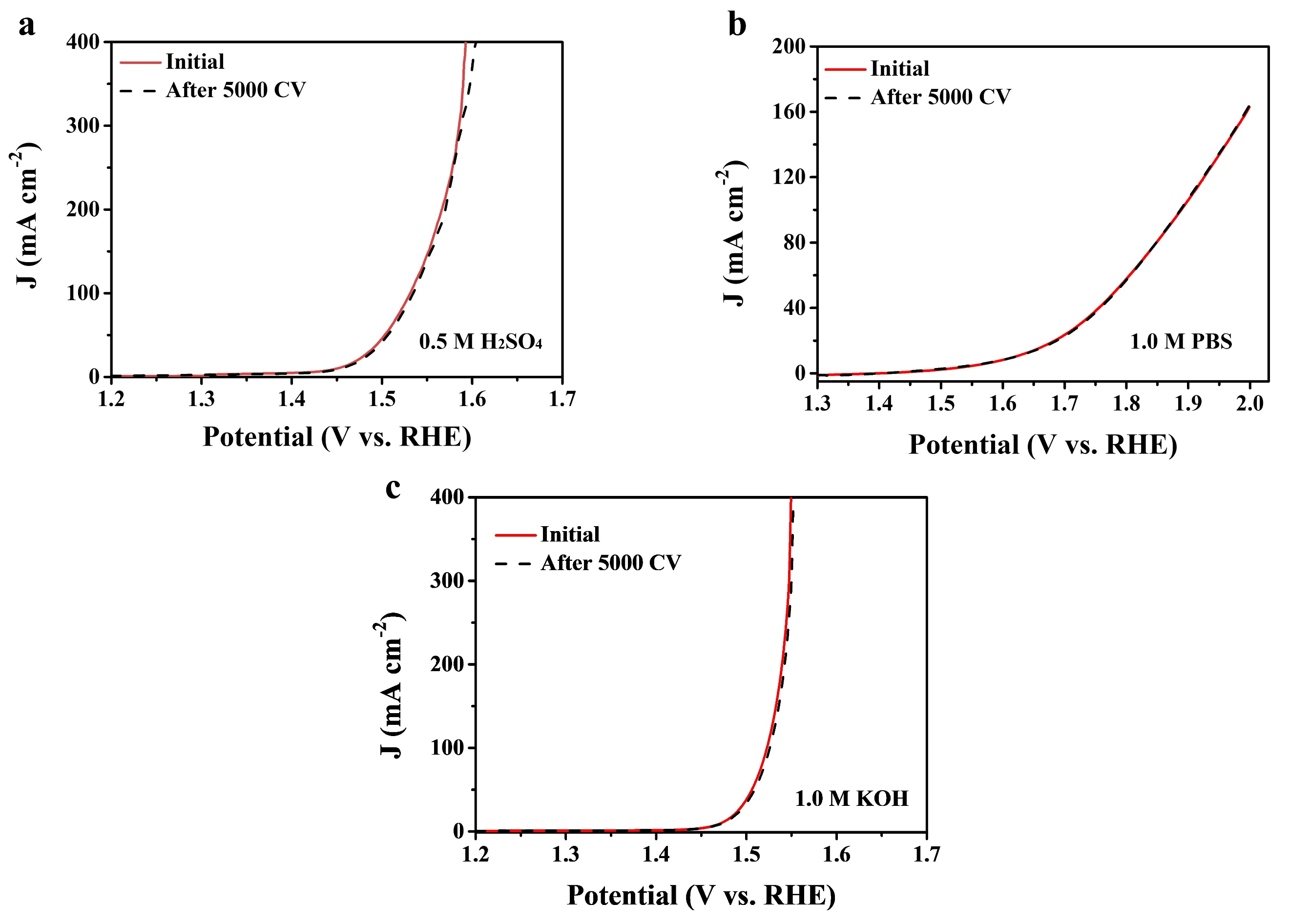


**Figure S24.** LSV cycling stability test of Ir cluster@CoO/CeO_2_ corresponding to the initial and 5000 CV cycles in (a) 0.5 M H_2_SO_4_, (b) 1.0 M PBS, and (c) 1.0 M KOH electrolytes, respectively. Under the rigorous cycling process, very small current density deviation indicates the superstable electrode material.


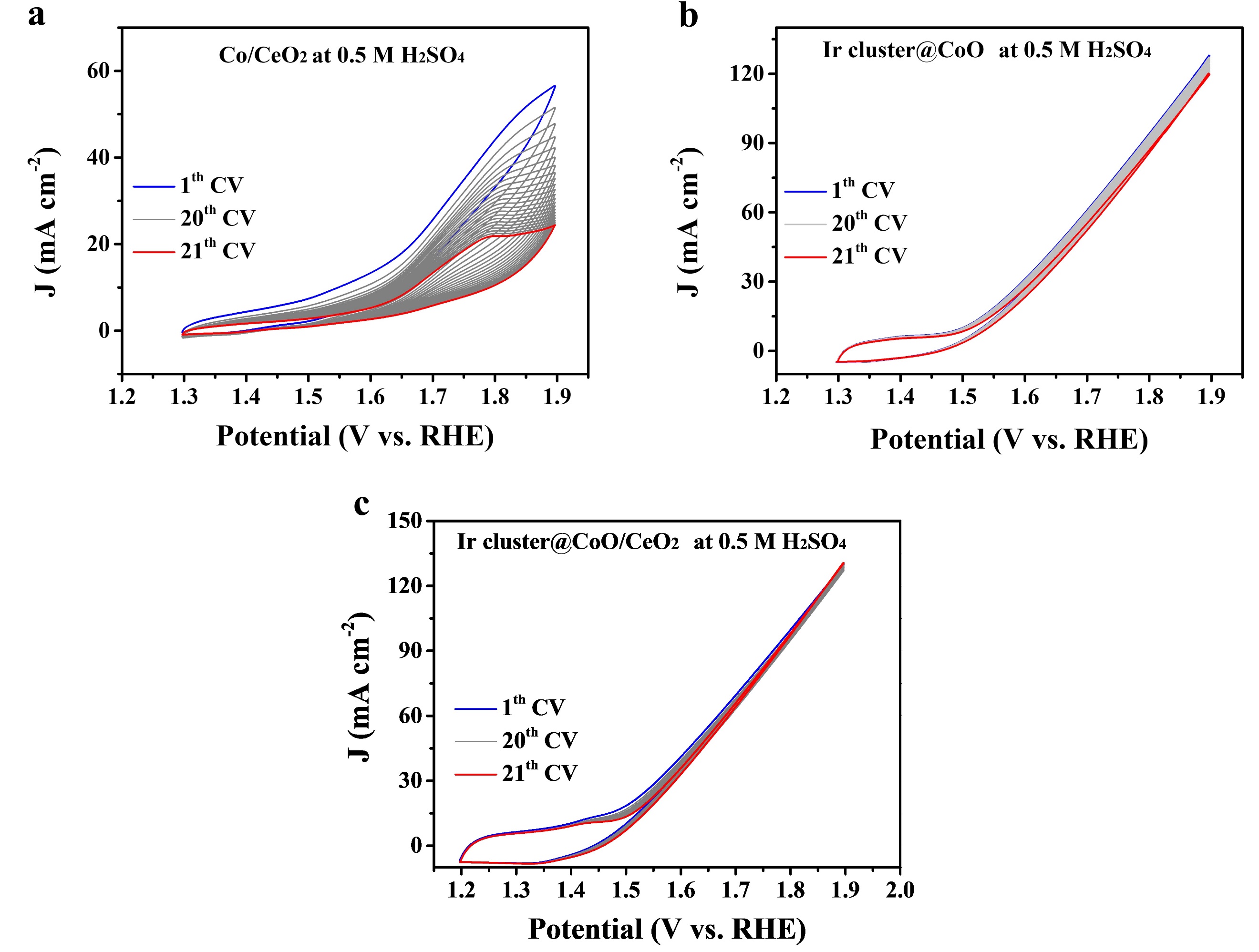


**Figure S25.** CV curves for OER of (a) CoO/CeO_2_, (b) Ir cluster@CoO, and (c) Ir cluster@CoO/CeO_2_ at 0.5 M H_2_SO_4_.

**
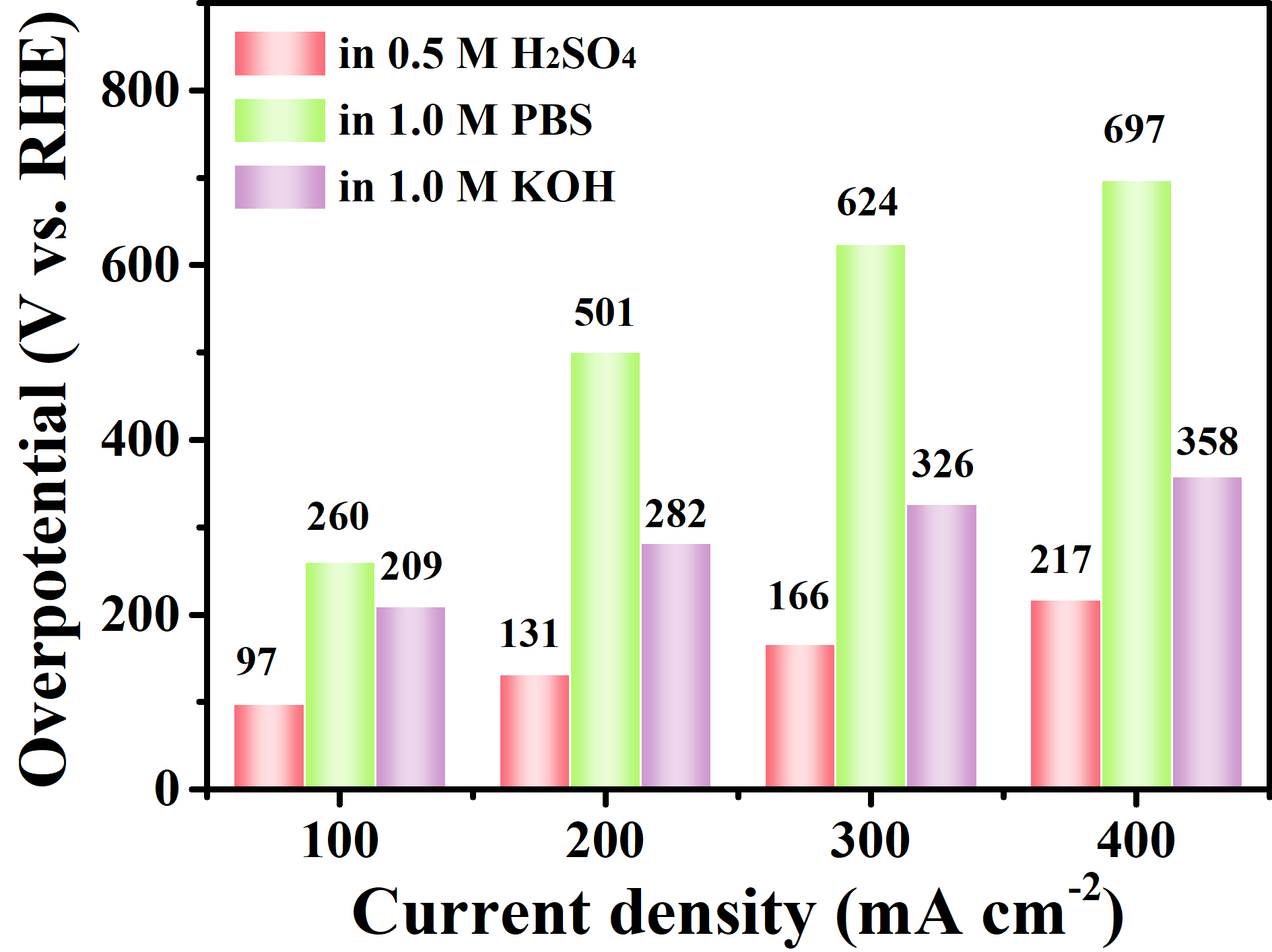
**

**Figure S26.** Overpotential comparison of Ir cluster@CoO/CeO_2_ at various current densities (at 100, 200, 300, and 400 mA cm^-2^) for HER in 0.5 M H_2_SO_4_, 1.0 M PBS, and 1.0 M KOH.


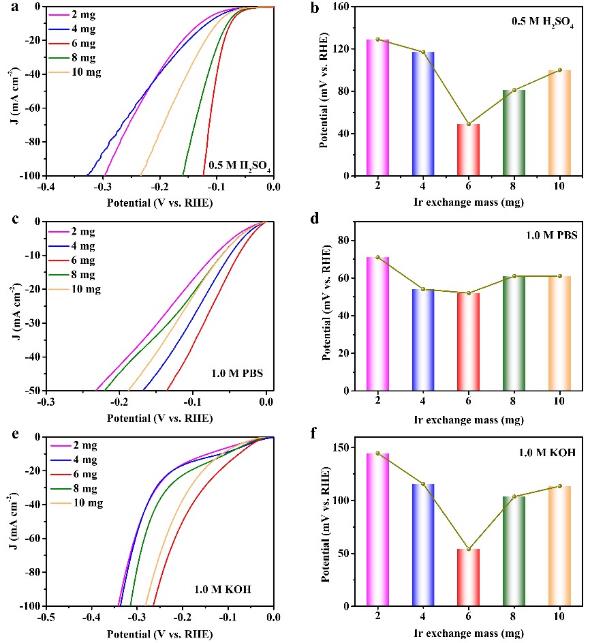


**Figure S27.** Comparison of HER performance of Ir cluster@CoO/CeO_2_ prepared by ion exchange with iridium cations of different concentrations. The HER LSV curves in (a) 0.5 M H_2_SO_4_, (c) 1.0 M PBS, and (e) 1.0 M KOH electrolytes. Comparison of HER overpotential in 10 mA cm^-2^ in (b) 0.5 M H_2_SO_4_, (d) 1.0 M PBS, and (f) 1.0 M KOH electrolytes.

**
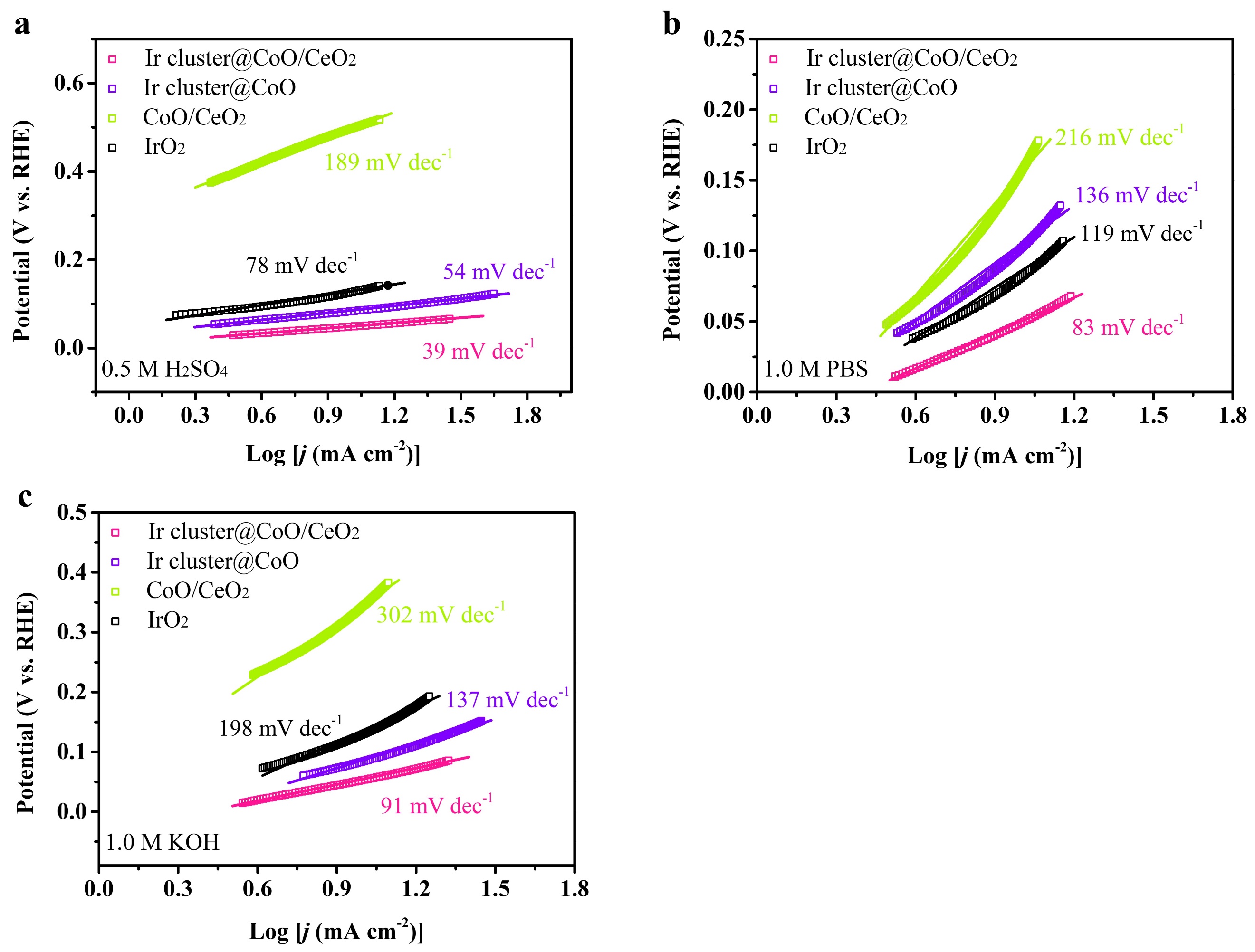
**

**Figure S28.** Tafel slope values for HER of Ir cluster@CoO/CeO_2_, Ir cluster@CoO, CoO/CeO_2_, and IrO_2_ at 10 mA cm^-2^ under 0.5 M H_2_SO_4_, 1.0 M PBS, and 1.0 M KOH, respectively.


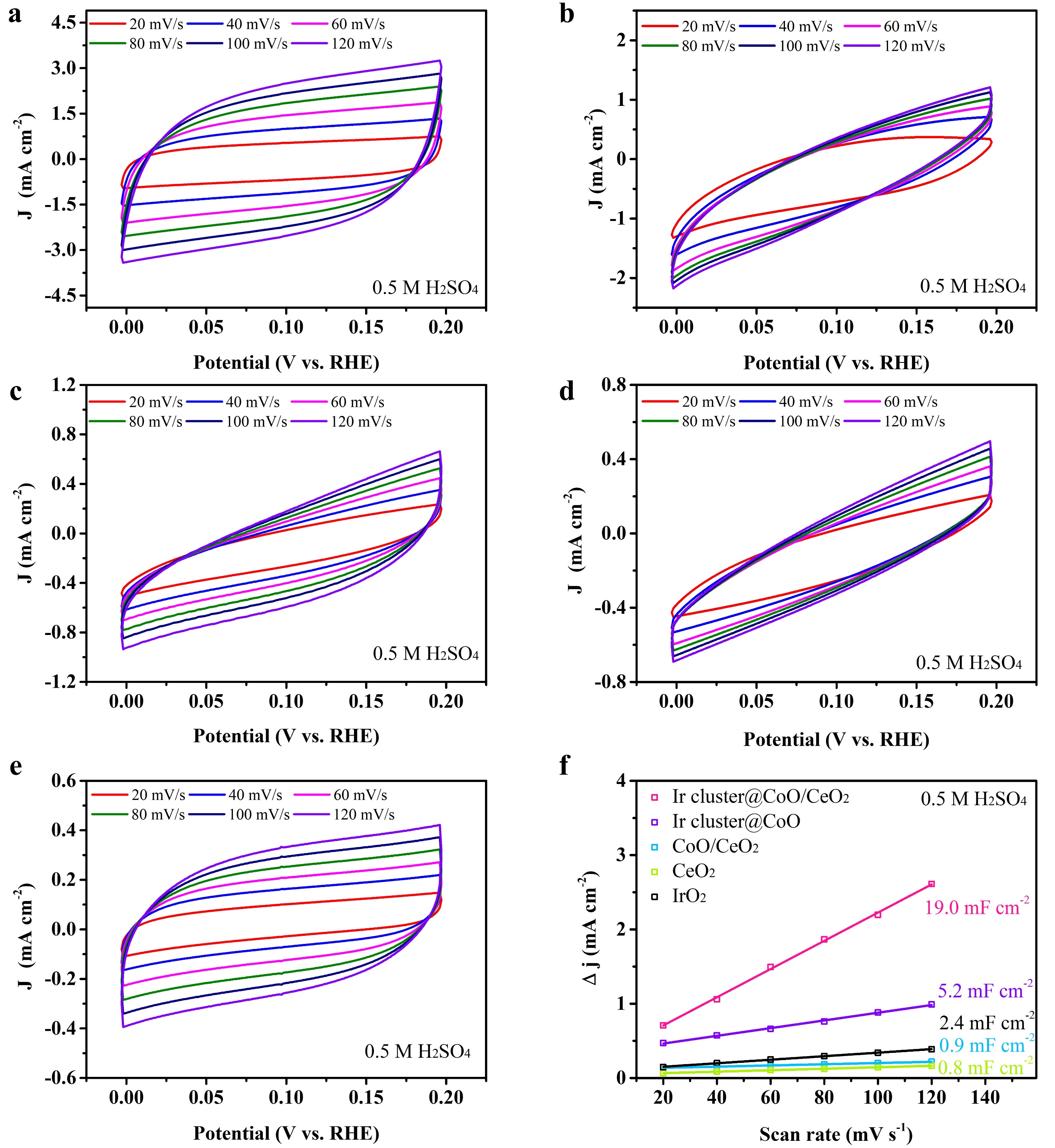


**Figure S29.** CV curves for HER of (a) Ir cluster@CoO/CeO_2_, (b) IrO_2_, (c) Ir cluster@CoO, (d) CoO/CeO_2_, and (e) IrO_2_ at increasing scan rates from 20 to 120 mV s^−1^ in 0.5 M H_2_SO_4_. (f) C_dl_ of the corresponding samples.


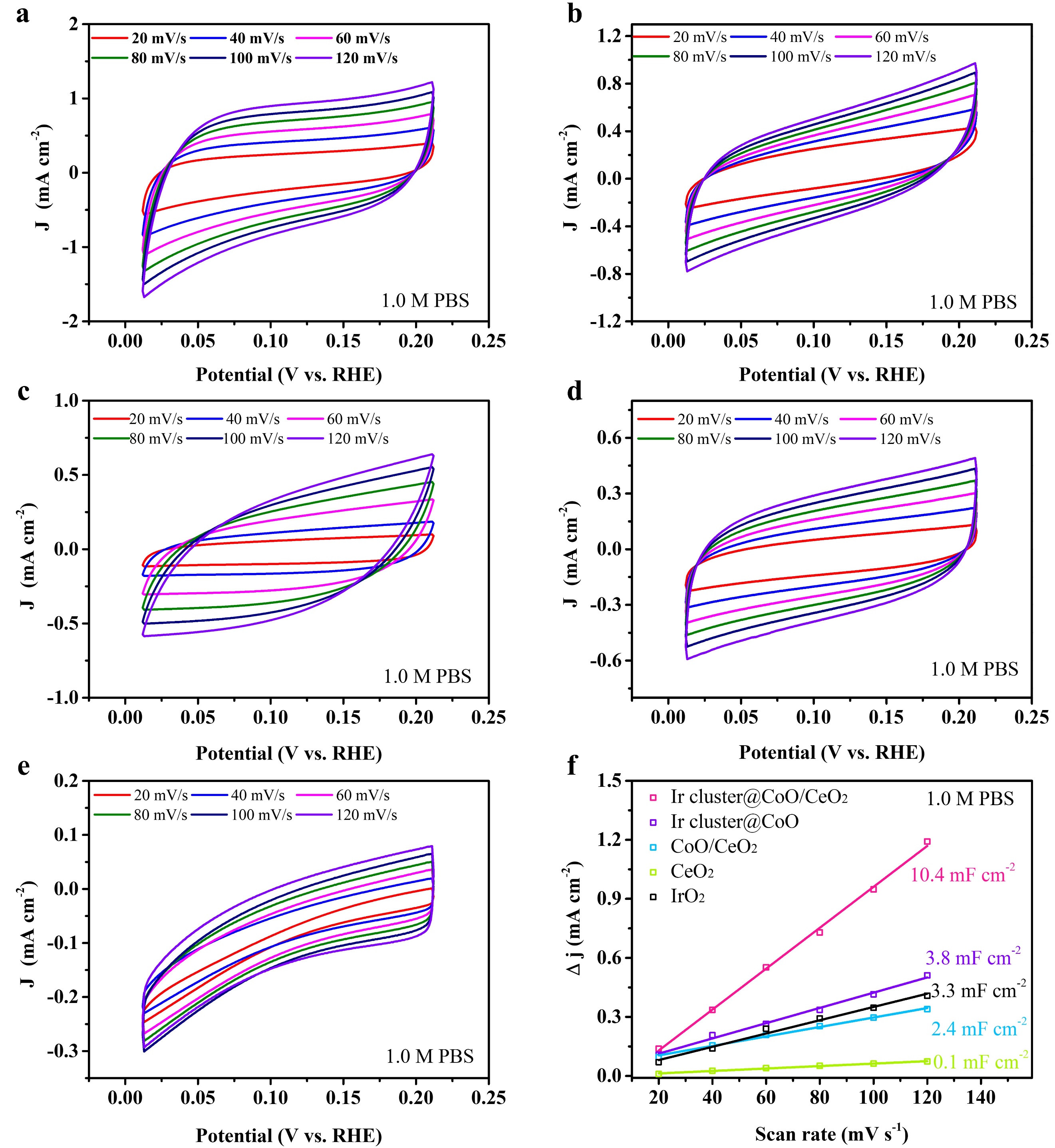


**Figure S30.** CV curves for HER of (a) Ir cluster@CoO/CeO_2_, (b) IrO_2_, (c) Ir cluster@CoO, (d) CoO/CeO_2_, and (e) IrO_2_ at increasing scan rates from 20 to 120 mV s^−1^ in 1.0 M PBS. (f) C_dl_ of the corresponding samples.


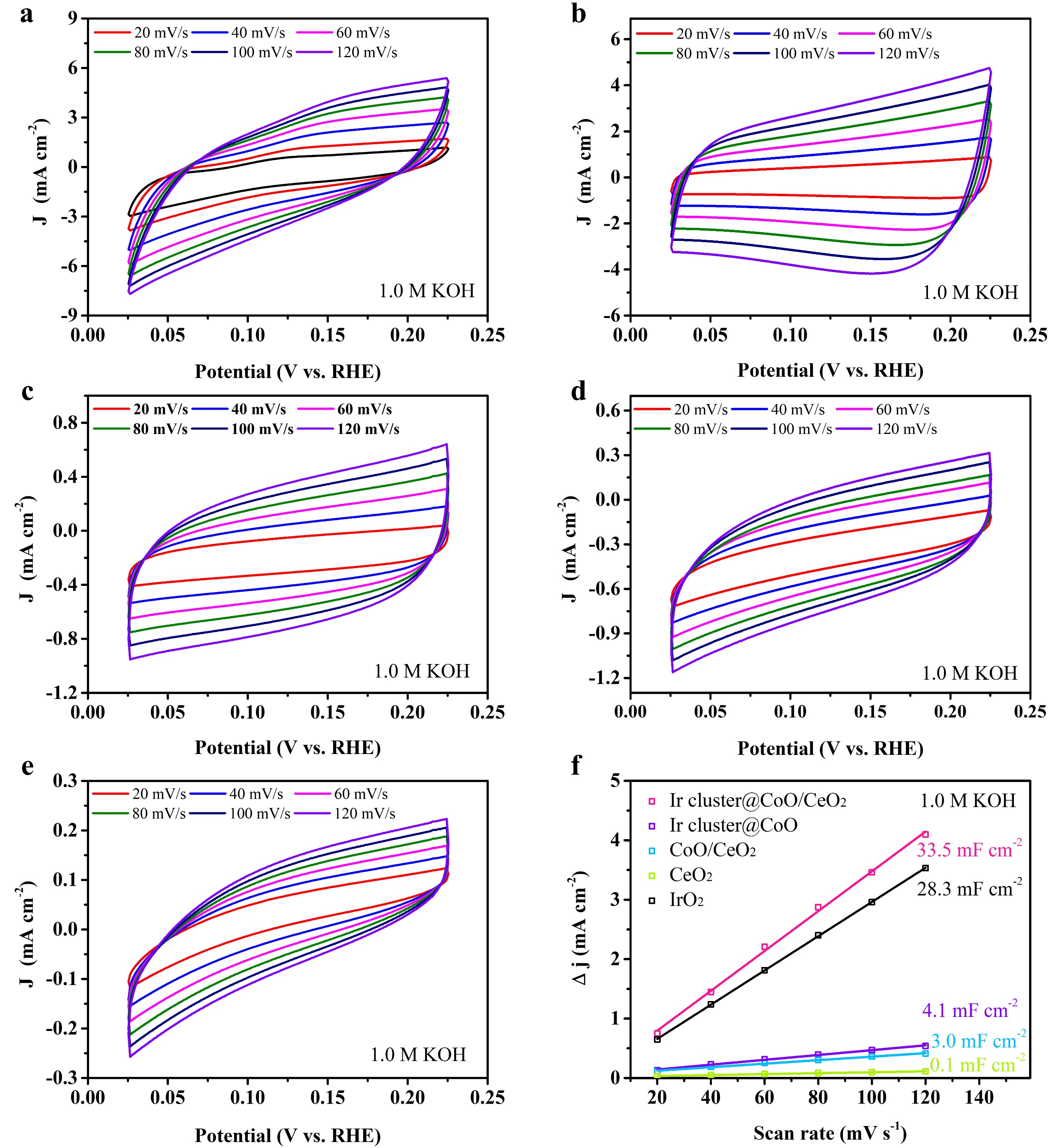


**Figure S31.** CV curves for HER of (a) Ir cluster@CoO/CeO_2_, (b) IrO_2_, (c) Ir cluster@CoO, (d) CoO/CeO_2_, and (e) IrO_2_ at increasing scan rates from 20 to 120 mV s^−1^ in 1.0 M KOH. (f) C_dl_ of the corresponding samples.


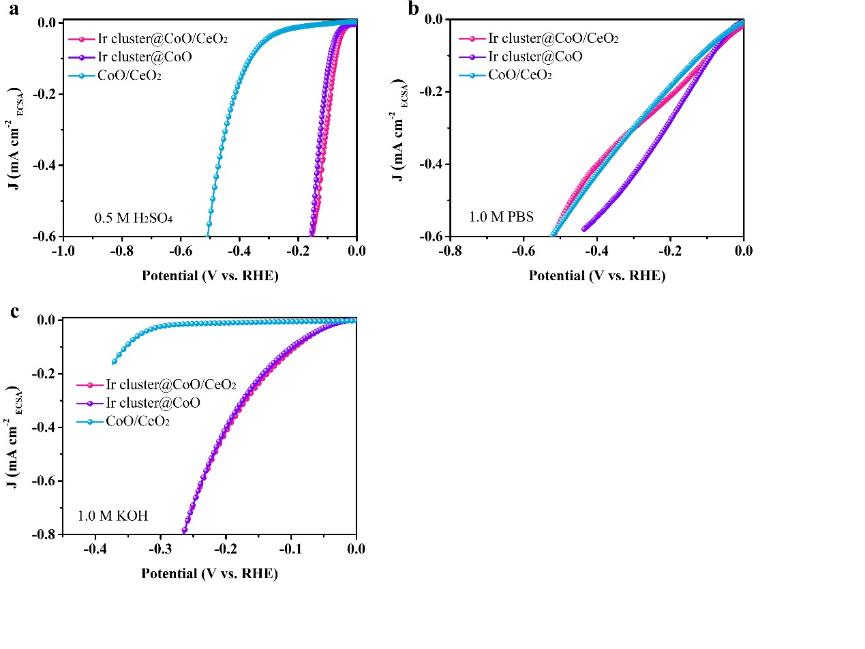


**Figure S32.** ECSA-normalized LSVs of Ir cluster@CoO/CeO_2_, Ir cluster@CoO, and CoO/CeO_2_ for HER in different electrolyte solutions.


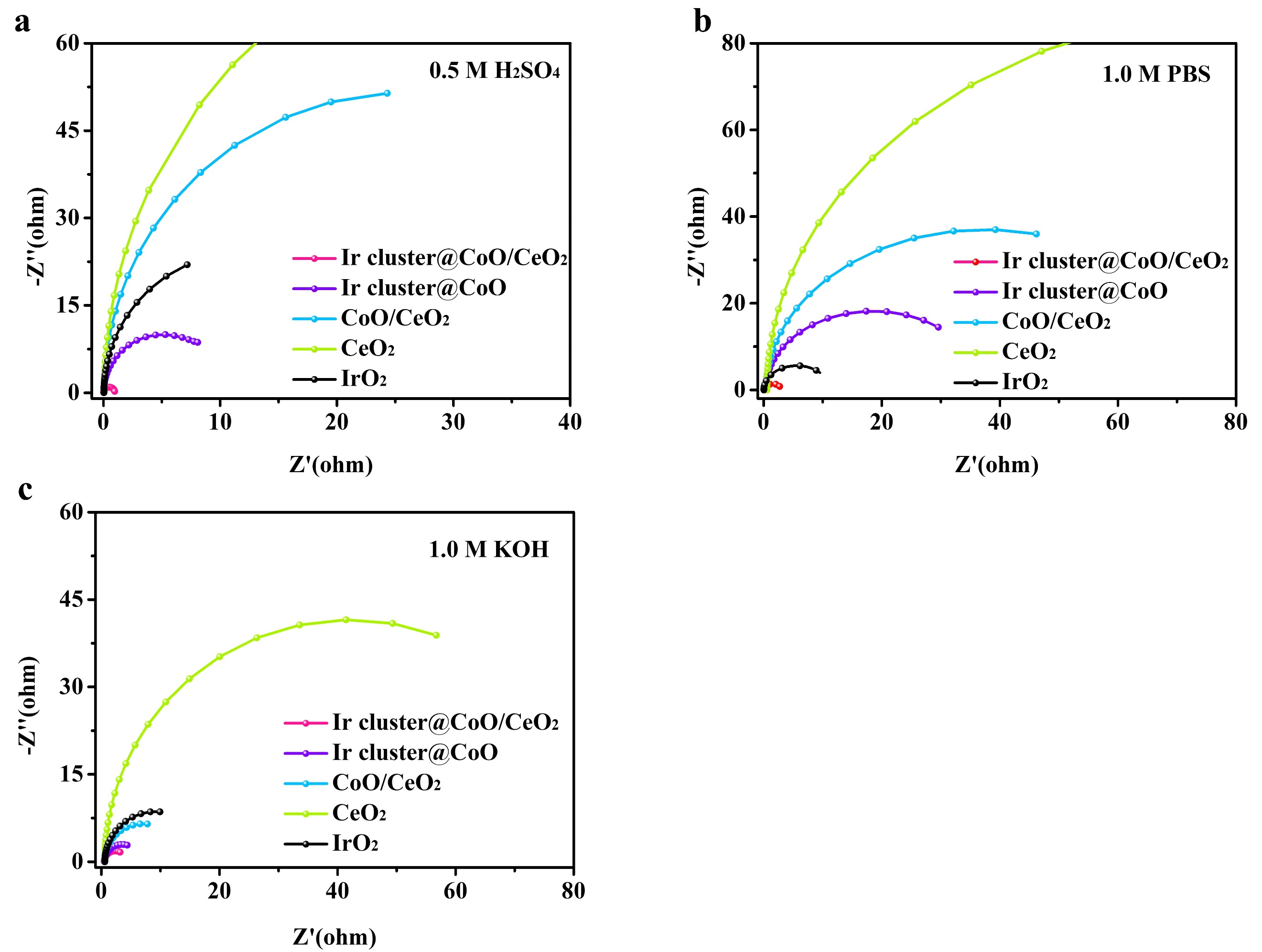


**Figure S33.** EIS curves during HER for Ir cluster@CoO/CeO_2_, Ir cluster@CoO, CoO/CeO_2_, and IrO_2_ at 10 mA cm^-2^ under (a) 0.5 M H_2_SO_4_, (b) 1.0 M PBS, and (c) 1.0 M KOH, respectively.


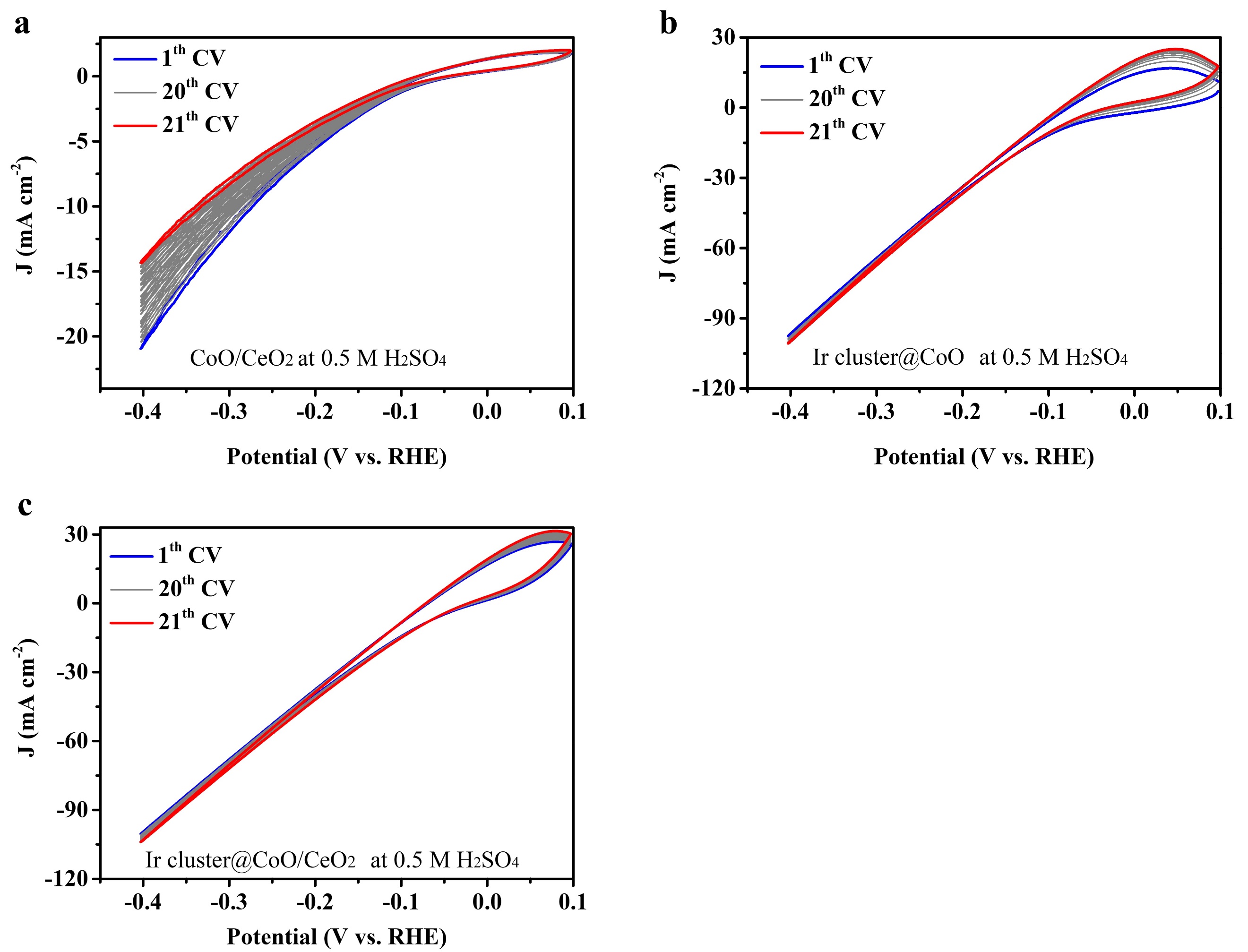


**Figure S34.** CV curves for HER of (a) CoO/CeO_2_, (b) Ir cluster@CoO, and (c) Ir cluster@CoO/CeO_2_ at 0.5 M H_2_SO_4_.


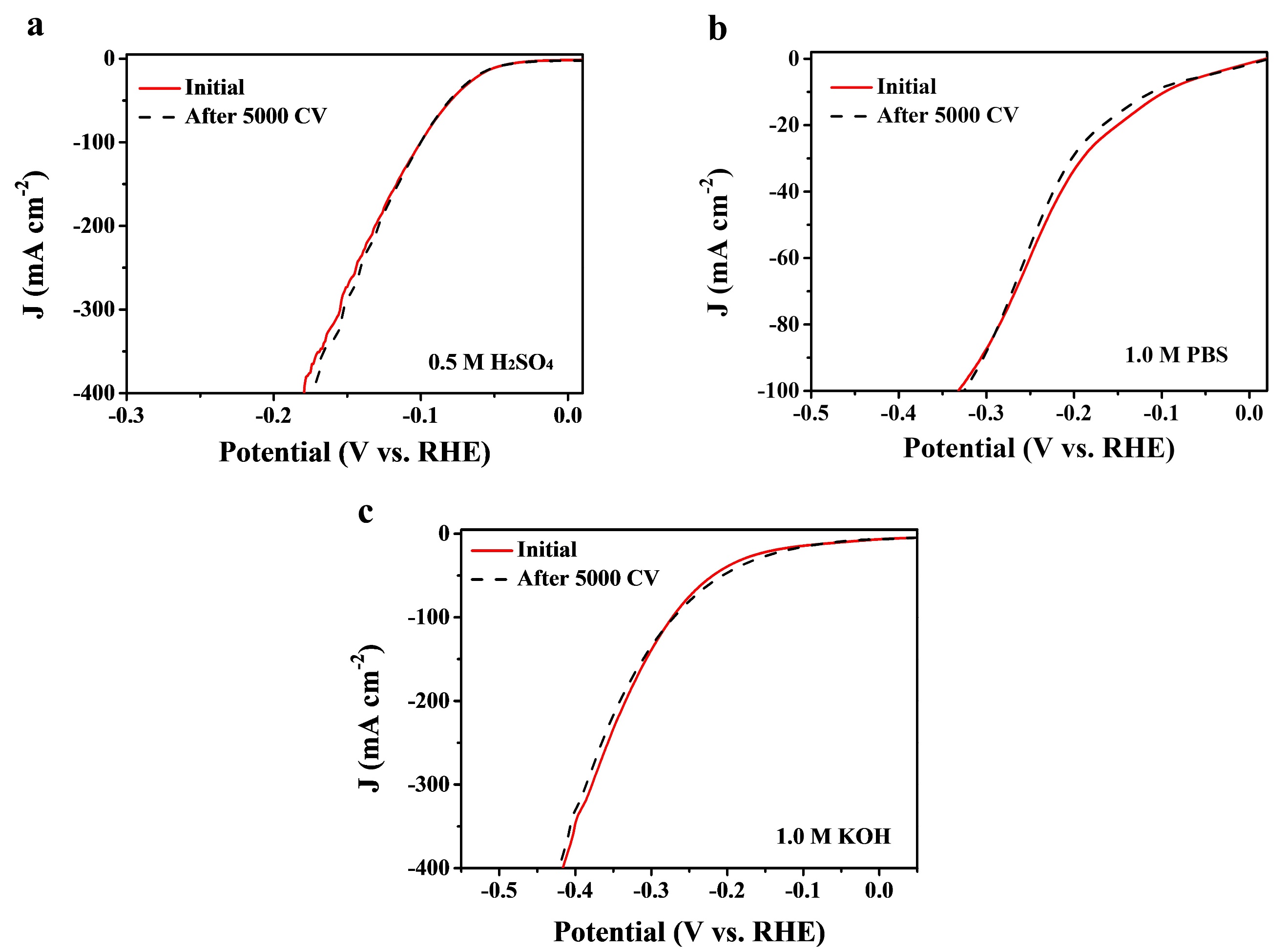


**Figure S35.** LSV cycling stability test of Ir cluster@CoO/CeO_2_ corresponding to the initial and 5000 CV cycles in (a) 0.5 M H_2_SO_4_, (b) 1.0 M PBS, and (c) 1.0 M KOH electrolytes, respectively. Under the rigorous cycling process, very small current density deviation indicates the superstable electrode material.


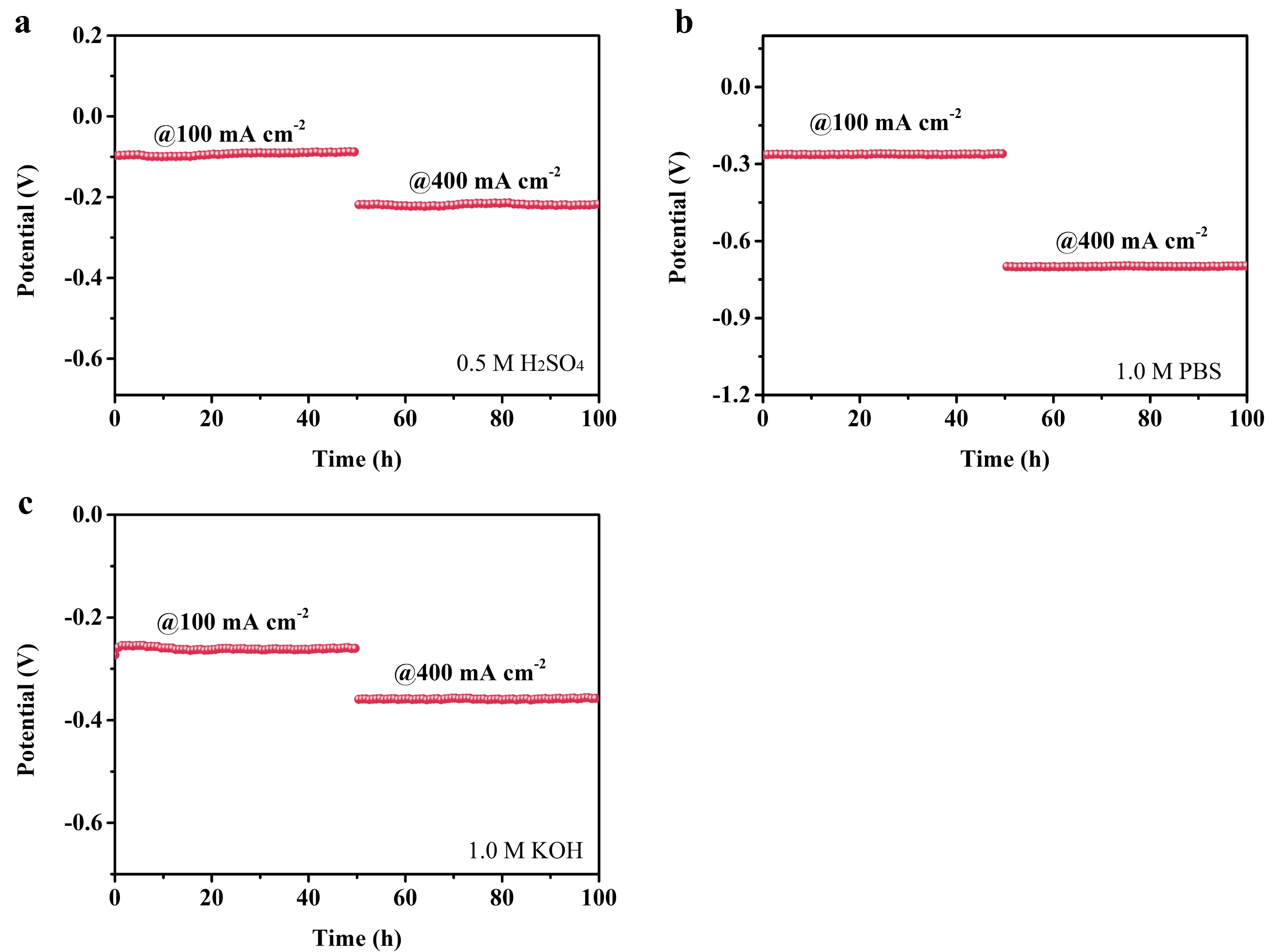


**Figure S36.** Chronopotentiometric analysis of the stability test for HER of Ir cluster@CoO/CeO_2_ at constant high current densities of 100 and 400 mA cm^-2^ in (a) 0.5 M H_2_SO_4_, (b) 1.0 M PBS, and (c) 1.0 M KOH electrolytes, respectively.


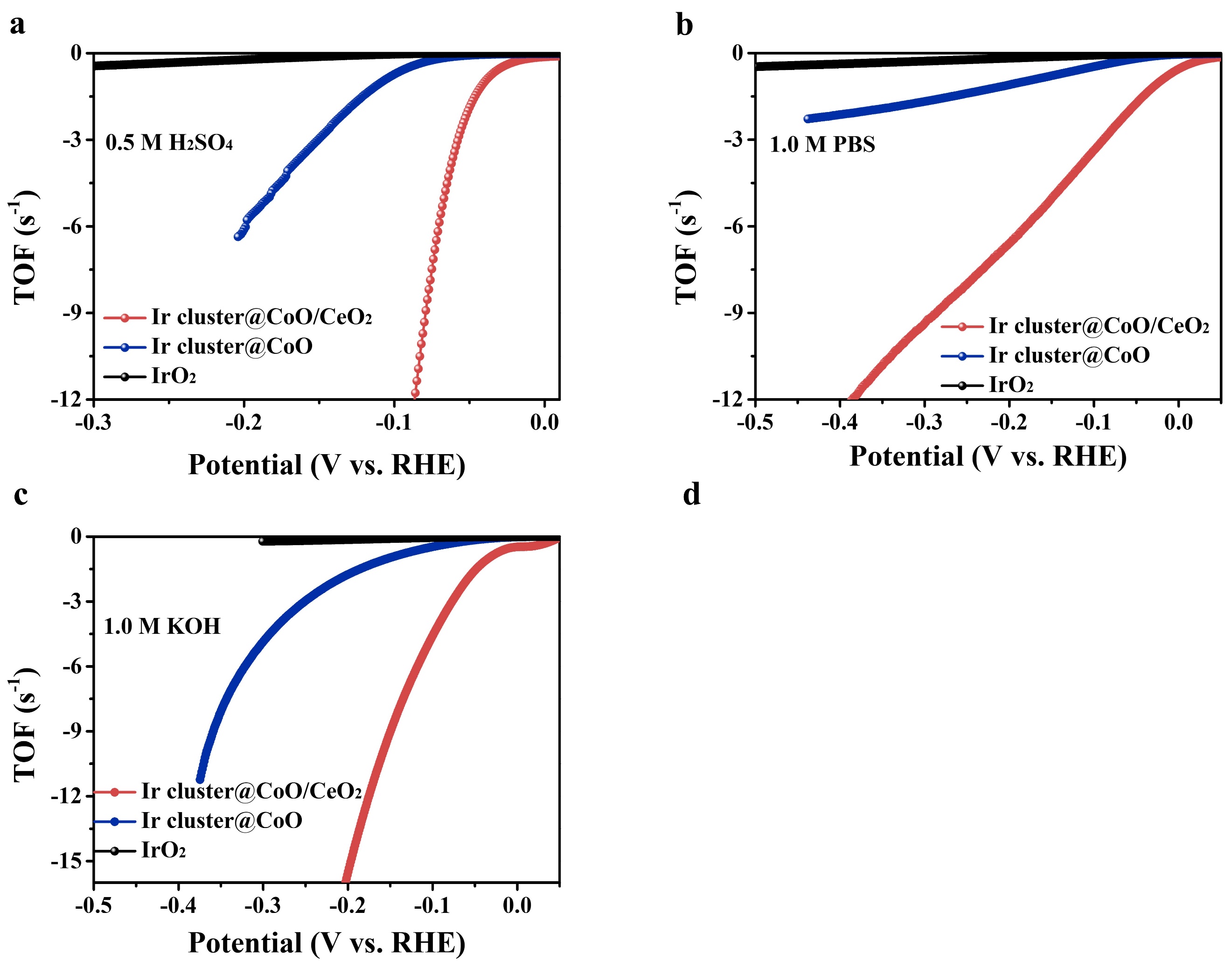


**Figure S37.** The TOF-normalized LSV curves for HER in (a) 0.5 M H_2_SO_4_, (b) 1.0 M PBS, and (c) 1.0 M KOH.


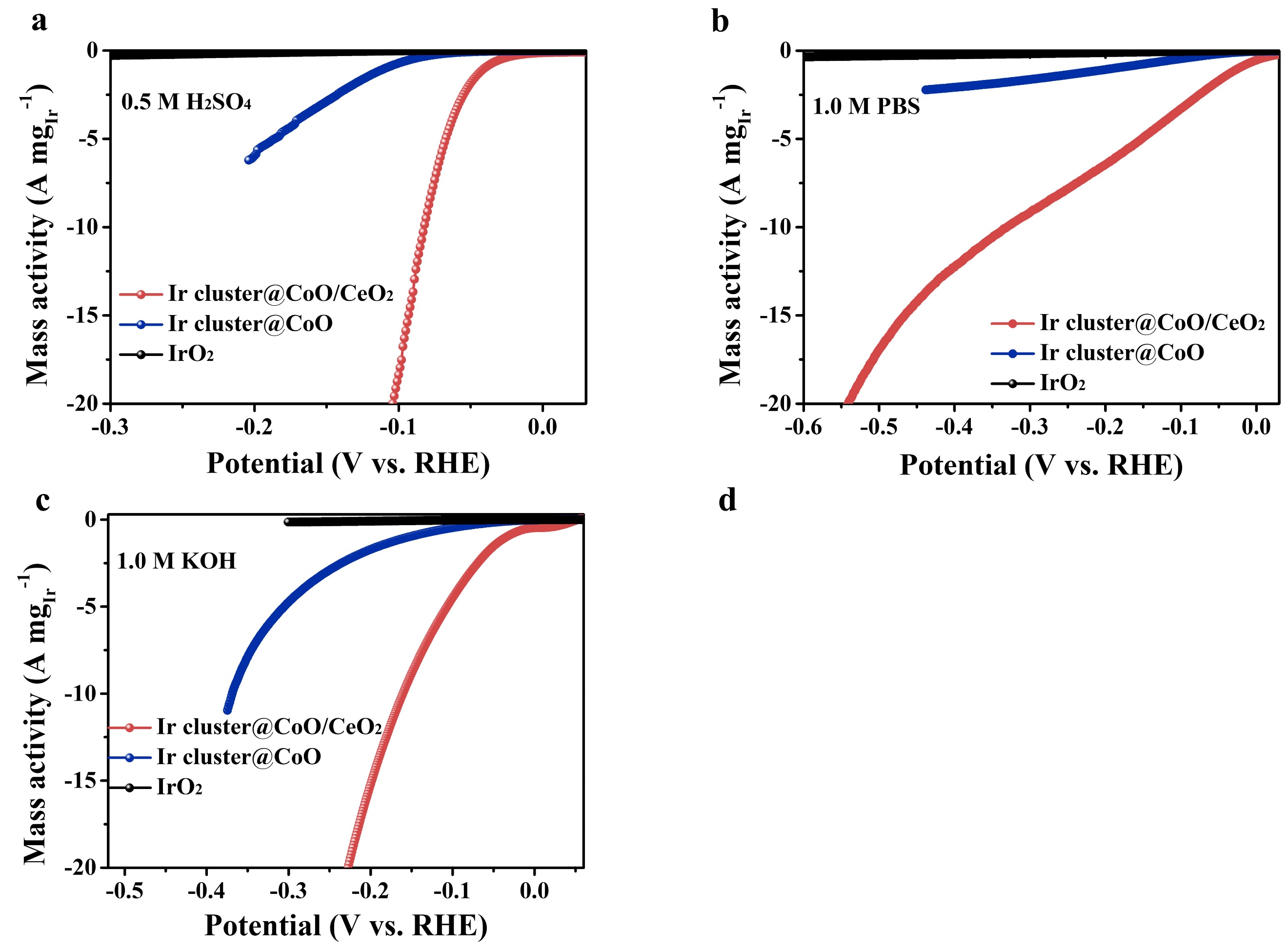


**Figure S38.** The mass-normalized LSV curves for HER in (a) 0.5 M H_2_SO_4_, (b) 1.0 M PBS, and (c) 1.0 M KOH.


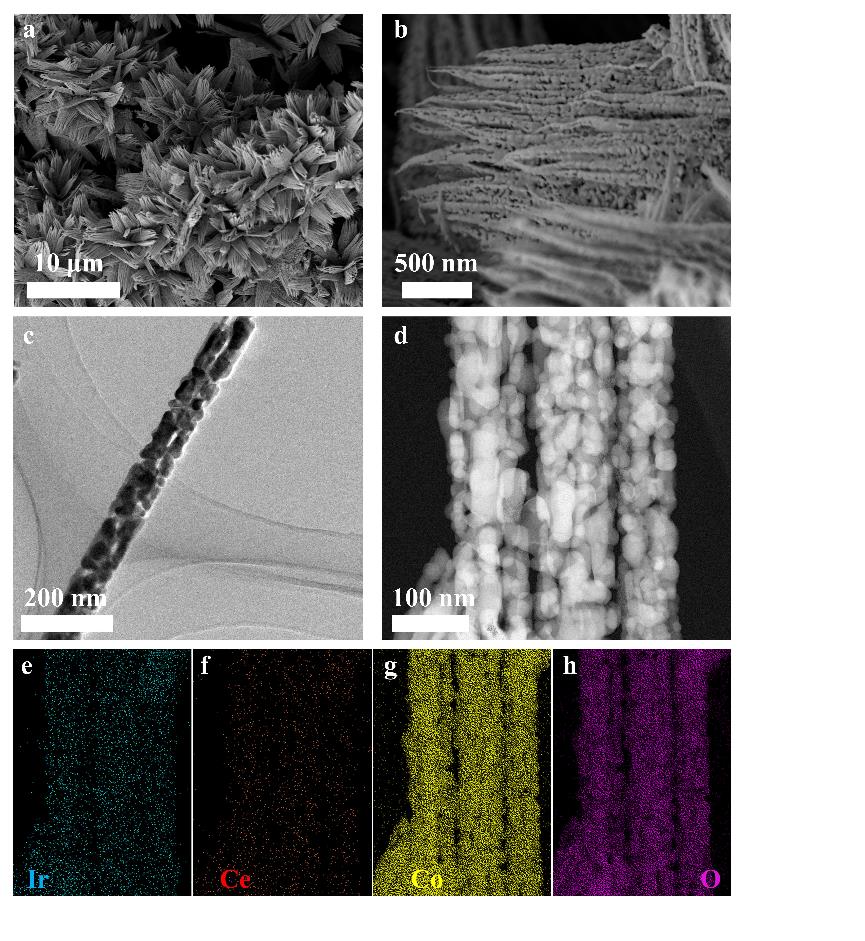


**Figure S39.** Morphology of the Ir cluster@CoO/CeO_2_ after overall water splitting test.


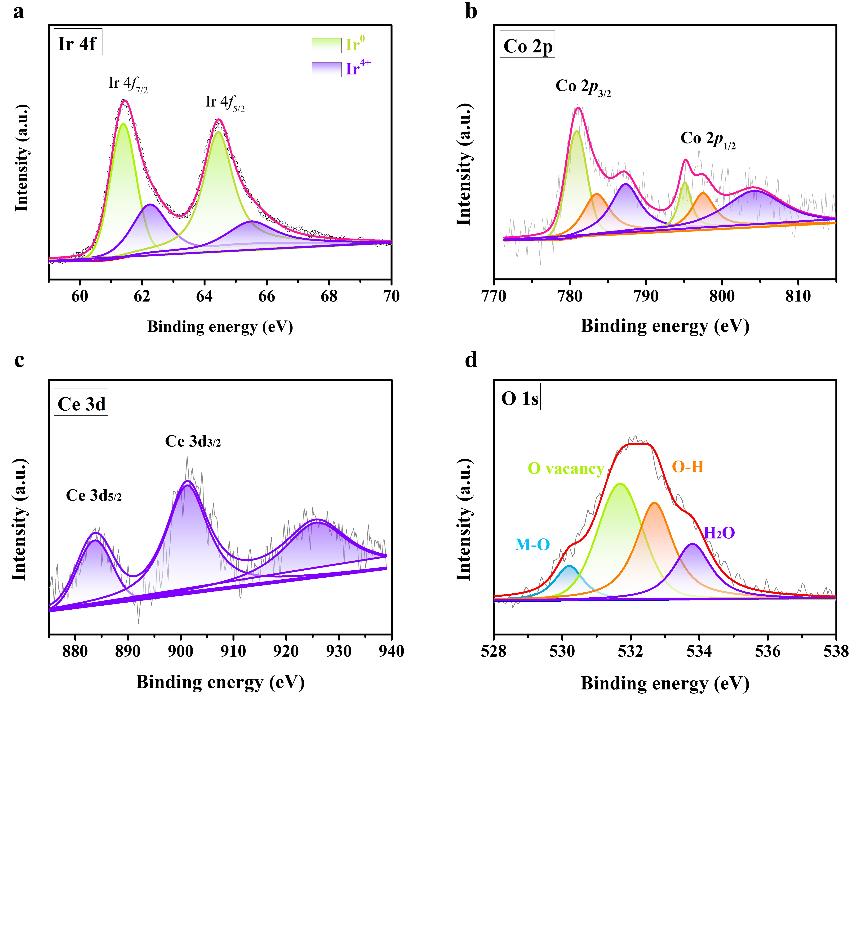


**Figure S40.** XPS of the Ir cluster@CoO/CeO_2_ after overall water splitting test.

**
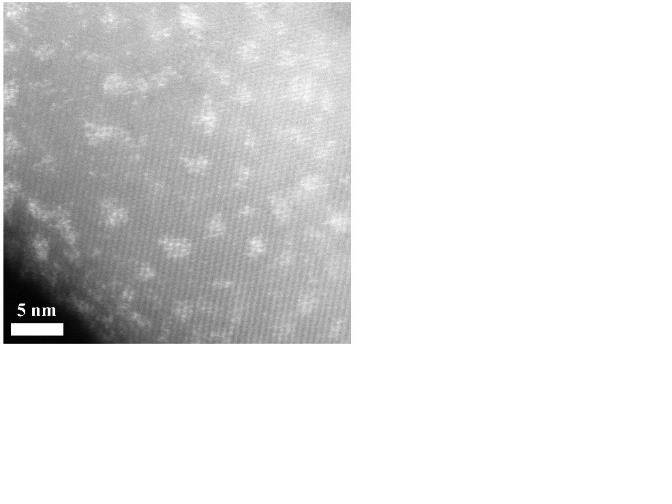
**

**Figure S41.** AC HAADF-STEM image of Ir cluster@CoO/CeO_2_ after stability test.


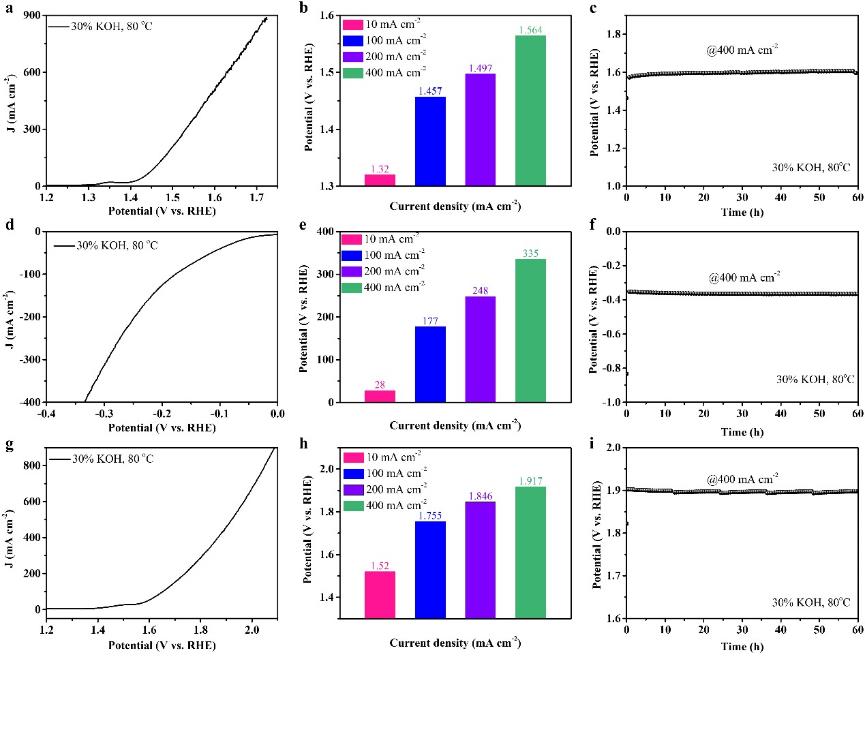


**Figure S42.** The catalytic performance of Ir cluster@CoO/CeO_2_ under the industrial electrolytic water system. (a, d, and g) LSV curves of the Ir cluster@CoO/CeO_2_ electrode for OER, HER, and overall water splitting in 30% KOH at 80 ^o^C. (b, e, and h) Potentials for OER, HER, and overall water splitting on the Ir cluster@CoO/CeO_2_ electrode at current densities of 10, 100, 200, and 400 mA cm^-2^ in 30% KOH at 80 ^o^C. The results were carried out with 90% iR-compensation. (c, f, and i) Chronpotentiometric curve at 400 mA cm^-2^ for the Ir cluster@CoO/CeO_2_ electrode towards OER, HER, and overall water splitting in 30% KOH at 80 ^o^C.


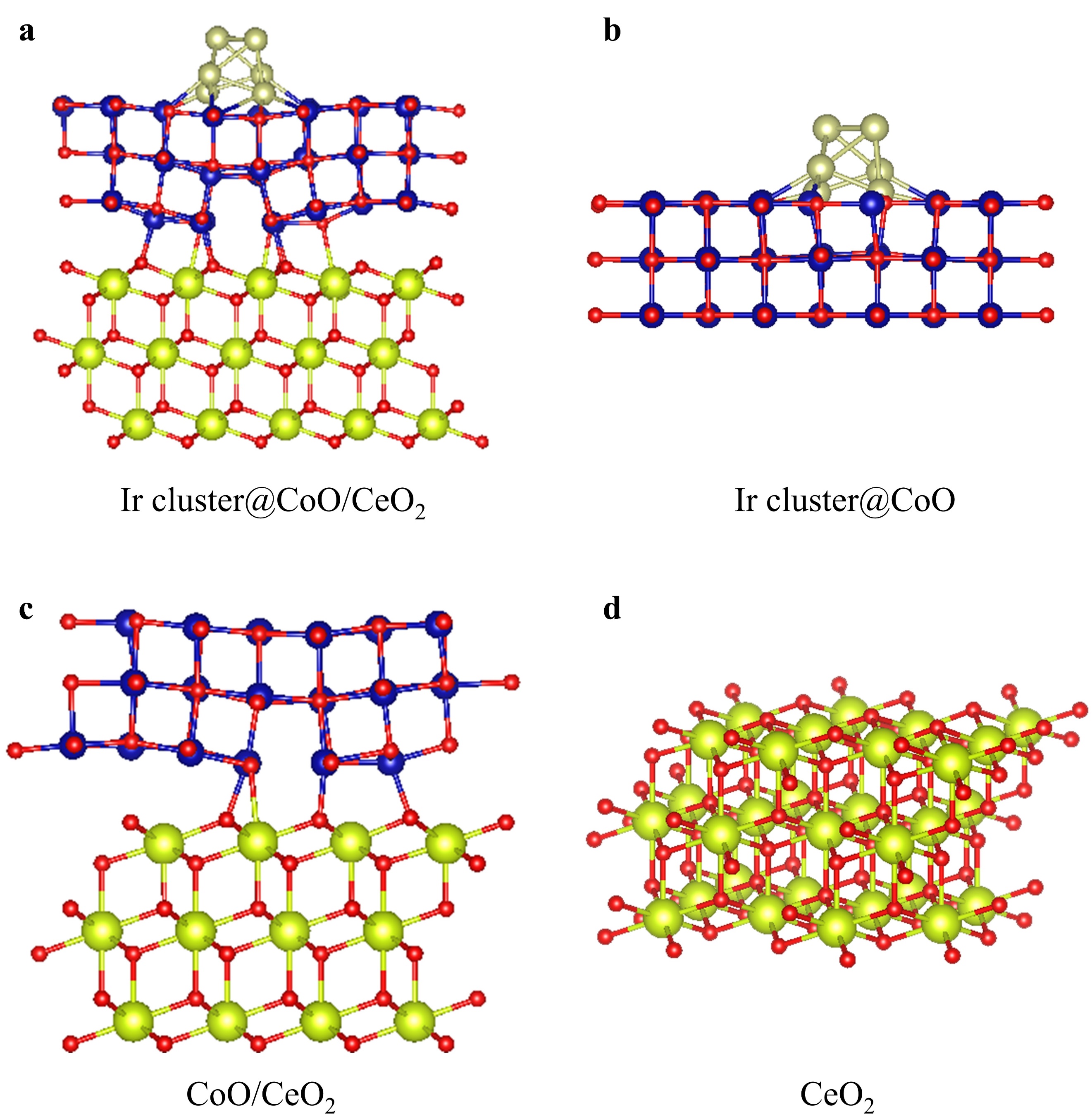


**Figure S43**. DFT models of (a) Ir cluster@CoO/CeO_2_, (b) Ir cluster@CoO, (c) CoO/CeO_2_, and (d) CeO_2_.


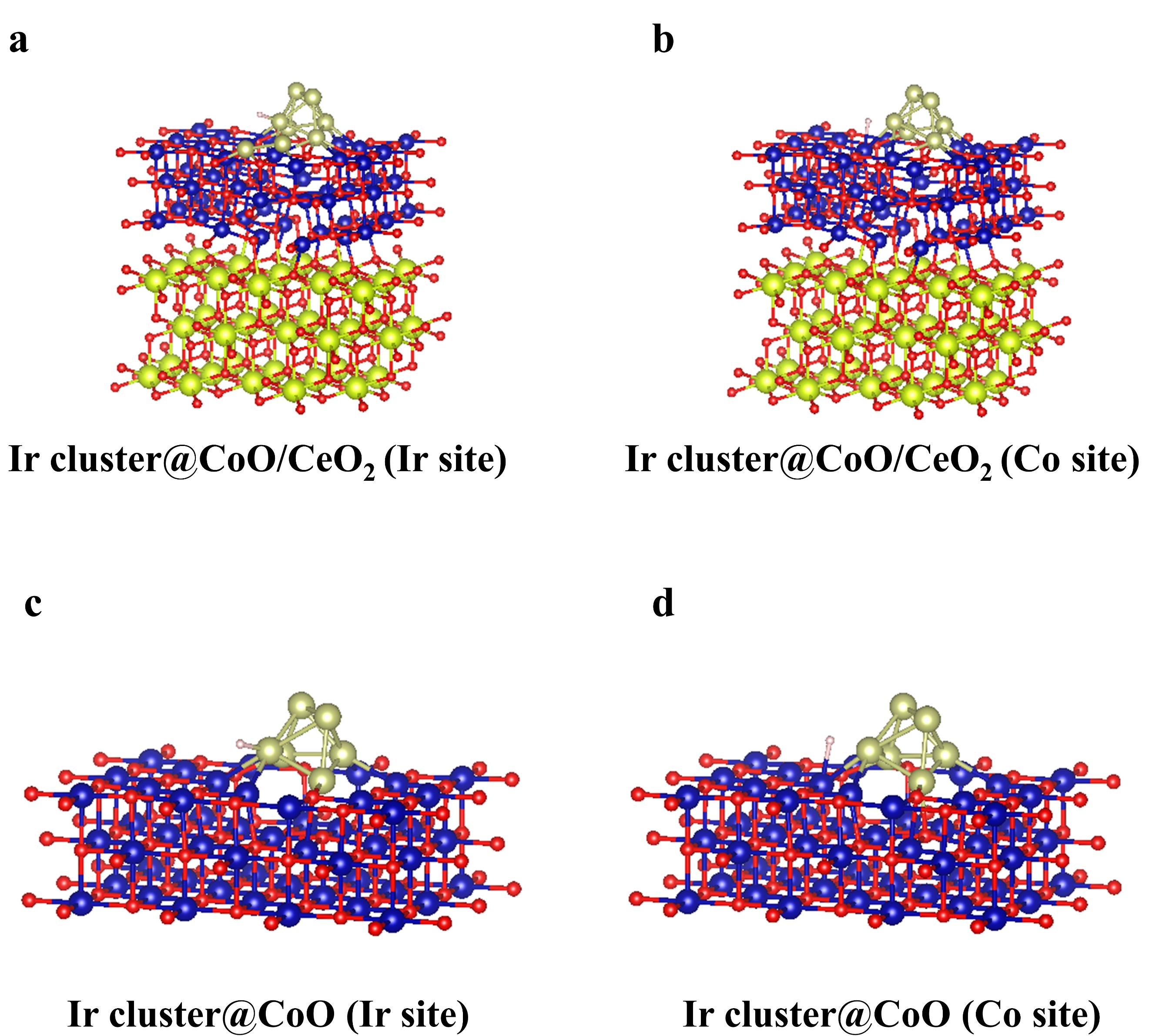


**Figure S44**. DFT models of *H on (a) Ir cluster@CoO/CeO_2_ (Ir site), (b) Ir cluster@CoO/CeO_2_ (Co site), (c) Ir cluster@CoO (Ir site), and (d) Ir cluster@CoO (Co site).

**
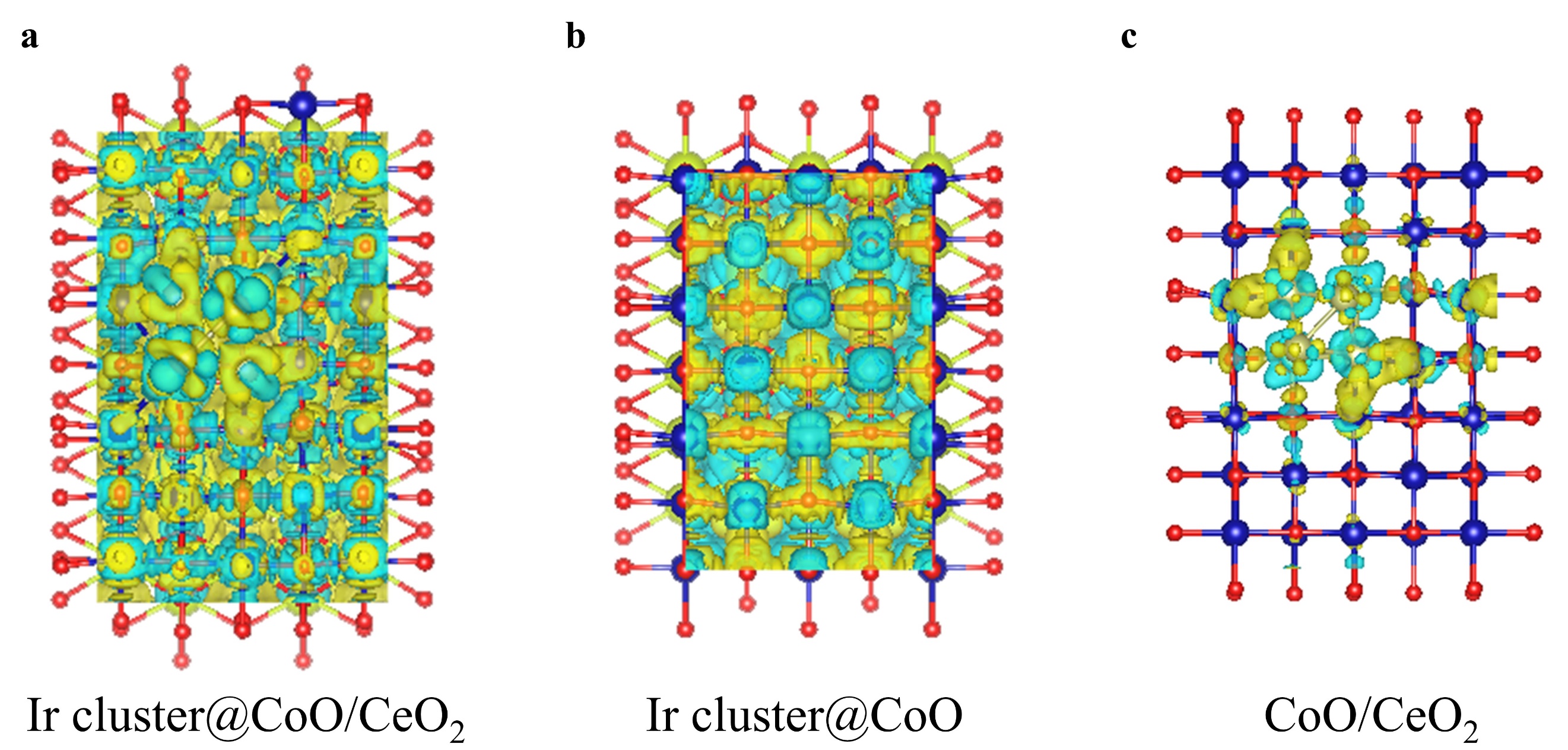
**

**Figure S45**. Charge-density difference plot of Ir cluster@CoO/CeO_2_, Ir cluster@CoO, CoO/CeO_2_ in top view. Color code: Co (blue), Ir (gold), Ce (yellow), and O (red). The yellow (blue) shadows represent the electron accumulation (depletion).

**
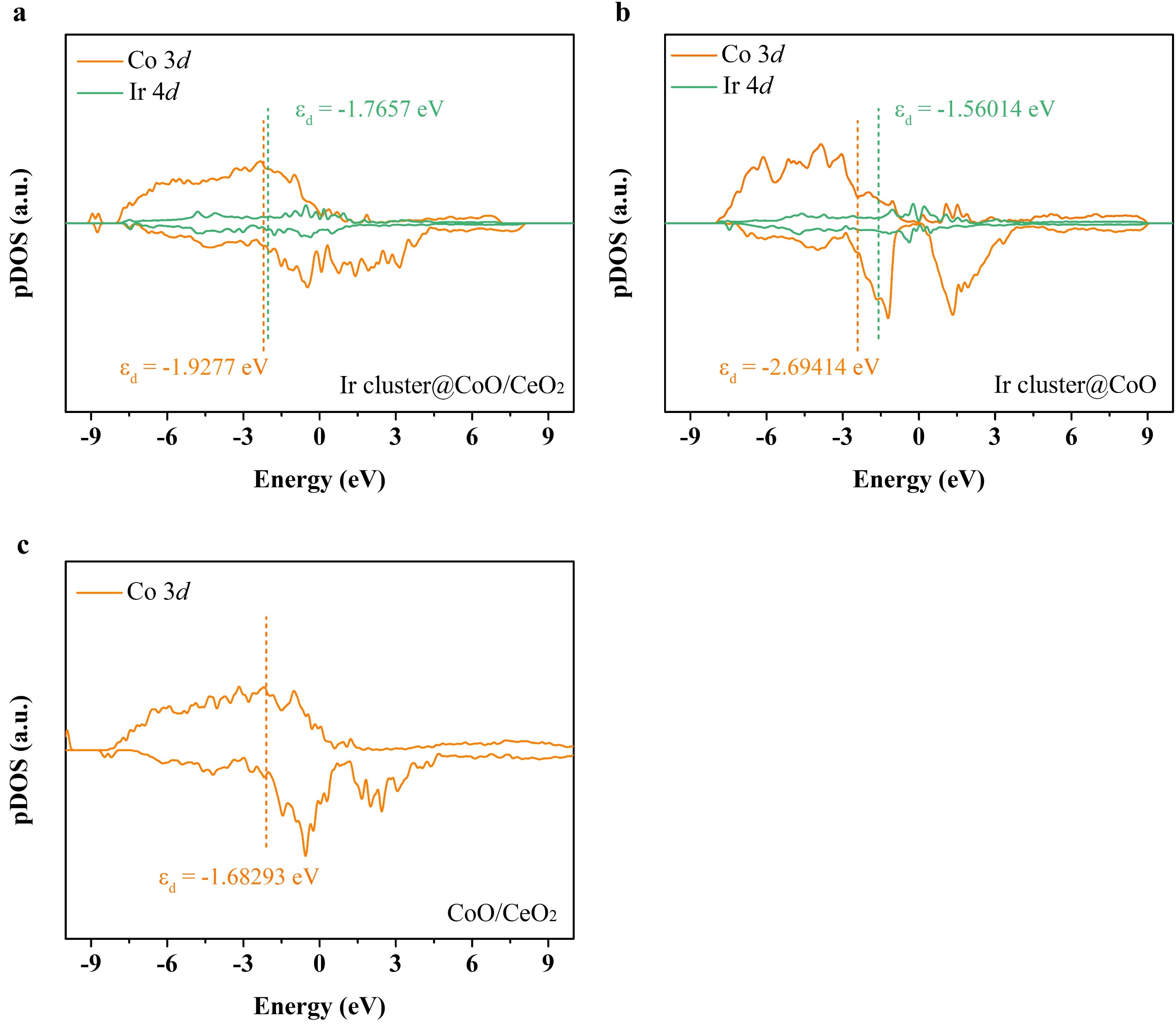
**

**Figure S46**. d-band center of Ir atom and Co atom for (a) Ir cluster@CoO/CeO_2_, (b) Ir cluster@CoO, and (c) CoO/CeO_2_.


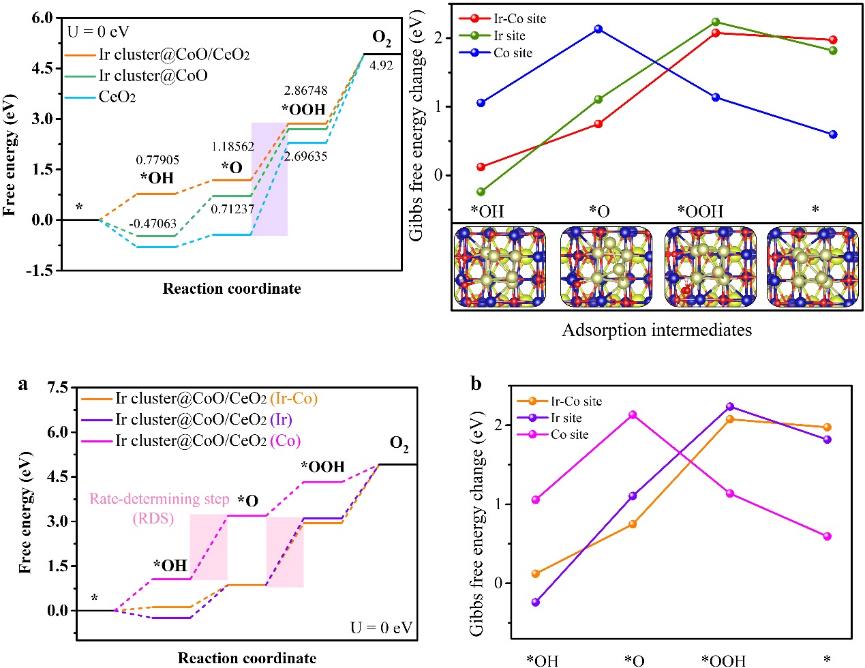


**Figure S47**. (h) Standard free energy diagrams of the OER pathway on different activities of Ir cluster@CoO/CeO_2_ at U = 0 V. (b) At U = 0 V, a comparison of Gibbs free energy changes of OER intermediates on different activities of Ir cluster@CoO/CeO_2_.

**
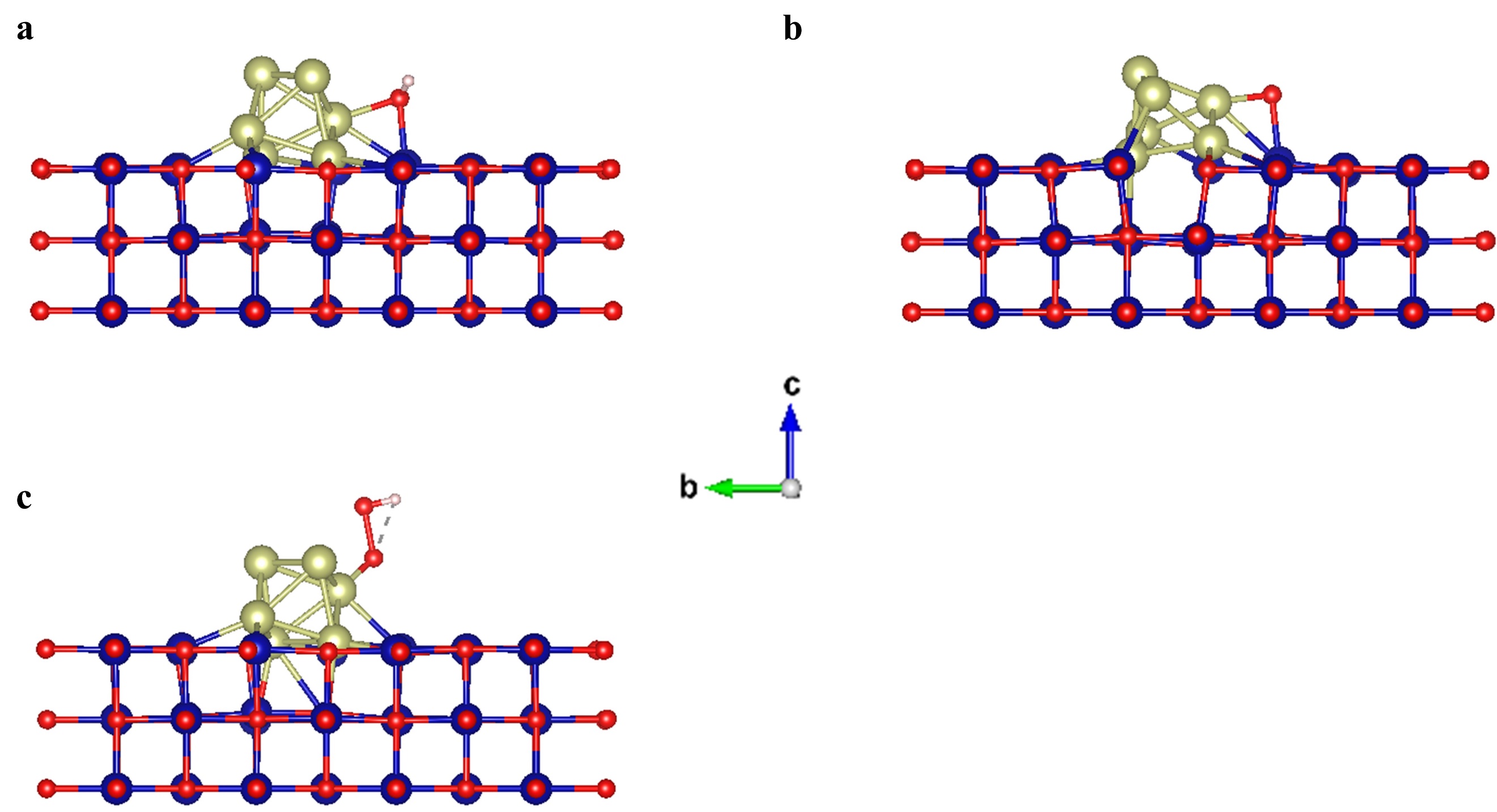
**

**Figure S48**. Local structural configurations of *OH, *O, and *OOH intermediates on the Ir cluster@CoO in the AEM pathway.


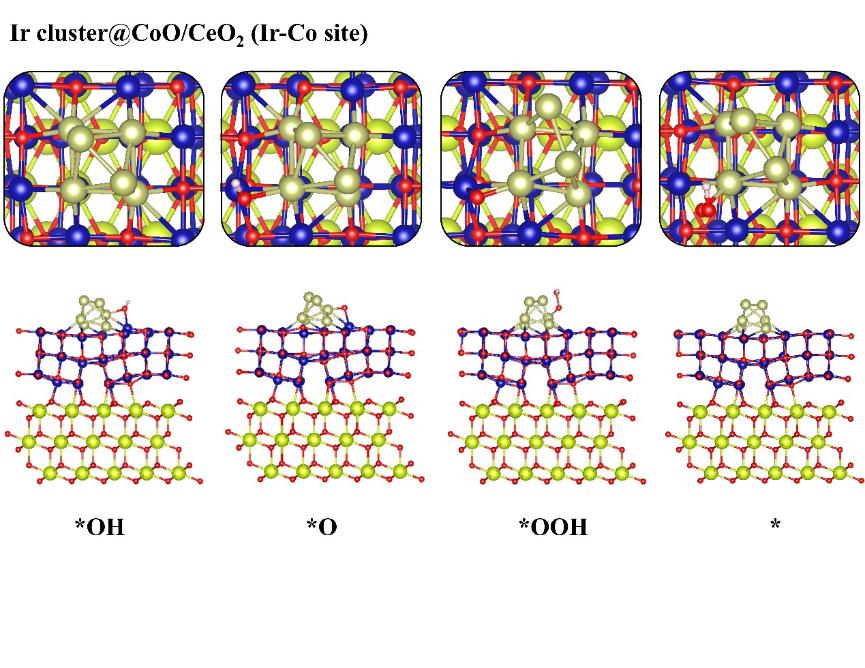


**Figure S49**. Optimized configurations of OER intermediates on the Ir-Co site of Ir cluster@CoO/CeO_2_. The golden, blue, red, yellow, and white ball marks Ir, Co, O, Ce, and H atoms, respectively.


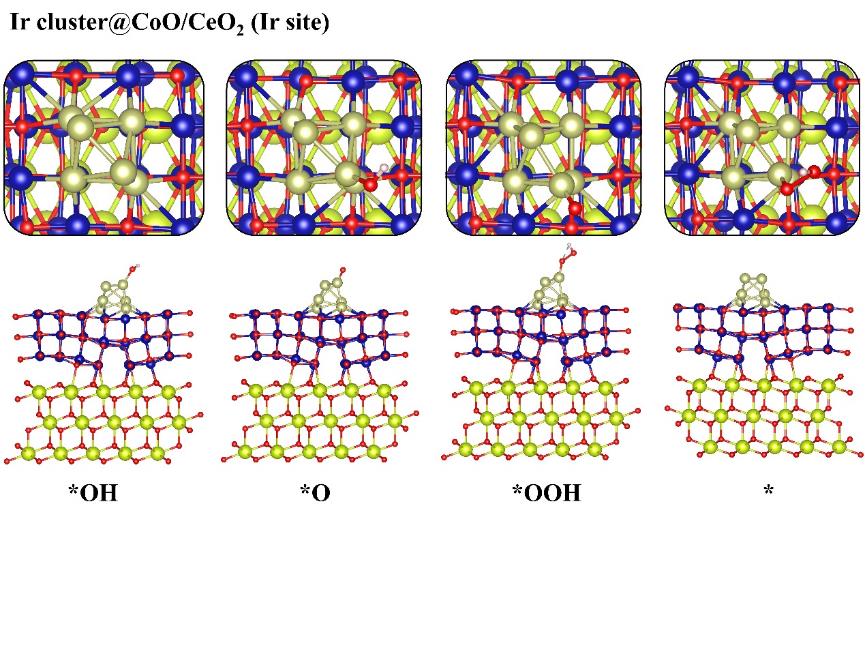


**Figure S50**. Optimized configurations of OER intermediates on the Ir site of Ir cluster@CoO/CeO_2_. The golden, blue, red, yellow, and white ball marks Ir, Co, O, Ce, and H atoms, respectively.


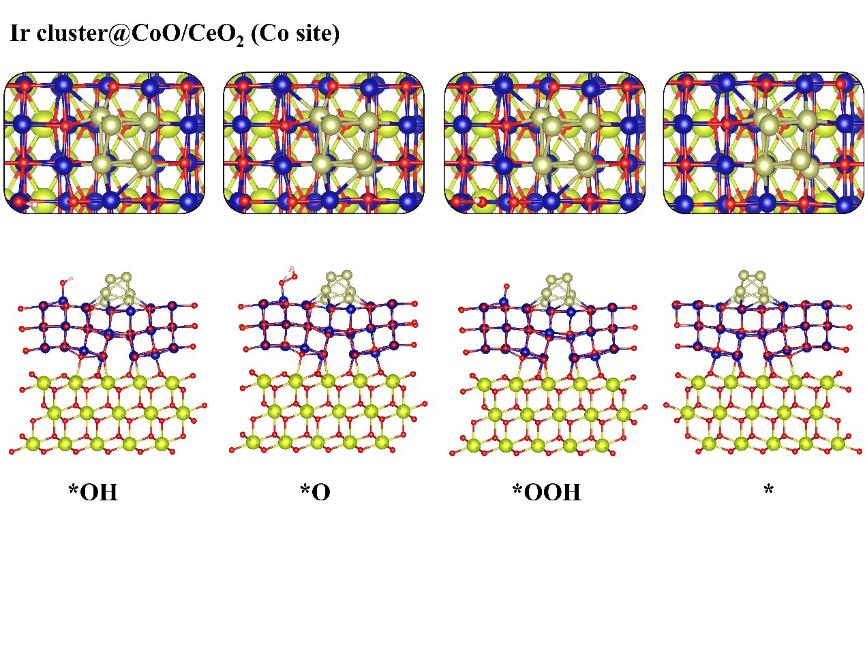


**Figure S51**. Optimized configurations of OER intermediates on the Co site of Ir cluster@CoO/CeO_2_. The golden, blue, red, yellow, and white ball marks Ir, Co, O, Ce, and H atoms, respectively.

**Table S1.** XPS fitting parameters for the Co peak area of the as-prepared catalysts.

| Samples | Peak area | | | | Co^2+^/Co^3+^ ratio |
| --- | --- | --- | --- | --- | --- |
|  | Co^3+^ | | Co^2+^ | |  |
|  | 2p_3/2_ | 2p_1/2_ | 2p_3/2_ | 2p_1/2_ |  |
| Ir cluster@CoO/CeO_2_ | 43123.43 | 13000.0 | 30364.2 | 17997.57 | 0.86 |
| Ir cluster@CoO | 9948.78 | 3768.09 | 7628.41 | 4968.09 | 0.92 |

**Table S2.** OER performance comparison of as-prepared Ir cluster@CoO/CeO_2_ with other typical electrocatalysts under acidic medium.

| **Catalyst** | Activity  (η_10_, mV) | Tafel slope  (mV dec^-1^) | C_dl_  (mF cm^-2^) | TOF^a^  (s^-1^) | MA^b^  (A mg_Ir_^-1^) | Stability  (h) | Ref. |
| --- | --- | --- | --- | --- | --- | --- | --- |
| Ir cluster@CoO/CeO_2_ | 215 | 97 | 19.6 | 17.9 | 17.5 | 300 | This work |
| Ir-SA@Fe@NCNT | 250 | 58.2 | 14.2 | 4.4 | 13.7 | 12 | ^[1]^ |
| AD-HN-Ir | 216 | 39 | 5.2 | 9.25 | 10.9 | 100 | ^[2]^ |
| Ir-Co_3_O_4_ | 236 | 52.6 | 38.54 | 1.665 | 3.343 | 30 | ^[3]^ |
| ZnNiCoIrMn | 237 | 46 | 19.45 | 7.53 | 0.61 | 100 | ^[4]^ |
| Ir-NiCo_2_O_4_ NSs | 240 | 60 | 108 | 1.13 | 10 | 70 | ^[5]^ |

a TOF-turnover frequency value,

b MA-mass activity.

**Table S3.** OER performance comparison of as-prepared Ir cluster@CoO/CeO_2_ with other typical electrocatalysts under neutral medium.

| **Catalyst** | Activity  (η_10_, mV) | Tafel slope  (mV dec^-1^) | C_dl_  (mF cm^-2^) | TOF  (s^-1^) | R_ct_  (Ω) | Stability  (h) | Ref. |
| --- | --- | --- | --- | --- | --- | --- | --- |
| Ir cluster@CoO/CeO_2_ | 390 | 87 | 16.1 | 0.24 | 3.9 | 300 | This work |
| Ni_6_Fe_1_Cu_1_ | 358 | 164 | 0.503 | 0.12 | 162.5 | 100 | ^[6]^ |
| NiS_x_-24h | 173 | 209.5 | 7.52 | 72 | 3.49 | 12 | ^[7]^ |
| Co_3-x_Pd_x_O_4_ | 370 | 96 | 14.12 | 0.18 | 15.6 | 100 | ^[8]^ |
| Ni_0.1_Co_0.9_P | 520 | 133 | 11.3 | 0.12 | 10 | 20 | ^[9]^ |

**Table S4.** OER performance comparison of as-prepared Ir cluster@CoO/CeO_2_ with other typical electrocatalysts under alkaline medium.

| **Catalyst** | Activity  (η_10_, mV) | Tafel slope  (mV dec^-1^) | C_dl_  (mF cm^-2^) | TOF  (s^-1^) | R_ct_  (Ω) | Stability  (h) | Ref. |
| --- | --- | --- | --- | --- | --- | --- | --- |
| Ir cluster@CoO/CeO_2_ | 243 | 61 | 28.1 | 14.1 | 7 | 300 | This work |
| Ir_1_/Ni LDH-T | 228 | 41 | 6.2 | 7.6 | 8.2 | 60 | ^[10]^ |
| Ir_1_/TO-CoOOH | 200 | 32 | 0.6 | 0.144 | 12 | 20 | ^[11]^ |
| DD-Ni-NDA | 260 | 50 | 5.83 | 0.0089 | 10 | 10 | ^[12]^ |
| VMoON@NC | 212 | 19.4 | 151.71 | 0.0188 | 9.89 | 71 | ^[13]^ |
| Ce@NiFe-LDH | 205 | 40.1 | 1.52 | 0.084 | 0.96 | 48 | ^[14]^ |
| Pd_2_RuO_x_-0.5 h | 225 | 41.9 | 22.9 | 2.52 | 3.14 | 24 | ^[15]^ |
| IrO_2_/V_2_O_5_ | 283 | 34 | 0.637 | 0.445 | 56 | 20 | ^[16]^ |

**Table S5.** HER performance comparison of as-prepared Ir cluster@CoO/CeO_2_ with other typical electrocatalysts under acidic medium.

| **Catalyst** | Activity  (η_10_, mV) | Tafel slope  (mV dec^-1^) | C_dl_  (mF cm^-2^) | TOF  (s^-1^) | MA  (A mg_mental_^-1^) | Stability  (h) | Ref. |
| --- | --- | --- | --- | --- | --- | --- | --- |
| Ir cluster  @CoO/CeO_2_ | 49 | 39 | 19 | 18.8 | 18.4 | 300 | This work |
| ZnNiCoIrMn | 22 | 30.6 | 30.19 | 6.4 | 0.68 | 100 | ^[4]^ |
| Ir_25_Ni_33_Ta_42_/Si | 99 | 35 | 0.05 | 1.76 | 1 | 10 | ^[17]^ |
| Pt_3_Fe/NMCS-A | 13 | 21 | 27.34 | 1.95 | 1.94 | 10 | ^[18]^ |
| Rh(OH)_3_/CoP | 12 | 25 | 230 | 15 | 3.5 | 70 | ^[19]^ |
| Ru/P-MoB | 40 | 43 | 24 | 3.6 | 0.811 | 1.59 | ^[20]^ |

**Table S6.** HER performance comparison of as-prepared Ir cluster@CoO/CeO_2_ with other typical electrocatalysts under neutral medium.

| **Catalyst** | Activity  (η_10_, mV) | Tafel slope  (mV dec^-1^) | C_dl_  (mF cm^-2^) | TOF  (s^-1^) | R_ct_  (Ω) | Stability  (h) | Ref. |
| --- | --- | --- | --- | --- | --- | --- | --- |
| Ir cluster@CoO/CeO_2_ | 52 | 83 | 10.4 | 3.4 | 3.3 | 300 | This work |
| Cu-CoP NAs/CP | 81 | 83.5 | 17 | 0.2 | 4 | 20 | ^[21]^ |
| Ni_0.1_Co_0.9_P | 125 | 103 | 11.3 | 0.25 | 2 | 20 | ^[9]^ |
| NiS_x_-24 h | 250 | 97.4 | -- | 4 | 3.75 | 12 | ^[7]^ |
| RuSe_x_-RuNC | 29 | 53 | 40.3 | -- | 2.1 | 100 | ^[22]^ |
| Ni_2_P-Ni_12_P_5_@Ni_3_S_2_/NF | 34 | 10.7 | 0.637 | -- | 24.88 | 24 | ^[23]^ |
| Pt_3_Fe/NMCS-A | 48 | 58 | 19.37 | 0.75 | -- | 10 | ^[18]^ |

**Table S7.** HER performance comparison of as-prepared Ir cluster@CoO/CeO_2_ with other typical electrocatalysts under alkaline medium.

| **Catalyst** | Activity  (η_10_, mV) | Tafel slope  (mV dec^-1^) | C_dl_  (mF cm^-2^) | TOF^a^  (s^-1^) | R_ct_  (Ω) | Stability  (h) | Ref. |
| --- | --- | --- | --- | --- | --- | --- | --- |
| Ir cluster  @CoO/CeO_2_ | 54 | 91 | 33.5 | 4.6 | 4.5 | 300 | This work |
| Pd_2_RuO_x_-0.5 h | 14 | 25.6 | 22.8 | 2.3 | 8 | 20 | ^[15]^ |
| Pt@S-NiFe LDH | 16 | 57.3 | 48.5 | 1.3 | 0.8 | 200 | ^[24]^ |
| Ir@CON | 13.5 | 29 | 2.83 | 0.2 | 2.54 | 75 | ^[25]^ |
| Ru/OMSNNC | 13 | 40.41 | 71.13 | 5.9 | 2 | 20 | ^[26]^ |
| Rh(OH)_3_/CoP | 13 | 24 | 118 | 20 | 1.6 | 25 | ^[19]^ |

**Table S8.** Water electrolysis performance comparison of as-prepared Ir cluster@CoO/CeO_2_ with other typical electrocatalysts under acidic medium.

| Catalyst | Current density  (mA cm^-2^) | Cell voltage (V) | Stability  (h) | Ref. |
| --- | --- | --- | --- | --- |
| Ir cluster@CoO/CeO_2_ | 10 | 1.49 | 300 | This work |
| IrCo@CNT/CC | 10 | 1.5 | 90 | ^[27]^ |
| Ir@Ni-NDC | 10 | 1.54 | 35 | ^[28]^ |
| IrO_2_/V_2_O_5_ | 10 | 1.5 | 30 | ^[16]^ |
| CoSAs-MoS_2_/TiN NRs | 10 | 1.7 | 30 | ^[29]^ |
| Co-RuIr | 10 | 1.52 | 25 | ^[30]^ |
| Ru@MoO(S)_3_ | 10 | 1.522 | 24 | ^[31]^ |
| Ru/Co-N-C | 10 | 1.5 | 20 | ^[32]^ |
| BPIr_be | 10 | 1.57 | 15 | ^[33]^ |
| Ir-NR/C | 10 | 1.55 | 12 | ^[34]^ |
| Ir-SA@Fe@NCNT | 10 | 1.51 | 12 | ^[1]^ |
| Ir-C | 10 | 1.495 | 8 | ^[35]^ |

**Table S9.** Water electrolysis performance comparison of as-prepared Ir cluster@CoO/CeO_2_ with other typical electrocatalysts under neutral medium.

| Catalyst | Current density  (mA cm^-2^) | Cell voltage (V) | Stability  (h) | Ref. |
| --- | --- | --- | --- | --- |
| Ir cluster@CoO/CeO_2_ | 10 | 1.67 | 300 | This work |
| karst N | 10 | 1.93 | 200 | ^[36]^ |
| CoO/Co_4_N/NF | 10 | 1.79 | 50 | ^[37]^ |
| IrO_2_/V_2_O_5_ | 10 | 1.65 | 30 | ^[16]^ |
| CoSAs-MoS_2_/TiN | 10 | 1.66 | 30 | ^[29]^ |
| S-NiFe_2_O_4_/NF | 10 | 1.95 | 24 | ^[38]^ |
| Cu-CoPNAs/CP | 10 | 1.72 | 20 | ^[21]^ |
| Ni_0.1_Co_0.9_P | 10 | 1.81 | 20 | ^[9]^ |
| Ir_0.5_W_0.5_ | 10 | 1.73 | 16.7 | ^[39]^ |
| Ir-NR/C | 10 | 1.7 | 12 | ^[34]^ |
| Ir-Ni(OH)_2_/NF | 10 | 1.64 | 12 | ^[40]^ |
| CoO/CoSe_2_ | 10 | 2.18 | 10 | ^[41]^ |

**Table S10.** Water electrolysis performance comparison of as-prepared Ir cluster@CoO/CeO_2_ with other typical electrocatalysts under alkaline medium.

| Catalyst | Current density  (mA cm^-2^) | Cell voltage (V) | Stability  (h) | Ref. |
| --- | --- | --- | --- | --- |
| Ir cluster@CoO/CeO_2_ | 10 | 1.52 | 300 | This work |
| Pd/NiFeOx | 10 | 1.57 | 50 | ^[42]^ |
| CoSAs-MoS_2_/TiN | 10 | 1.65 | 30 | ^[29]^ |
| Ru@MoO(S)_3_ | 10 | 1.526 | 24 | ^[31]^ |
| Ir/Ni_3_Fe/rGO | 10 | 1.57 | 24 | ^[43]^ |
| Ir_0.5_W_0.5_ | 10 | 1.6 | 16.7 | ^[39]^ |
| BPIr_be | 10 | 1.54 | 15 | ^[33]^ |
| Ir-NR/C | 10 | 1.57 | 12 | ^[34]^ |
| Ir-Ni(OH)_2_/NF | 10 | 1.54 | 12 | ^[40]^ |
| IrO_2_-RuO_2_/C | 10 | 1.52 | 8.5 | ^[44]^ |
| IrCo@NC-850 | 10 | 1.62 | 2.8 | ^[45]^ |

**Table S11.** OER, HER, and OWS performance comparison of as-prepared Ir cluster@CoO/CeO_2_ with other Co, Ir based electrocatalysts under different electrolyte conditions.

| **Catalyst** | HER  (η_10_, mV) | | OER  (η_10_, mV) | | OWS  (V) | | Ref. | |  |
| --- | --- | --- | --- | --- | --- | --- | --- | --- | --- |
| Ir cluster@CoO/CeO_2_ | 54^a^ | | 243^a^ | | 1.52^a^ | | This work | |  |
|  | 52^b^ | | 390^b^ | | 1.67^b^ | |  |  |  |
|  | 49^c^ | | 215^c^ | | 1.49^c^ | |  |  |  |
| Ru/Co-N-C | 19 | | 276 | | 1.49 | | ^[32]a^ | |  |
| Ir_VG | 17 | | 320 | | 1.57 | | ^[46]a^ | |  |
| Ir-NR/C | 42 | | 296 | | 1.57 | | ^[34]a^ | |  |
| Co SAs-MoS_2_/TiN NRs | 131.9 | | 340 | | 1.65 | | ^[29]a^ | |  |
| Ir@Ni-NDC | 31 | | 296 | | 1.46 | | ^[28]a^ | |  |
| Cu-CoP NAs/CP | 81 | | 411 | | 1.72 | | ^[21]b^ | |  |
| Co SAs-MoS_2_/TiN NRs | 203 | | 508 | | 1.65 | | ^[29]b^ | |  |
| Ni_0.1_Co_0.9_P | 125 | | 520 | | 1.81 | | ^[47]b^ | |  |
| IrO_2_/V_2_O_5_ | 147 | | 238 | | 1.52 | | ^[16]b^ | |  |
| BPIr_be | 330 | | 329 | | 1.65 | | ^[33]b^ | |  |
| Ru/Co-N-C | | 17 | | 323 | | 1.50 | | ^[32]c^ | |
| Ir_VG | | 47 | | 300 | | 1.58 | | ^[46]c^ | |
| Co SAs-MoS_2_/TiN NRs | | 187.5 | | 455 | | 1.7 | | ^[29]c^ | |
| Co-RuIr | | 14 | | 235 | | 1.52 | | ^[30]c^ | |
| IrCo@CNT/CC | | 26 | | 241 | | 1.50 | | ^[27]c^ | |

^a^ in 1.0 M KOH, ^b^ in 1.0 M PBS and ^c^ in 0.5 M H_2_SO_4_.

**Table S12.** Bader charge analysis

|  | Ir/ \|e\| | Co/ \|e\| | Ce/ \|e\| |
| --- | --- | --- | --- |
| Ir cluster@CoO/CeO_2_ | -0.10 | 0.87 | 2.25 |
| Ir cluster@CoO | -0.07 | 0.83 |  |
| CoO/CeO_2_ |  | 1.03 | 2.24 |

**References**

[1] F. Luo, H. Hu, X. Zhao, Z. Yang, Q. Zhang, J. Xu, T. Kaneko, Y. Yoshida, C. Zhu, W. Cai, *Nano Letters* **2020**, 20, 2120.

[2] H. Su, W. Zhou, W. Zhou, Y. Li, L. Zheng, H. Zhang, M. Liu, X. Zhang, X. Sun, Y. Xu, F. Hu, J. Zhang, T. Hu, Q. Liu, S. Wei, *Nature Communications* **2021**, 12, 6118.

[3] Y. Zhu, J. Wang, T. Koketsu, M. Kroschel, J.-M. Chen, S.-Y. Hsu, G. Henkelman, Z. Hu, P. Strasser, J. Ma, *Nature Communications* **2022**, 13, 7754.

[4] J. Kwon, S. Sun, S. Choi, K. Lee, S. Jo, K. Park, Y. K. Kim, H. B. Park, H. Y. Park, J. H. Jang, H. Han, U. Paik, T. Song, *Advanced Materials* **2023**, 35, 2300091.

[5] J. Yin, J. Jin, M. Lu, B. Huang, H. Zhang, Y. Peng, P. Xi, C.-H. Yan, *Journal of the American Chemical Society* **2020**, 142, 18378.

[6] M. Han, N. Wang, B. Zhang, Y. Xia, J. Li, J. Han, K. Yao, C. Gao, C. He, Y. Liu, Z. Wang, A. Seifitokaldani, X. Sun, H. Liang, *ACS Catalysis* **2020**, 10, 9725.

[7] G. Bahuguna, A. Cohen, B. Filanovsky, F. Patolsky, *Advanced Science* **2022**, 9, 2203678.

[8] N. Wang, P. Ou, S.-F. Hung, J. E. Huang, A. Ozden, J. Abed, I. Grigioni, C. Chen, R. K. Miao, Y. Yan, J. Zhang, Z. Wang, R. Dorakhan, A. Badreldin, A. Abdel-Wahab, D. Sinton, Y. Liu, H. Liang, E. H. Sargent, *Advanced Materials* **2023**, 35, 2210057.

[9] R. Wu, B. Xiao, Q. Gao, Y.-R. Zheng, X.-S. Zheng, J.-F. Zhu, M.-R. Gao, S.-H. Yu, *Angewandte Chemie International Edition* **2018**, 57, 15607.

[10] J. Wei, H. Tang, L. Sheng, R. Wang, M. Fan, J. Wan, Y. Wu, Z. Zhang, S. Zhou, J. Zeng, *Nature Communications* **2024**, 15, 559.

[11] Z. Zhang, C. Feng, D. Wang, S. Zhou, R. Wang, S. Hu, H. Li, M. Zuo, Y. Kong, J. Bao, J. Zeng, *Nature Communications* **2022**, 13, 2473.

[12] F. He, Q. Zheng, X. Yang, L. Wang, Z. Zhao, Y. Xu, L. Hu, Y. Kuang, B. Yang, Z. Li, L. Lei, M. Qiu, J. Lu, Y. Hou, *Advanced Materials* **2023**, 35, 2304022.

[13] J. Balamurugan, P. M. Austeria, J. B. Kim, E.-S. Jeong, H.-H. Huang, D. H. Kim, N. Koratkar, S. O. Kim, *Advanced Materials* **2023**, 35, 2302625.

[14] M. Liu, K.-A. Min, B. Han, L. Y. S. Lee, *Advanced Energy Materials* **2021**, 11, 2101281.

[15] V.-H. Do, P. Prabhu, V. Jose, T. Yoshida, Y. Zhou, H. Miwa, T. Kaneko, T. Uruga, Y. Iwasawa, J.-M. Lee, *Advanced Materials* **2023**, 35, 2208860.

[16] X. Zheng, M. Qin, S. Ma, Y. Chen, H. Ning, R. Yang, S. Mao, Y. Wang, *Advanced Science* **2022**, 9, 2104636.

[17] Z.-J. Wang, M.-X. Li, J.-H. Yu, X.-B. Ge, Y.-H. Liu, W.-H. Wang, *Advanced Materials* **2020**, 32, 1906384.

[18] P. Kuang, Z. Ni, B. Zhu, Y. Lin, J. Yu, *Advanced Materials* **2023**, 35, 2303030.

[19] M. Xing, S. Zhu, X. Zeng, S. Wang, Z. Liu, D. Cao, *Advanced Energy Materials* **2023**, 13, 2302376.

[20] P. Yang, F. Liu, X. Zang, L. Xin, W. Xiao, G. Xu, H. Li, Z. Li, T. Ma, J. Wang, Z. Wu, L. Wang, *Advanced Energy Materials* **2024**, 14, 2303384.

[21] L. Yan, B. Zhang, J. Zhu, Y. Li, P. Tsiakaras, P. Kang Shen, *Applied Catalysis B: Environmental* **2020**, 265, 118555.

[22] K. Sun, X. Wu, Z. Zhuang, L. Liu, J. Fang, L. Zeng, J. Ma, S. Liu, J. Li, R. Dai, X. Tan, K. Yu, D. Liu, W.-C. Cheong, A. Huang, Y. Liu, Y. Pan, H. Xiao, C. Chen, *Nature Communications* **2022**, 13, 6260.

[23] H. Yang, P. Guo, R. Wang, Z. Chen, H. Xu, H. Pan, D. Sun, F. Fang, R. Wu, *Advanced Materials* **2022**, 34, 2107548.

[24] H. Lei, Q. Wan, S. Tan, Z. Wang, W. Mai, *Advanced Materials* **2023**, 35, 2208209.

[25] J. Mahmood, M. A. R. Anjum, S.-H. Shin, I. Ahmad, H.-J. Noh, S.-J. Kim, H. Y. Jeong, J. S. Lee, J.-B. Baek, *Advanced Materials* **2018**, 30, 1805606.

[26] Y.-L. Wu, X. Li, Y.-S. Wei, Z. Fu, W. Wei, X.-T. Wu, Q.-L. Zhu, Q. Xu, *Advanced Materials* **2021**, 33, 2006965.

[27] X. Wang, Z. Qin, J. Qian, L. Chen, K. Shen, *ACS Catalysis* **2023**, 13, 10672.

[28] J. Yang, Y. Shen, Y. Sun, J. Xian, Y. Long, G. Li, *Angewandte Chemie International Edition* **2023**, 62, e202302220.

[29] T. L. L. Doan, D. C. Nguyen, S. Prabhakaran, D. H. Kim, D. T. Tran, N. H. Kim, J. H. Lee, *Advanced Functional Materials* **2021**, 31, 2100233.

[30] J. Shan, T. Ling, K. Davey, Y. Zheng, S.-Z. Qiao, *Advanced Materials* **2019**, 31, 1900510.

[31] D. Chen, R. Yu, D. Wu, H. Zhao, P. Wang, J. Zhu, P. Ji, Z. Pu, L. Chen, J. Yu, S. Mu, *Nano Energy* **2022**, 100, 107445.

[32] C. Rong, X. Shen, Y. Wang, L. Thomsen, T. Zhao, Y. Li, X. Lu, R. Amal, C. Zhao, *Advanced Materials* **2022**, 34, 2110103.

[33] J. Mei, T. He, J. Bai, D. Qi, A. Du, T. Liao, G. A. Ayoko, Y. Yamauchi, L. Sun, Z. Sun, *Advanced Materials* **2021**, 33, 2104638.

[34] F. Luo, L. Guo, Y. Xie, J. Xu, K. Qu, Z. Yang, *Applied Catalysis B: Environmental* **2020**, 279, 119394.

[35] Y. Peng, Q. Liu, B. Lu, T. He, F. Nichols, X. Hu, T. Huang, G. Huang, L. Guzman, Y. Ping, S. Chen, *ACS Catalysis* **2021**, 11, 1179.

[36] X. Gao, Y. Chen, T. Sun, J. Huang, W. Zhang, Q. Wang, R. Cao, *Energy & Environmental Science* **2020**, 13, 174.

[37] R.-Q. Li, P. Hu, M. Miao, Y. Li, X.-F. Jiang, Q. Wu, Z. Meng, Z. Hu, Y. Bando, X.-B. Wang, *Journal of Materials Chemistry A* **2018**, 6, 24767.

[38] J. Liu, D. Zhu, T. Ling, A. Vasileff, S.-Z. Qiao, *Nano Energy* **2017**, 40, 264.

[39] L. Fu, X. Hu, Y. Li, G. Cheng, W. Luo, *Nanoscale* **2019**, 11, 8898.

[40] Y. Tong, H. Mao, Q. Sun, P. Chen, F. Yan, J. Liu, *ChemCatChem* **2020**, 12, 5720.

[41] K. Li, J. Zhang, R. Wu, Y. Yu, B. Zhang, *Advanced Science* **2016**, 3, 1500426.

[42] W. Zhang, X. Jiang, Z. Dong, J. Wang, N. Zhang, J. Liu, G.-R. Xu, L. Wang, *Advanced Functional Materials* **2021**, 31, 2107181.

[43] Y. Li, Y.-C. Miao, C. Yang, Y.-X. Chang, Y. Su, H. Yan, S. Xu, *Chemical Engineering Journal* **2023**, 451, 138548.

[44] R. Samanta, P. Panda, R. Mishra, S. Barman, *Energy & Fuels* **2022**, 36, 1015.

[45] Y.-Q. Zhou, L. Zhang, H.-L. Suo, W. Hua, S. Indris, Y. Lei, W.-H. Lai, Y.-X. Wang, Z. Hu, H.-K. Liu, S.-L. Chou, S.-X. Dou, *Advanced Functional Materials* **2021**, 31, 2101797.

[46] S. B. Roy, K. Akbar, J. H. Jeon, S.-K. Jerng, L. Truong, K. Kim, Y. Yi, S.-H. Chun, *Journal of Materials Chemistry A* **2019**, 7, 20590.

[47] R. Wu, B. Xiao, Q. Gao, Y.-R. Zheng, X.-S. Zheng, J.-F. Zhu, M.-R. Gao, S.-H. Yu, *Angewandte Chemie International Edition* **2018**, 57, 15445.
